# SERCA2 regulates proinsulin processing and processing enzyme maturation in pancreatic beta cells

**DOI:** 10.1007/s00125-023-05979-4

**Published:** 2023-08-04

**Authors:** Hitoshi Iida, Tatsuyoshi Kono, Chih-Chun Lee, Preethi Krishnan, Matthew C. Arvin, Staci A. Weaver, Timothy S. Jarvela, Renato C. S. Branco, Madeline R. McLaughlin, Robert N. Bone, Xin Tong, Peter Arvan, Iris Lindberg, Carmella Evans-Molina

**Affiliations:** 1 Department of Medicine, Indiana University School of Medicine, Indianapolis, IN USA; 2 Department of Metabolism & Endocrinology, Juntendo University Graduate School of Medicine, Tokyo, Japan; 3 Department of Pediatrics, Indiana University School of Medicine, Indianapolis, IN USA; 4 Center for Diabetes and Metabolic Diseases, Indiana University School of Medicine, Indianapolis, IN USA; 5 Herman B. Wells Center for Pediatric Research, Indiana University School of Medicine, Indianapolis, IN USA; 6 Richard L. Roudebush VA Medical Center, Indianapolis, IN USA; 7 Department of Biochemistry and Molecular Biology, Indiana University School of Medicine, Indianapolis, IN USA; 8 Department of Anatomy and Neurobiology, University of Maryland School of Medicine, Baltimore, MD USA; 9 Weldon School of Biomedical Engineering, Purdue University, West Lafayette, IN USA; 10 Department of Molecular Physiology and Biophysics, Vanderbilt University, Nashville, TN USA; 11 Division of Metabolism, Endocrinology & Diabetes, University of Michigan Medical School, Ann Arbor, MI USA; 12 Department of Anatomy, Cell Biology, and Physiology, Indiana University School of Medicine, Indianapolis, IN USA

**Keywords:** Beta cell biology, Calcium imaging, Calcium signalling, Endoplasmic reticulum, Insulin secretion, Insulin synthesis, Proinsulin processing, Protein trafficking

## Abstract

**Aims/hypothesis:**

Increased circulating levels of incompletely processed insulin (i.e. proinsulin) are observed clinically in type 1 and type 2 diabetes. Previous studies have suggested that Ca^2+^ signalling within beta cells regulates insulin processing and secretion; however, the mechanisms that link impaired Ca^2+^ signalling with defective insulin maturation remain incompletely understood.

**Methods:**

We generated mice with beta cell-specific sarcoendoplasmic reticulum Ca^2+^ ATPase-2 (SERCA2) deletion (βS2KO mice) and used an INS-1 cell line model of SERCA2 deficiency. Whole-body metabolic phenotyping, Ca^2+^ imaging, RNA-seq and protein processing assays were used to determine how loss of SERCA2 impacts beta cell function. To test key findings in human model systems, cadaveric islets were treated with diabetogenic stressors and prohormone convertase expression patterns were characterised.

**Results:**

βS2KO mice exhibited age-dependent glucose intolerance and increased plasma and pancreatic levels of proinsulin, while endoplasmic reticulum (ER) Ca^2+^ levels and glucose-stimulated Ca^2+^ synchronicity were reduced in βS2KO islets. Islets isolated from βS2KO mice and SERCA2-deficient INS-1 cells showed decreased expression of the active forms of the proinsulin processing enzymes PC1/3 and PC2. Additionally, immunofluorescence staining revealed mis-location and abnormal accumulation of proinsulin and proPC2 in the intermediate region between the ER and the Golgi (i.e. the ERGIC) and in the cis-Golgi in beta cells of βS2KO mice. Treatment of islets from human donors without diabetes with high glucose and palmitate concentrations led to reduced expression of the active forms of the proinsulin processing enzymes, thus phenocopying the findings observed in βS2KO islets and SERCA2-deficient INS-1 cells. Similar findings were observed in wild-type mouse islets treated with brefeldin A, a compound that perturbs ER-to-Golgi trafficking.

**Conclusions/interpretation:**

Taken together, these data highlight an important link between ER Ca^2+^ homeostasis and proinsulin processing in beta cells. Our findings suggest a model whereby chronic ER Ca^2+^ depletion due to SERCA2 deficiency impairs the spatial regulation of prohormone trafficking, processing and maturation within the secretory pathway.

**Data availability:**

RNA-seq data have been deposited in the Gene Expression Omnibus (GEO; accession no.: GSE207498).

**Graphical Abstract:**

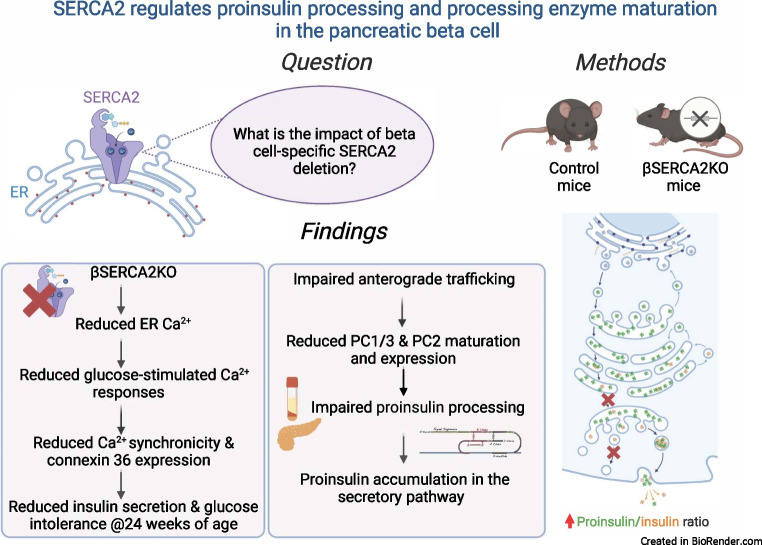

**Supplementary Information:**

The online version contains peer-reviewed but unedited supplementary material available at 10.1007/s00125-023-05979-4.


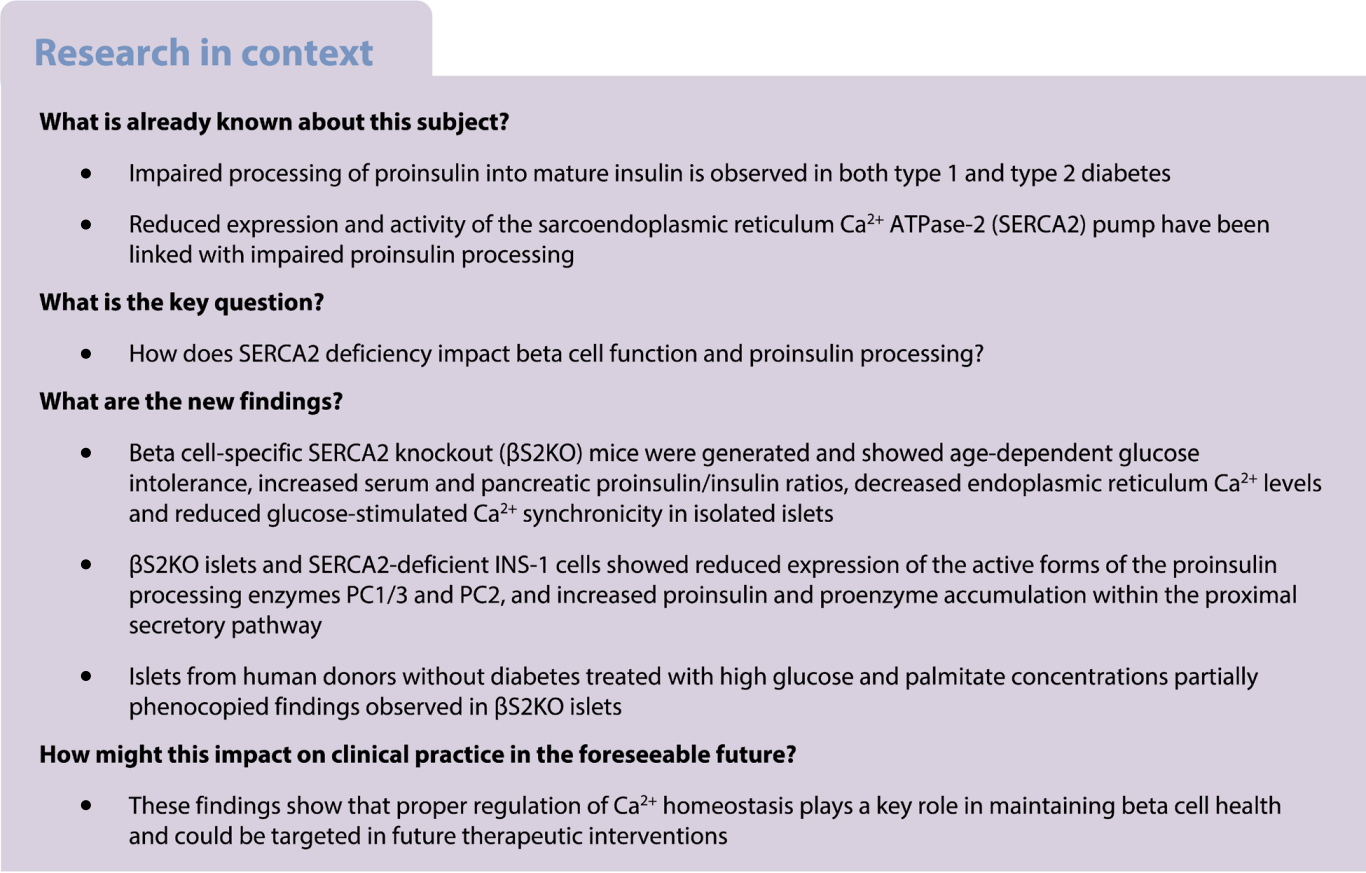


## Introduction

Loss of coordinated insulin production and secretion from pancreatic beta cells is a key pathological feature of both type 1 and type 2 diabetes. Insulin production is an energetically consuming process that involves translation of preproinsulin mRNA on the rough endoplasmic reticulum (ER) and co-translational insertion of the nascent preproinsulin molecule into the ER lumen, where the N-terminal signal peptide is cleaved to form proinsulin [1–3]. Following disulphide bond formation and terminal protein folding within the ER and Golgi complex, proinsulin is packaged into immature secretory granules and routed to the regulated secretory pathway within the trans-Golgi network (TGN) [4]. The final steps of proinsulin processing and maturation involve cleavage of intact proinsulin into mature insulin and C-peptide by proteolytic enzymes, including prohormone convertase 1/3 (PC1/3, encoded by the *PCSK1* gene), prohormone convertase 2 (PC2, encoded by *PCSK2*) and carboxypeptidase E (CPE, encoded by *CPE*), in a sequential process that begins in the TGN and is completed within the granule lumen. Secretion of mature insulin and C-peptide in response to glucose and other nutrient cues is a multistage process in which insulin-containing vesicles are transported to the plasma membrane for priming, docking and fusion during exocytosis [5].

Under a variety of stress and disease conditions, including overnutrition or genetic predisposition, the protein processing capacity within the beta cell secretory compartment is overwhelmed, leading to the accumulation of inadequately processed proinsulin [6–9]. Importantly, aberrant accumulation of proinsulin relative to insulin is observed in islets from pancreatic tissue from human organ donors with autoantibody positivity, type 1 diabetes, and type 2 diabetes [7, 10–12]. Clinically, this defect in protein processing and secretion manifests as an elevated ratio of secreted proinsulin/C-peptide or proinsulin/insulin (PI/I), which can be detected in the serum or plasma prior to the onset of both type 1 and type 2 diabetes, with persistence of this phenotype in established disease [13–19]. We and others have shown the presence of impaired proinsulin processing in ex vivo models of diabetes and metabolic stress [6, 8, 9, 20–22]; however, the molecular pathways responsible for defective proinsulin processing in vivo during the development of diabetes remain poorly understood.

Ca^2+^ depletion in the ER is a common pathway activated by multiple metabolic stressors in both type 1 and type 2 diabetes. Under normal conditions, steady-state ER Ca^2+^ levels are maintained through the balance between Ca^2+^ transport into the ER lumen by the sarcoendoplasmic reticulum Ca^2+^ ATPase (SERCA) pump and Ca^2+^ release via inositol trisphosphate receptors (IP3Rs) and ryanodine receptors [23–26]. We have shown previously that SERCA2 expression and activity are reduced in mouse models of diabetes and that mice with SERCA2 haploinsufficiency show impaired glucose tolerance, reduced insulin secretion, defective insulin granule maturation and increased proinsulin secretion when challenged with a high-fat diet [26–28]. In this study we generated beta cell-specific SERCA2-null C57BL/6J mice and used a SERCA2-null rat insulinoma cell line to investigate how loss of SERCA2 and chronic ER Ca^2+^ depletion impacts proinsulin processing.

## Methods

Details of key resources used in this study are provided in ESM Table 1.

### Animals

Male B6(Cg)-*Ins1*^*tm1.1(cre)Thor*^/J mice (*Ins1*^*cre*^) on a C57BL/6J background (MGI:5614359) were purchased from the Jackson Laboratory (https://www.jax.org/strain/026801). *Atp2a2*^*tm1.1Iemr*^ mice on a 129P2/OlaHsd background (MGI:4415164) were a kind gift from O. M. Sejersted (University of Oslo, Norway). *Atp2a2*^*tm1.1Iemr*^ mice were backcrossed for more than ten generations onto a C57BL6/J background, and beta cell-specific SERCA2 knockout (βS2KO) mice were generated by crossing *Atp2a2*^*tm1.1Iemr*^ mice on a C57BL/6J background with *Ins1*^*tm1.1(cre)Thor*^ mice. Mice were studied between 4 and 25 weeks of age.

During our analysis of these mice, we became aware of a polymorphism in the *P2rx7* gene that impacts many 129 strain-originating transgenic mouse lines. To account for this passenger mutation, all comparisons in this study were made using *Atp2a2*^*fl/fl*^;*Ins1*^*Cre*^-negative mice as controls. Therefore, we are able to attribute our observed phenotype directly to SERCA2 deletion, rather than to an off-target effect of the *P2rx7* passenger mutation inherent to *Atp2a2*^*tm1.1Iemr*^ mice. The data on this polymorphism are available online [29].

Because female βS2KO mice showed normal glucose tolerance, islets from male mice were used for ex vivo analyses. Littermates were randomly assigned to experimental groups. Mice were group housed at 22±2°C under a 12h light–12h dark cycle (lights on 0700–1900), with ad libitum access to a rodent chow diet (Teklad 2018SX).

All protocols involving mice were approved by the Indiana University Institutional Animal Care and Use Committee (protocol no. 11384) and were preformed according to Animal Research: Reporting of In Vivo Experiments (ARRIVE) guidelines. Animal health was monitored daily by veterinary staff at the Indiana University School of Medicine, and all mice used for experiments showed normal health. Every effort was made to minimise suffering.

### Cell lines

The male INS-1 832/13 cell line is an established model for studying glucose-stimulated insulin biosynthesis and secretion of insulin [30]. The INS-1 cell line was generated by irradiation of a male rat insulinoma [31], and the INS-1 832/13 line was generated by transfecting INS-1 cells with a plasmid containing the human insulin gene under the cytomegalovirus promoter [32]. A SERCA2 knockout INS-1 cell line (S2KO) was generated by the Genome Engineering and Stem Cell Center (GESC) at Washington University (St. Louis, MO, USA) from wild-type (WT) INS-1 832/13 cells using clustered regularly interspaced short palindromic repeats (CRISPR)/Cas 9 technology [26]. S2KO and WT INS-1 cells were maintained at 37°C in an atmosphere supplemented with 5% CO_2_. The cells were grown in RPMI 1640 containing 11 mmol/l glucose supplemented with 10% (vol./vol.) FBS, 100 U/ml penicillin, 100 μg/ml streptomycin, 10 mmol/l HEPES, 2 mmol/l l-glutamine, 1 mmol/l sodium pyruvate and 50 μmol/l β-mercaptoethanol.

Mouse insulinoma (MIN6) cells were kindly provided by J. Miyazaki (Osaka University, Japan). The MIN6 cell line was generated by microinjecting the SV40 T antigen gene connected to the human insulin promoter into pronuclei of fertilised eggs of C57BL/6 mice. Insulinoma cells were obtained from adult mice at 13 weeks of age [33].

MIN6 cells were maintained at 37°C in an atmosphere supplemented with 5% CO_2_ in DMEM containing 25 mmol/l glucose supplemented with 10% (vol./vol.) FBS, 100 U/ml penicillin, 100 μg/ml streptomycin, 10 mmol/l HEPES, 2 mmol/l l-glutamine, 1 mmol/l sodium pyruvate and 50 μmol/l β-mercaptoethanol. Cell lines were regularly verified to be free of mycoplasma.

### Human tissue

Human cadaveric islets were obtained from the Integrated Islet Distribution Program (IIDP, City of Hope, Duarte, CA, USA). Donor characteristics are shown in ESM Table 2. Immediately on receipt, human pancreatic islets were placed in DMEM containing 5.5 mmol/l glucose, 10% (vol./vol.) FBS, 100 U/ml penicillin and 100 μg/ml streptomycin and incubated overnight at 37°C in an atmosphere containing 5% CO_2_ [34]. Human islets distributed by IIDP are from cadaveric organ donors from whom at least one other organ has been approved for transplantation; tissues are considered ‘exempt’ from Human Studies Approval.

### Mouse metabolic studies

Body weight and random blood glucose levels were measured every other week. Blood glucose levels were measured in whole blood collected from the tail vein using a Contour glucometer. GTTs were performed following i.p. administration of glucose (2 g/kg body weight) after 6 h of fasting, as described previously [26]. d-(+)-glucose was prepared by adding 2.5 g powdered glucose to 10 ml sterile water and letting it dissolve at 4°C overnight. Glucose-stimulated insulin secretion was determined in vivo by i.p. injection of glucose (2 g/kg body weight) after 6 h of fasting and collection of serum at 0 and 15 min after glucose injection. ITTs were performed after 3 h of fasting followed by i.p. administration of 0.5 mU/kg short-acting insulin (Humulin R). Insulin and proinsulin levels in serum and tissues were measured using ELISA kits. The rat/mouse proinsulin ELISA has no cross reactivity with insulin or C-peptide.

### Isolation of mouse islets and perifusion analysis

Mouse pancreatic islets were isolated following injection of collagenase into the common bile duct, excision and digestion of the pancreas, and retrieval of the islets using a Histopaque gradient [35]. To measure dynamic insulin secretion, hand-picked islets (50/chamber) were perifused using Krebs buffer containing 2.8 mmol/l glucose for 20 min, followed by Krebs buffer containing 16.7 mmol/l glucose for 30 min at a rate of 120 μl/min using the Biorep Perifusion System. Secreted insulin in the perifusion buffer was measured using a mouse insulin ELISA and results were normalised to DNA content, which was quantified using the DNeasy Blood & Tissue Kit. The stimulation index was calculated as the insulin level after glucose stimulation divided by the basal insulin level for the peak of phase 1 insulin secretion and phase 2 insulin secretion.

### Quantitative RT-PCR

Mouse islets, muscle, heart, liver and hypothalamus were washed twice with PBS and total RNA was extracted using the Qiagen RNeasy Micro kit. Total RNA was reverse-transcribed at 37°C for 1 h using 15 μg random hexamers, 0.5 mmol/l deoxynucleotide triphosphate, 5× first-strand buffer, 0.01 mmol/l dithiothreitol and 200 U of Moloney murine leukaemia virus reverse transcriptase in a final reaction volume of 20 μl [36]. The cDNA template was then diluted 1:5 with RNase-free water and subjected to quantitative real-time reverse transcription PCR (qRT-PCR) using the SensiFAST SYBR Lo-ROX kit and a QuantStudio 3 thermal cycler. Relative RNA levels were calculated against constitutively expressed β-actin mRNA using the comparative ${2}^{{-\Delta \Delta \mathrm{C}}_{\mathrm{t}}}$ method, as described previously [37]. Primer sequences are provided in ESM Table 1.

### Mouse islet Ca^2+^ imaging

Direct measurement of ER Ca^2+^ was performed as previously described [26, 38]. Briefly, isolated mouse islets were incubated for 24 h with an adenovirus encoding the ER-targeted D4ER Cameleon probe expressed under transcriptional control of the rat insulin promoter [25, 38]. Transduced islets were incubated for an additional 24 h and transferred for imaging to a glass-bottom plate containing Hanks’ balanced salt solution (HBSS) supplemented with 0.2% (vol./vol.) BSA, 1.0 mmol/l Mg^2+^ and 2.0 mmol/l Ca^2+^. For Förster resonance energy transfer (FRET) experiments, confocal images were acquired with an LSM 800 confocal imaging system mounted with an Ibidi stage top incubator. Imaging was performed using a 405 nm single excitation laser line and two hybrid fluorescence emission detectors set to 460–500 nm (CFP channel) and 515–550 nm (YFP channel). Time-series images (z-stacks) of two to three stage-registered fields were acquired over a period of 20 min. FRET ratios were calculated using Zen Blue edition version 2.3 software by dividing the intensity acquired in the acceptor channel (YFP; on 405 nm excitation) by the intensity acquired in the donor channel (CFP). For cytosolic Ca^2+^ imaging, islets were incubated in HBSS buffer supplemented with 2 mmol/l Ca^2+^ and loaded with the ratiometric Ca^2+^ indicator fura-2-acetoxymethylester (Fura-2 AM). Baseline measurements were performed at 5.5 mmol/l glucose and spontaneous intracellular Ca^2+^ transients were measured in response to 16.7 mmol/l glucose using a Zeiss Lightsheet Z.1 microscope, as previously reported [25, 38].

To analyse Ca^2+^ synchronicity, islets were transduced with a GCaMP6s-expressing adenovirus and stimulated with high glucose concentrations (16.7 mmol/l). Images were captured using a Zeiss LSM 800 microscope. Islet movement was corrected by registration in ImageJ (version 2.1.0/1.53c) [39]. Regions of interest (ROIs) were manually assigned to cells positive for GCaMP6s, and mean intensities of cell ROIs were measured in ImageJ over the duration of the experiment. Mean intensities were calculated for representative islets and plotted against time with the 95% CI. The mean GCaMP6s signal was calculated by taking the mean fluorescent intensity of the cell over the course of the experiment (FI_avg_) for each islet. The *z* score was calculated for each time point and each beta cell by subtracting the mean fluorescent intensity of the beta cell over the course of the experiment (FI_avg_) from the fluorescent intensity at each time point (FI_t_) and dividing by the SD (StDevFI_avg_) of the beta cell fluorescent intensity over the course of the experiment: *z* score = (FI_t_ - FI_avg_)/StDevFI_avg_.

The mean SD of the islet beta cell *z* score was calculated for each islet by taking the mean of the SDs of the beta cell *z* scores for each islet over the course of the experiment. Oscillations in Ca^2+^ were illustrated by displaying the calculated *z* score of beta cells within an islet. *z* scores >1.96 represent statistical elevations in the GCaMP6s signal (alpha ≤0.05).

### Immunoblotting

Immunoblotting was performed as previously described [28]. Approximately 1.5×10^5^ S2KO and WT INS-1 cells or 100–125 human or mouse islets were incubated in RPMI 1640 culture medium for 2 days (cells) or 1 day (islets). Mouse and human islets were treated with 0.5 mmol/l BSA-conjugated palmitate and 25 mmol/l glucose for 24 h to model glucolipotoxicity. S2KO and WT INS-1 cells were treated with an adenovirus encoding human SERCA2b for 24 h [40], 4 µmol/l brefeldin A (BFA) for 24 h or 10 µmol/l cycloheximide in the presence or absence of 10 µmol/l MG132 proteasome inhibitor for 9 h prior to protein extraction. Approximately 1.0×10^6^ WT MIN6 or WT INS-1 cells were seeded into six-well plates. A 1.5 ml centrifuge tube containing 9 µl of Lipofectamine RNAiMAX and 150 µL of Opti Mem I and a 1.5 ml centrifuge tube containing 3 µl of 10 µmol/l siRNA against SERCA2 and 150 µl of Opti-Mem I were prepared. The diluted siRNA was added to the diluted Lipofectamine RNAiMAX and incubated at room temperature for 5 min. Cells were then incubated with 250 µl of the mixed reagent for 48 h. After removal of the transfection medium, cells were incubated in regular medium for an additional 48 h prior to protein extraction.

In other experiments, S2KO and WT INS-1 cells were treated with BFA, thapsigargin (TG; a SERCA inhibitor) or tunicamycin (TM; a blocker of N-linked glycosylation) for up to 24 h prior to protein extraction. Following the specified treatment, cells and islets were washed with PBS and lysed with buffer containing 50 mmol/l TRIS (pH 8.0), 150 mmol/l NaCl, 0.05% (wt/vol.) deoxycholate, 0.1% (vol./vol.) IGEPAL CA-630, 0.1% (wt/vol.) SDS, 0.2% (vol./vol.) sarcosyl, 10% (vol./vol.) glycerol, 1 mmol/l dithiothreitol, 1 mmol/l EDTA, 10 mmol/l sodium flouride, 1× EDTA-free complete protease inhibitor cocktail, 1× PhosSTOP, 2 mmol/l MgCl_2_ and 0.05% (vol./vol.) benzonase nuclease.

Mouse muscle, heart and liver were also homogenised and lysed using the same buffer and assayed for SERCA2. Anti-proPC2 and PC2 antisera were raised against residues within the propeptide of mouse pro-PC2 or the N-terminus of mature PC2, as previously reported [41].

Protein concentration was measured using the DC (detergent-compatible) protein assay kit II and a SpectraMax M5 multiwell plate reader. Equal quantities of proteins were suspended in 10% SDS and subjected to electrophoresis on a 4–20% Mini-Protean TGX gel in a Mini-Protean Tetra apparatus. Proteins were transferred to a PDVF membrane and the membrane was blocked with Odyssey blocking buffer and incubated with primary antibodies in Signal Enhancer Hikari solution 1 at 4°C overnight. Primary antibodies were diluted 1:1000, except for beta-actin, which was diluted 1:10,000. Antibodies against PC1/3 (rabbit, cat. no. 11914; RRID:AB_2631284), PC2 (rabbit, cat. no. 14013; RRID:AB_2631285) and insulin (rabbit, cat. no. 3014, RRID:AB_2126503) were from Cell Signaling Technology. The antibody against CPE (rabbit, cat. no. ab11044; RRID:AB_297698) was from Abcam. Antibodies against SERCA2 (goat, cat. no. sc-8095; RRID:AB_2290108), SERCA2 in ESM Figs 4 and 5 (mouse, cat. no. sc-376235; RRID:AB_10989947) and actin in ESM Figs 4 and 5 (mouse, cat. no. sc-47778; RRID:AB_626632) were from Santa Cruz Biotechnology. The antibody against actin (mouse, cat. no. MAB1501; RRID:AB_2223041) was from Millipore. Antibodies against the C-terminus of PC1/3 (rabbit) and proPC2 were a kind gift from I. Lindberg (University of Maryland, College Park, MD, USA; http://thelindberglab.com/antibodies/). Bound primary antibodies were detected with anti-mouse donkey antibody (1:10,000 dilution), anti-goat donkey antibody (1:10,000 dilution) or anti-rabbit donkey antibody (1:10,000 dilution) from LI-COR Biosciences [28]. Secondary antibodies were diluted in Signal Enhancer Hikari solution 2. Immunoblots were scanned using an Odyssey 1828 scanner and analysed using Image Studio software (version 3.1.4).

### Bulk RNA sequencing

Islets from three control and three βS2KO male mice were hand-picked and incubated overnight in RPMI 1640 supplemented with 10% (vol./vol.) FBS, 100 U/ml penicillin and 100 μg/ml streptomycin. RNA was isolated the following day using the RNeasy Micro kit and the quantity and quality of RNA were evaluated using the Bioanalyzer 2100. The cDNA library was prepared using 100 ng of total RNA and the KAPA mRNA Hyper Prep kit, according to the manufacturer’s protocol. For each generated indexed library, the quantity and quality were assessed using a Qubit Fluorometer and a Bioanalyzer 2100, respectively. Multiple libraries were pooled in equal molarity. The pooled libraries were denatured, neutralised and loaded onto a HiSeq 4000 sequencer at a final concentration of 300 pmol/l for 100 bp paired-end sequencing. Approximately 30 million reads per library were generated. The Phred quality score (*Q* score) was used to measure the quality of sequencing; more than 90% of the sequencing reads reached Q30 (99.9% base call accuracy). The quality of sequencing data was assessed using the FastQC tool (version 0.11.5).

### mRNA sequencing data analysis

Sequencing files were analysed using Flow software version 10.0.20.1231. The raw sequencing files were aligned to the mouse genome (mm10) using STAR aligner version 2.7.3a. Uniquely mapped reads were annotated using RefSeq release 93. mRNAs with a total of at least ten read counts were retained for further analysis. mRNAs with a linear-scale fold change (FC) ≥1.5 and *p*<0.05 were identified using DESeq2 and were considered differentially expressed mRNAs. Biological pathways were identified using Qiagen Ingenuity Pathway Analysis, and gene ontology enrichment was performed using Metascape. Functional terms with *p*<0.05 were considered significant. Figures were generated using the ggplot2 package in R and GraphPad Prism 7.0 software.

### Fluorogenic PC1/3 and PC2 enzyme activity assays

S2KO or WT INS-1 cells were transduced with an empty adenovirus or adenovirus expressing human SERCA2b (Ad-SERCA2, a kind gift from U. Ozcan, Harvard Medical School, Boston, MA, USA) at 1×10^7^ plaque-forming units/well [40]. At 48 h after transduction, cells were incubated for an additional 24 h in Opti-Mem I medium containing 11 mmol/l glucose, 100 U/ml penicillin, 100 μg/ml streptomycin, 10 mM HEPES, 2 mmol/l l-glutamine, 1 mmol/l sodium pyruvate, 50 μmol/ml β-mercaptoethanol and 1 mg/ml bovine aprotinin. Enzyme activity of secreted PC1/3 and PC2 was analysed in a reaction buffer containing 100 mmol/l sodium acetate (pH 5.5 for PC1/3; pH 5.0 for PC2), 2mmol/l CaCl_2_, 0.1% (vol./vol.) Triton X-100, 200 μmol/l pERTKR-aminomethyl-coumarin and a proteinase inhibitor cocktail containing 1 μmol/l pepstatin, 0.28 mmol/l N-*p*-tosyl-l-phenylalanine chloromethyl ketone, 10 μmol/l *trans*-epoxysuccinyl-l-leucylamido(4-guanidino) butane and 0.14 mmol/l Nα-tosyl-l-lysine chloromethyl ketone. To measure PC1/3 and PC2 activity, 7B2-CT peptide was used to inhibit PC2 activity in parallel reactions, while a similar concentration of the proSAAS-CT peptide was used to specifically inhibit PC1/3 activity (both a gift from I. Lindberg) [42]. Following incubation at 37°C for 3 h, the fluorescence was quantified using the SpectraMax ID5 plate reader operating at the excitation wavelength of 380 nm and the emission wavelength of 460 nm. Aminomethyl-coumarin was used as a standard [43, 44]. Enzyme activity was normalised to the amount of enzyme determined by immunoblotting; inhibitor-containing reactions were subtracted from non-inhibitor-containing reactions.

### Immunofluorescence and morphometric analysis

Mice were euthanised and pancreases were rapidly removed and fixed overnight in Z-Fix (buffered zinc formalin). Fixed specimens were paraffin-embedded and longitudinal sections measuring 5 μm in thickness were obtained [26]. Beta cell mass was estimated by multiplying the beta cell fractional area by the weight of the pancreas, as previously described [26]. After reducing non-specific binding by incubation for 30 min with Animal-Free Blocker followed by incubation for 1 h with Mouse-on-Mouse Blocking Solution (Vector Laboratories), sections were incubated with primary antibodies at 4°C overnight in a humidity chamber and then with AlexaFluor-conjugated secondary antibodies at room temperature for 1 h. Primary antibodies were diluted 1:100 in Animal-Free Blocker and Diluent (Vector Laboratories), except for the insulin antibody, which is a ready-to-use product that does not require dilution. The primary antibody against proinsulin (mouse, cat. no. GS-9A8-C) was from the Developmental Studies Hybridoma Bank. The antibody against insulin (guinea pig, cat. no. A0564; RRID:AB_10013624) was from Agilent Dako, and the antibody against giantin (goat, cat. no. sc-46993; RRID:AB_2279271) was from Santa Cruz Biotechnology. The antibody against proPC2 was a kind gift from I. Lindberg. The antibody against lectin mannose-binding 1 [LMAN1] (cat. no. ab125006; RRID:AB_10973984) was from Abcam, and the antibody against connexin-36 (rabbit, cat. no. NBP1-59254; RRID:AB_11039152) was from Novus Biological. Bound primary antibodies were detected with anti-mouse donkey antibody, anti-goat donkey antibody or anti-rabbit donkey antibodies from Thermo Fisher Scientific or with anti-guinea pig donkey antibody from Jackson ImmunoResearch Laboratories. Secondary antibodies were diluted 1:500 in Animal-Free Blocker and Diluent (Vector Laboratories). Specificities of primary antibodies were checked in negative control experiments without primary antibodies. Stained sections were mounted with FluorSave. Islet images were acquired using an LSM 800 confocal imaging system using 40× and 63× oil immersion objectives with an Airyscan detector. Images were analysed using Zen Blue edition software. Signal intensities were quantified using ImageJ. Puncta count and co-localisation analyses were performed using CellProfiler 4.1.3 [45]. The background was subtracted from each image by removing the lower-quartile intensity from each channel. Pancreas ROIs were defined as proinsulin-positive areas, and proinsulin, LMAN1, proPC2 and giantin puncta were identified by discarding objects outside a pixel diameter range after median filtering [46]. The puncta count was normalised to the proinsulin-positive area. For co-localisation positivity, parent (giantin or LMAN1) and child (proinsulin or proPC2) objects were defined [46], and the percentage of proinsulin or proPC2 puncta co-localised or co-compartmentalised with giantin or LMAN was determined using CellProfiler’s built-in tutorials.

### Quantification and statistical analysis

The definitions of *n*, mean and error bars and the statistical methods used are listed in the figure legends. Unless otherwise indicated, at least three independent experiments were carried out for each assay. Data are displayed as means ± SEM, and *p*<0.05 was considered to indicate a significant difference between groups. Data analysis was performed using GraphPad Prism 7.0 software. Pairwise comparisons were performed using unpaired Student’s *t* tests. Interactions between two groups with multiple treatments were determined using two-way ANOVA with a multiple comparisons post hoc test. Normal distribution was assessed using a Kolmogorov–Smirnov test and, when the *p* value was >0.05, normal data distribution was assumed. Statistical tests were two-tailed.

## Results

### Pancreatic beta cell-specific SERCA2 deletion results in age-dependent glucose intolerance and impaired insulin secretion

**Fig. 1:**
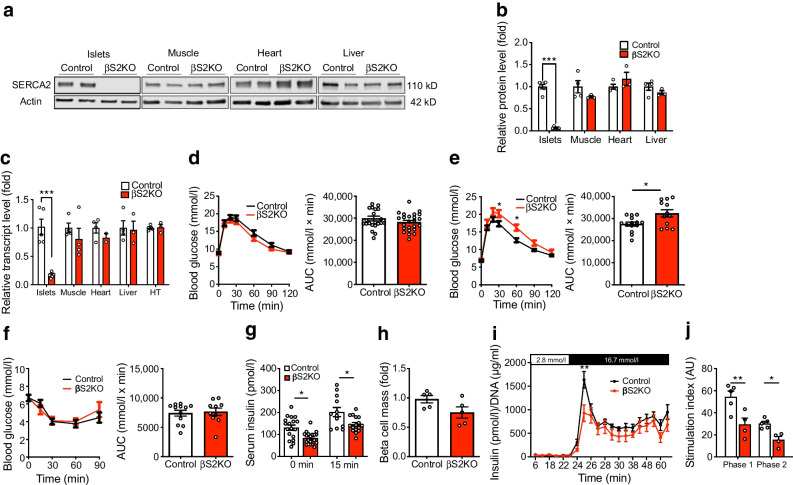
βS2KO mice exhibit age-dependent glucose intolerance and impaired insulin secretion without changes in insulin sensitivity. βS2KO and *Atp2a2*^*tm1.1Iemr*^ mice (control) were fed a normal chow diet for 25 weeks. (**a**) Representative immunoblot performed using SERCA2 and actin antibodies in tissues from control and βS2KO mice. (**b**) Quantification of immunoblotting results. Expression of SERCA2 was normalised to actin expression (*n*=3–6). (**c**) SERCA2 (*Atp2a2*) transcript levels in tissues of control and βS2KO mice were determined by qRT-PCR (*n*=3–6). HT, hypothalamus. (**d**, **e**) IPGTTs were performed in male control and βS2KO mice. Glucose (2 g/kg body weight) was administered after 6 h of fasting. The tests were performed at (**d**) 8 and (**e**) 24 weeks of age. Bar graphs show quantification of the AUC (*n*=12–24). (**f**) ITTs were performed in male control and βS2KO mice at 25 weeks of age following injection of short-acting insulin. Bar graph shows AUC quantification (*n*=10–12). (**g**) In vivo glucose-stimulated insulin secretion assays were performed in male control and βS2KO mice after 6 h of fasting and administration of glucose (2 g/kg body weight). Insulin levels were measured by ELISA at baseline and 15 min after glucose injection (*n*=12–20). (**h**) Pancreatic beta cell mass was assessed by insulin immunostaining in the pancreases of control and βS2KO mice (*n*=5). (**i**, **j**) Isolated islets from βS2KO and control mice were perifused with 2.8 mmol/l glucose (0–23 min) and 16.8 mmol/l glucose (23–74 min) (*n*=4–5). (**i**) Glucose-stimulated insulin secretion during the whole perifusion experiment. (**j**) The stimulation index was calculated as the insulin level after glucose stimulation divided by the basal insulin level for the peak of phase 1 insulin secretion and phase 2 insulin secretion. Results are presented as means ± SEM. Replicate samples are indicated by open circles. Comparisons between two groups were performed using unpaired Student’s *t* tests. Multiple comparisons were made using two-way ANOVA and Sidak’s post test. *p<0.05, **p<0.01, ***p<0.001 vs control

Over 14 different isoforms of SERCA have been identified and been found to show tissue-specific expression and alternative mRNA splicing patterns [47]. SERCA2 and SERCA3 are both expressed in beta cells, and we have identified SERCA2b as the most highly expressed isoform at the mRNA level [28]. Therefore, to determine the role of SERCA2 in beta cell biology, pancreatic beta cell-specific SERCA2 knockout mice (βS2KO) were generated by crossing *Atp2a2*^*tm1.1Iemr*^ mice on a C57BL/6J background with *Ins1*^*tm1.1(cre)Thor*^/J mice [48]. The efficiency of SERCA2 deletion in isolated islets was approximately 95% at the protein level (Fig. 1a,b) and 85% at the mRNA level (Fig. 1c). Protein and mRNA expression of SERCA2 remained unchanged in other tissues including the hypothalamus, heart, liver and skeletal muscle (Fig. 1a–c). βS2KO mice exhibited normal body weight gain, random glucose levels and lean mass on a chow diet, whereas fat mass was slightly increased compared with control mice (ESM Fig. 1a–d). While 8-week-old βS2KO male mice showed normal glucose tolerance when challenged with an i.p. glucose injection (Fig. 1d), they developed significant glucose intolerance by 24 weeks of age (Fig. 1e). There was no phenotype in female mice at the same age (ESM Fig. 1e–h). The glucose-intolerant phenotype of 24-week-old male βS2KO mice was not accompanied by changes in systemic insulin sensitivity (Fig. 1f), suggesting reduced beta cell function in βS2KO mice. In support of this conclusion, the serum insulin response to i.p. glucose injection was significantly lower in βS2KO mice than in control mice (Fig. 1g), with a non-significant reduction in beta cell mass (classically defined by anti-insulin immunopositivity) in βS2KO mice compared with control mice (0.75 mg vs 0.98 mg, *p*=0.08) at 24 weeks of age (Fig. 1h). Representative images are shown in ESM Fig. 1i.

To evaluate dynamic patterns of insulin secretion, perifusion experiments were performed using islets isolated from βS2KO and control mice. Overall, insulin secretion from βS2KO islets was lower than that from islets from control mice (Fig. 1i,j). Furthermore, stimulation index analysis showed a significant decrease in the peak of both phase 1 and phase 2 insulin secretion from βS2KO islets. Taken together, these findings show that SERCA2 deletion in beta cells results in glucose intolerance and impaired insulin secretion.

### Beta cell-specific SERCA2 deletion reduces ER Ca^2+^ levels and alters nutrient-induced Ca^2+^ signalling

**Fig. 2:**
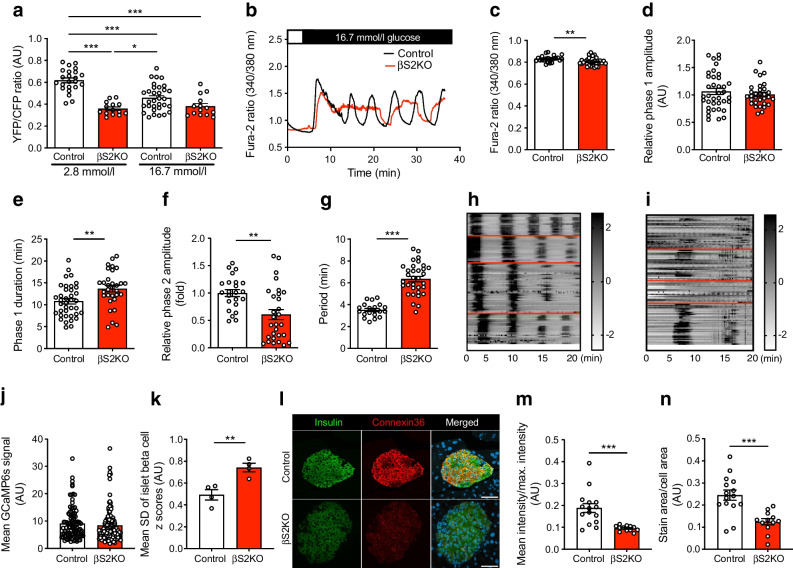
Islets from βS2KO mice exhibit reduced ER Ca^2+^ levels and altered glucose-stimulated Ca^2+^ oscillations. (**a**) Control and βS2KO islets were transduced with an adenovirus expressing the D4ER Cameleon probe under control of the rat insulin promoter. Images were captured before and after treatment with high glucose (16.7 mmol/l); ER Ca^2+^ levels are expressed as the YFP/CFP ratio (*n*=14–32 islets from three mice). (**b–g**) Control and βS2KO islets were loaded with Fura-2 AM and Ca^2+^ imaging was performed. (**b**) Representative recordings of cytosolic Ca^2+^ after stimulation with 16.7 mmol/l glucose. Quantification of the baseline (**c**) and mean phase 1 amplitude (**d**), phase 1 duration (**e**), phase 2 (oscillation) amplitude (**f**) and oscillation period (**g**) (*n*=20–38 islets from three mice). (**h–k**) Control and βS2KO islets were transduced with an adenovirus expressing GCaMP6s. (**h**, **i**) Example traces of phase 2 responses of GCaMP6-expressing islet cells treated with 16.7 mmol/l glucose. ROIs were manually assigned to individual cells positive for GCaMP6s. The *z* score was calculated for each time point and each beta cell as described in the methods. Oscillations in cytosolic Ca^2+^ were illustrated by displaying the calculated *z* score of each beta cell. Oscillatory patterns for individual islets are demarcated by red lines. Index bars on the right of the spectrograms show the *z* scores in greyscale. (**j**) The mean GCaMP6s signal in control (*n*=98) and βS2KO (*n*=112) beta cells was calculated and plotted. (**k**) Mean SD of *z* scores for each islet (*n*=4 per group) over the time course of the experiment. (**l**) Pancreatic sections from control and βS2KO mice were stained for insulin and connexin-36. Scale bar=50 μm. Quantification of image intensity (**m**) and stain area (**n**) were assessed using ImageJ (*n*=3). Results are presented as means ± SEM. Replicate samples are indicated by open circles. *p<0.05, **p<0.01, ***p<0.001 vs control islets by Student’s *t* test or (**a**) two-way ANOVA

To test whether SERCA2 deletion was sufficient to reduce ER Ca^2+^ levels, islets isolated from control and βS2KO mice were transduced with an adenovirus expressing the D4ER Cameleon probe under transcriptional control of the rat insulin promoter [49]; ER Ca^2+^ levels were measured by FRET. At basal glucose levels (2.8 mmol/l), the FRET ratio was higher in islets from control mice than in islets from βS2KO mice, indicating a significant reduction in ER Ca^2+^ levels in βS2KO islets. Treatment with high glucose (16.7 mmol/l) led to a reduction in the FRET ratio in control islets. In contrast, glucose treatment did not significantly change the FRET ratio in βS2KO islets (Fig. 2a).

Next, glucose-stimulated Ca^2+^ responses were assessed using the cytosolic Ca^2+^ dye Fura-2AM (Fig. 2b). Islets from βS2KO mice showed a slight but statistically significant reduction in baseline cytosolic Ca^2+^ levels (3.3% reduction vs control; Fig. 2c), an equivalent phase 1 amplitude (Fig. 2d), an increased phase 1 duration (26.3% increase vs control; Fig. 2e), a reduced amplitude of the second phase response (39.7% reduction vs control; Fig. 2f) and a longer oscillatory period (80% increase vs control; Fig. 2g). To measure glucose-induced Ca^2+^ oscillations in individual beta cells, isolated islets were transduced with an adenovirus expressing the cytosolic Ca^2+^ indicator GCaMP6 under control of the rat insulin promoter. Notably, beta cells within islets of βS2KO mice showed diminished synchronicity of Ca^2+^ oscillations, as shown by a significantly higher GCaMP6 signal variance among the beta cell population (Fig. 2h–k).

Ion coupling at gap junctions via connexin-36 is critically important for the coordination of Ca^2+^ oscillations and glucose-stimulated insulin secretion in pancreatic islets [50, 51]. Therefore, to examine the effect of SERCA2 deletion on connexin-36 expression, immunostaining was performed in pancreatic sections from control and βS2KO mice. Interestingly, we observed a reduction in connexin-36 staining intensity and area in islets from βS2KO mice (Fig. 2l–n).

### SERCA2 deficiency impairs proinsulin processing and processing enzyme maturation

**Fig. 3:**
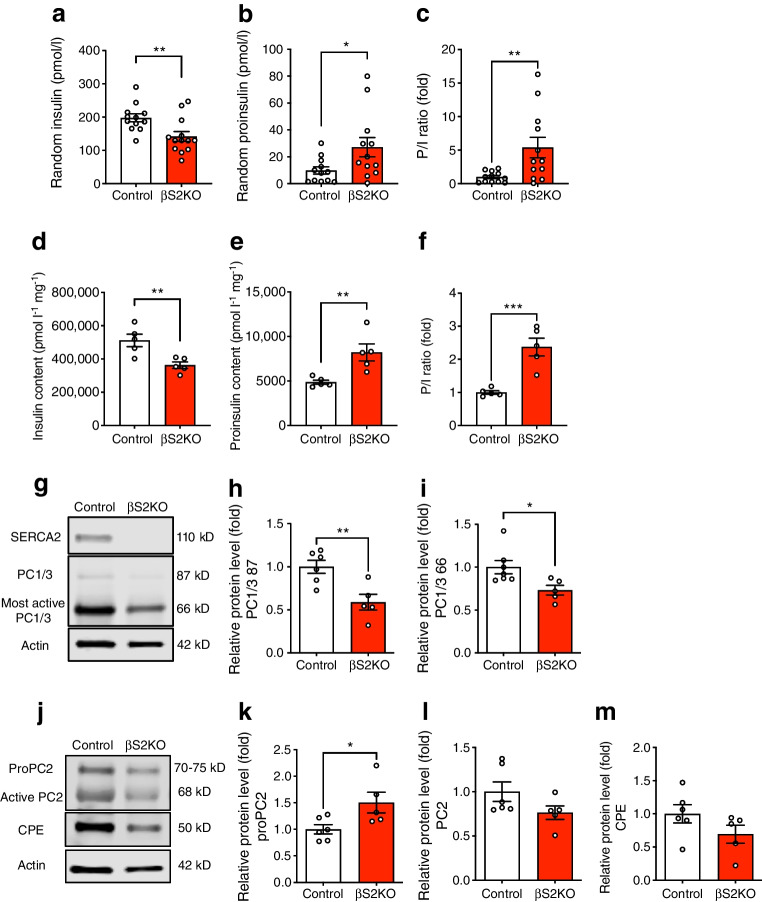
Beta cell-specific SERCA2 deficiency results in an increase in serum and total pancreas P/I ratios and alters the expression of prohormone convertase isoforms in islets. (**a–c**) Blood was collected from randomly fed control and βS2KO mice at 25 weeks of age and serum was isolated. Serum levels of insulin (**a**) and proinsulin (**b**) were determined by ELISA and the P/I ratio (**c**) was calculated (*n*=12–13 mice). (**d–f**) Total protein was extracted from the whole pancreas and the levels of insulin (**d**) and proinsulin (**e**) were determined by ELISA and the P/I ratio (**f**) was calculated (*n*=5). (**g**, **j**) Representative immunoblots performed using antibodies against SERCA2 and PC1/3 (**g**) and PC2 and CPE (**j**) in islets obtained from 24-week-old control and βS2KO mice. (**h**, **i**, **k–m**) Quantification of immunoblotting results: (**h**, **i**) PC1/3, (**k**) proPC2, (**l**) PC2 and (**m**) CPE. Expression of proteins was normalised to actin expression (*n*=4–7). Results are presented as means ± SEM. Replicate samples are indicated by open circles. *p<0.05, **p<0.01, ***p<0.001 vs control using Student’s *t* tests

To further investigate the mechanisms underlying the glucose-intolerant phenotype observed in βS2KO mice, serum insulin and proinsulin levels were measured. Serum insulin levels were significantly decreased, coupled with an increase in serum proinsulin levels and an elevated serum PI/I ratio in βS2KO mice (Fig. 3a–c). Consistent with these results, insulin content in the whole pancreas of βS2KO mice was decreased by ~25%, while the proinsulin content and the pancreas PI/I ratio were increased by 1.5 and 2-fold, respectively (Fig. 3d–f).

Given the prominent defect in proinsulin processing observed in our model, we analysed the effect of beta cell-specific SERCA2 deletion on the expression of the three main proinsulin processing enzymes, PC1/3, PC2 and CPE, which are synthesised as proenzymes and undergo sequential maturation and activation by autocleavage in different domains of the secretory pathway. First, the protein sizes of proPC2 and active PC2 in rat, mouse and human samples were confirmed by western blotting using previously validated antibodies [41, 52] targeted at the N-terminus of PC2 (ESM Fig. 2e) and the propeptide of proPC2 (ESM Fig. 2f), respectively. The band for proPC2 in INS1 cells and islets was detected at around 70–75 kDa, which is slightly smaller than previously reported in the mouse pituitary AtT-20 cell line [41].

Notably, expression of both the intermediate/less active (87 kDa) and the most active (66 kDa) forms of PC1/3 was significantly reduced in islets from βS2KO mice. Similarly, levels of the inactive form of PC2 (proPC2; 70–75 kDa) were increased in βS2KO islets. Levels of the active forms of PC2 and CPE were reduced but not to a significant extent (Fig. 3g–m). qRT-PCR analysis of islets showed no differences in mRNA levels of PC1/3, PC2 or CPE (ESM Fig. 2a–d) between control and βS2KO mice. Together, these results suggest that SERCA2 deletion in beta cells may impact insulin processing through impaired maturation of key proinsulin processing enzymes.

### Prohormone convertase enzyme activity is regulated in a SERCA2-dependent manner in beta cell lines

**Fig. 4:**
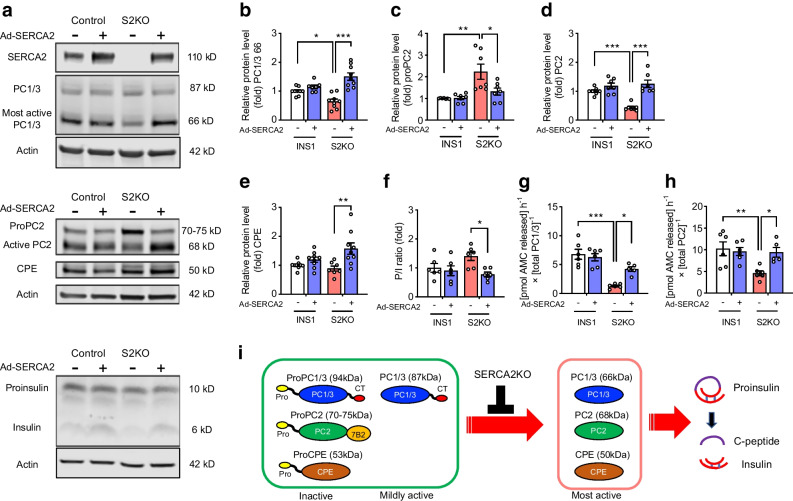
Enzyme activity of prohormone convertases is regulated in a SERCA2-dependent manner in beta cell lines. (**a**) Representative immunoblots of SERCA2, PC1/3, PC2, CPE, proinsulin and insulin in WT and S2KO cells transduced with empty adenovirus or an adenovirus expressing human SERCA2b (Ad-SERCA2). (**b–e**) Quantification of immunoblotting results: (**b**) PC1/3, (**c**) proPC2, (**d**) PC2 and (**e**) CPE. Expression of proteins was normalised to actin expression (*n*=6–9). (**f**) Quantification of the P/I ratio (*n*=6). (**g**, **h**) WT and S2KO INS-1 cells were transduced with empty adenovirus or Ad-SERCA2 and the enzymatic activity of secreted PC1/3 and PC2 was determined using fluorometric assays. Enzyme activity was normalised to the protein level of each enzyme, as determined by immunoblotting (*n*=5–6). AMC, aminomethyl-coumarin; CT, C-terminal. (**i**) Schematic illustration of the function of the S2KO in the maturation of prohormone convertases and insulin processing. Before becoming enzymatically capable of cleaving their targets, including proinsulin, precursors to PC1/3, PC2 and CPE must be activated through a series of autoproteolytic events, most of which are pH- and Ca^2+^-dependent. Results are presented as means ± SEM. Replicate samples are indicated by open circles. *p<0.05, **p<0.01, ***p<0.001 vs indicated groups by two-way ANOVA followed by Tukey's multiple comparison test

To confirm a cell-autonomous relationship between SERCA2 and insulin processing, we analysed prohormone convertase expression patterns in WT and SERCA2-deficient (S2KO) INS-1 cell lines [26]. Similar to results observed in islets from βS2KO mice (Fig. 3), S2KO cells showed reduced protein levels of the most active form of PC1/3 (66 kDa). In addition, S2KO cells exhibited increased levels of proPC2 and reduced levels of the active form of PC2 (68 kDa) (Fig. 4a–d). Adenoviral re-expression of SERCA2b in S2KO cells restored protein levels of the active forms of PC1/3, PC2 and CPE and decreased proPC2 levels and the PI/I ratio to levels seen in WT cells (Fig. 4a–f). Fluorogenic enzyme assays showed an 81% reduction in PC1/3 enzyme activity and a 55% reduction in PC2 enzyme activity in S2KO cells compared with WT cells, and the activity of both enzymes was partially rescued by adenoviral restoration of SERCA2 expression in S2KO cells (Fig. 4g,h). These results indicate that SERCA2 regulates prohormone convertase maturation and activation in the secretory pathway of beta cells (Fig. 4i).

### RNA-seq shows functional enrichment of genes involved in Ca^2+^ signalling, diabetes pathophysiology and secretory pathway function in islets from βS2KO mice

**Fig. 5:**
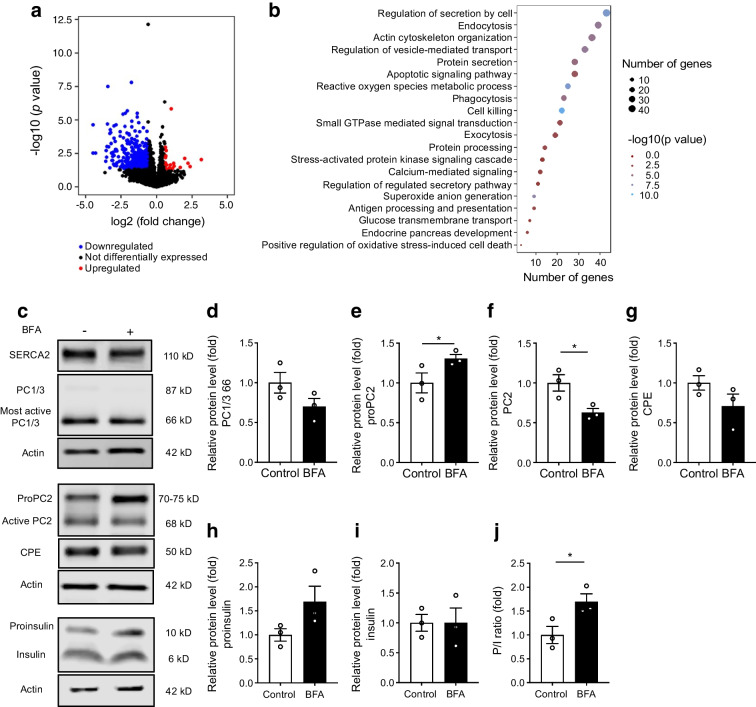
Bulk RNA-seq and chemical inhibition of vesicle trafficking demonstrate a role for SERCA2 in prohormone maturation. (**a**, **b**) RNA isolated from islets from 17-week-old control and βS2KO mice was subjected to bulk RNA-seq. (**a**) Volcano plot indicating differentially expressed genes (FC ≥1.5, *p*<0.05). Red indicates upregulated genes, blue indicates downregulated genes and black indicates no change in expression in βS2KO islets vs control islets. (**b**) Dot plot representing the top 20 most relevant GO terms identified using the ggplot2 package in R. The size of the dots indicates the number of genes in each term. Enrichment analysis was performed using Metascape. (**c–j**) Isolated WT mouse islets were treated with 4 µmol/l BFA for 6 h. (**c**) Representative immunoblots of PC1/3, proPC2, PC2, CPE, proinsulin and insulin. (**d–i**) Quantification of immunoblotting results: (**d**) PC1/3, (**e**) proPC2, (**f**) PC2, (**g**) CPE, (**h**) proinsulin and (**i**) insulin. Expression of proteins was normalised to actin expression and FC is relative to the mean control value (*n*=3). (**j**) Quantification of the P/I ratio; *n*=3. Results are presented as means ± SEM. Replicate samples are indicated by open circles. *p<0.05 vs control by Student’s *t* test

To gain insight into novel pathways through which beta cell SERCA2 modifies systemic glucose tolerance and proinsulin processing, RNA-seq was performed using islets isolated from 17-week-old control and βS2KO male mice, a time-period prior to the development of glucose intolerance. We identified 453 differentially expressed mRNAs, of which 22 were upregulated and 431 (95%) were downregulated in βS2KO mice (Fig. 5a; ESM Table 3). There was a non-significant decrease in *Ins1* and an increase in *Ins2* in the RNA-seq data, while qRT-PCR showed no significant changes in *Ins1* or *Ins2* in βS2KO islets isolated from 24-week-old mice (ESM Fig. 3). Pathway analysis identified 162 pathways that were significantly modulated and, after removing terms that are less relevant to islet cells and our study context, 112 pathways were retained (ESM Table 4). Gene Ontology (GO) terms were identified using Metascape, and terms with *p*<0.05 were considered significant (Fig. 5b; ESM Table 5). As expected, significantly altered GO terms included processes involved in Ca^2+^ signalling. Interestingly, this analysis also highlighted modulation of several pathways relevant to diabetes pathophysiology (‘apoptosis’, ‘antigen presentation’ and ‘oxidative stress’), with a notable enrichment of terms related to secretory function (‘exocytosis’, ‘protein secretion’, ‘protein processing’ and ‘regulation of vesicle-mediated transport’).

### SERCA2 loss disrupts protein trafficking within the beta cell secretory pathway

Enrichment for terms related to secretory pathway transport within the RNA-seq dataset was notable given our observations of reduced proinsulin processing and impaired maturation of proinsulin processing enzymes, both of which are spatially regulated within different domains of the beta cell secretory pathway. Thus, we hypothesised that chronic ER Ca^2+^ depletion within the beta cell secretory pathway may cause a proinsulin processing defect via impaired anterograde protein trafficking. With this in mind, we treated WT mouse islets with BFA, an inhibitor of anterograde protein transport. Similar to what was observed in SERCA2-deficient islets and cells, BFA treatment decreased active PC2 levels (68 kDa) and led to increased accumulation of proPC2 (Fig. 5e,f). There was a non-significant reduction in the most active form of PC1/3 (66 kDa; 30.0% reduction in BFA-treated islets, *p*=0.072; Fig. 5d) and the mature 50 kDa form of CPE (28.5% reduction in BFA-treated islets, *p*=0.093; Fig. 5g). Furthermore, there was a non-significant increase in proinsulin levels (69.2% increase, *p*=0.118), accompanied by a significant increase in the PI/I ratio in BFA-treated islets (Fig. 5h,j).

### ER stress and chemical inhibition of ER-to-Golgi trafficking alters the expression of prohormone convertase isoforms

Our data show that beta cell-specific SERCA2 deficiency leads to ER Ca^2+^ depletion, which can lead to dysfunction of ER chaperone proteins, impaired protein folding and ER stress. To determine whether SERCA2 deficiency activates ER stress pathways, transcript levels of the unfolded protein response genes *Xbp1*, *Hsp90ab1*, *Dnajc3*, *Bip* (also known as *Hspa5*), *Pdia4* and *Edem2* were determined by qRT-PCR in islets isolated from βS2KO and control mice. The ratio of spliced to unspliced *Xbp1* and mRNA levels of *Dnajc3* and *Edem2* were significantly increased in βS2KO islets. Levels of *Hsp90ab1*, *Bip* and *Pdia4* were increased in islets from βS2KO mice, but the differences did not reach statistical significance (ESM Fig. 3c).

Next, to determine how ER stress and reduced protein trafficking impact prohormone convertase expression patterns, WT INS1 cells were treated with TG (a SERCA inhibitor), TM (a blocker of N-linked glycosylation) or BFA. Both TG and BFA reduced protein levels of the active forms of PC1/3 (66 kDa) and PC2 (68 kDa) in a time-dependent manner, while TM significantly reduced only the active form of PC1/3 after 24 h (ESM Fig. 4a–f). Similarly, siRNA-based SERCA2 knockdown in MIN6 cells decreased protein levels of the active forms of PC1/3 (66 kDa) and PC2 (68 kDa), while knockdown of SERCA2 in INS1 cells decreased protein levels of the active form of PC1/3 (66 kDa) and increased protein levels of proPC2 (70–75 kDa) (ESM Fig. 5a–f). Together, these data suggest that both ER stress and protein trafficking defects alter the maturation of PC1/3 and PC2.

**Fig. 6:**
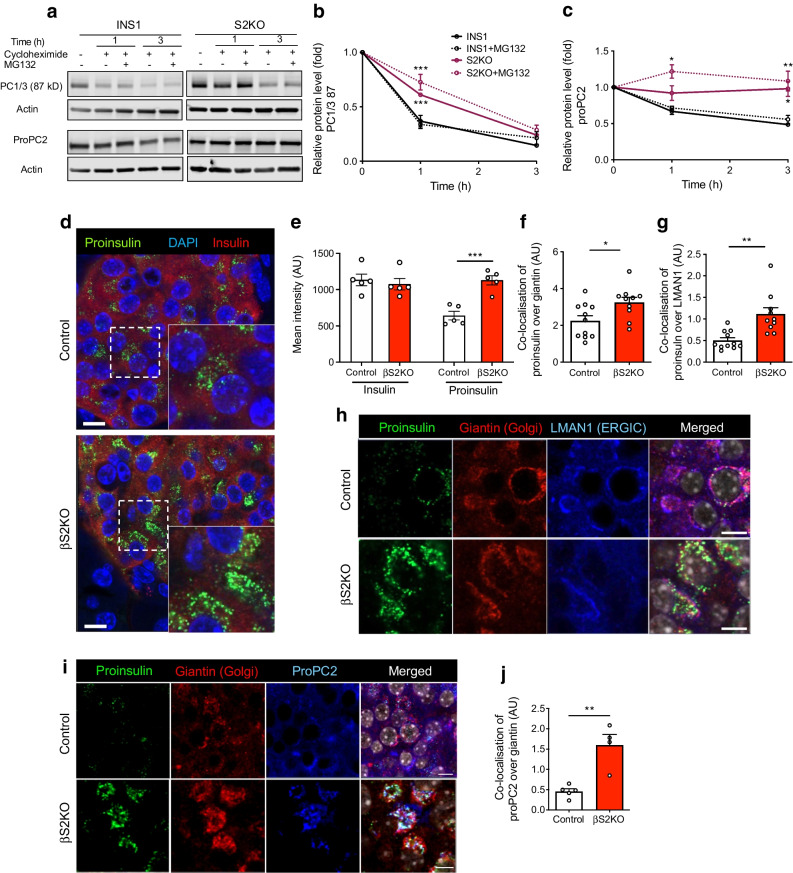
SERCA2 loss disrupts protein trafficking within the beta cell secretory pathway. (**a–c**) WT and S2KO INS-1 cells were treated with 10 mol/l cycloheximide with or without 10 µmol/l MG132 for 9 h prior to protein extraction. (**a**) Representative immunoblots of the 87 kD less active form of PC1/3 and proPC2. (**b**, **c**) Quantification of time-dependent changes in the levels of 87 kD PC1/3 (**b**) and 70–75 kD proPC2 (**c**) after the addition of MG132 (*n*=3) independent experiments. (**d**, **e**) Pancreatic sections from control and βS2KO mice were stained for proinsulin, insulin and DAPI. (**d**) Representative immunofluorescent images and (**e**) quantification of protein expression levels using ImageJ (*n*=5). (**f–h**) Pancreatic sections from control and βS2KO mice were stained for proinsulin, giantin and LMAN1. (**h**) Representative immunofluorescent images and (**f**, **g**) quantification of protein expression levels and localisation using Cell Profiler (*n*=10–11). (**i**, **j**) Pancreatic sections from control and βS2KO mice were stained for proinsulin, giantin and proPC2. (**i**) Representative immunofluorescent images and (**j**) quantification of protein expression levels and localisation using Cell Profiler (*n*=4–5). Results are presented as means ± SEM. Replicate samples are indicated by open circles. *p<0.05, **p<0.01, ***p<0.001 vs indicated groups by two-way ANOVA (**b**, **c**) and Student’s *t* test (**e**–**g** and **j**)

Consistent with the notion that ER Ca^2+^ depletion impairs anterograde trafficking and processing of protein cargo, we observed increased stability of the less active/intermediate form of active PC1/3 (87 kDa) and of proPC2 (70-75 kDa) in S2KO INS1 cells compared with WT cells (Fig. 6a–c). The stability of these two proteins was prolonged further by treatment with the proteasome inhibitor MG132 (Fig. 6a–c).

Next, immunofluorescence staining was performed to investigate changes in protein localisation in islets from βS2KO and control mice. Proinsulin staining intensity was significantly increased in islets from βS2KO mice (Fig. 6d,e). Notably, in islets from βS2KO mice, there was increased co-localisation of proinsulin and LMAN1, a marker of the intermediate region between the ER and the Golgi (i.e. the ERGIC), as well as increased co-localisation of proinsulin and giantin, a marker of the Golgi apparatus (Fig. 6f–h). In addition, co-localisation of proPC2 and giantin was observed in βS2KO mouse islets (Fig. 6i,j). These findings indicate that SERCA2 deficiency leads to an accumulation of proinsulin and proPC2 in the ERGIC and cis-Golgi complex in beta cells, suggesting a defect in trafficking of protein cargo to more distal parts of the secretory pathway.

### Glucolipotoxicity decreases protein expression of active prohormone convertase isoforms in mouse and human islets

A glucolipotoxic environment, similar to that observed in type 2 diabetes, is known to inhibit insulin biogenesis [8, 53], vesicle budding, protein trafficking and ER lipid raft formation [54, 55]. We have previously shown that glucolipotoxic treatment (GLT; 25 mmol/l glucose and 0.5 mmol/l palmitate) reduces beta cell SERCA2 expression and activity in mouse islets and INS-1 cells [26]. To test the relevance of our findings in an in vitro model of type 2 diabetes, we used GLT to evaluate changes in prohormone convertase expression in WT mouse islets and in human islets.

**Fig. 7:**
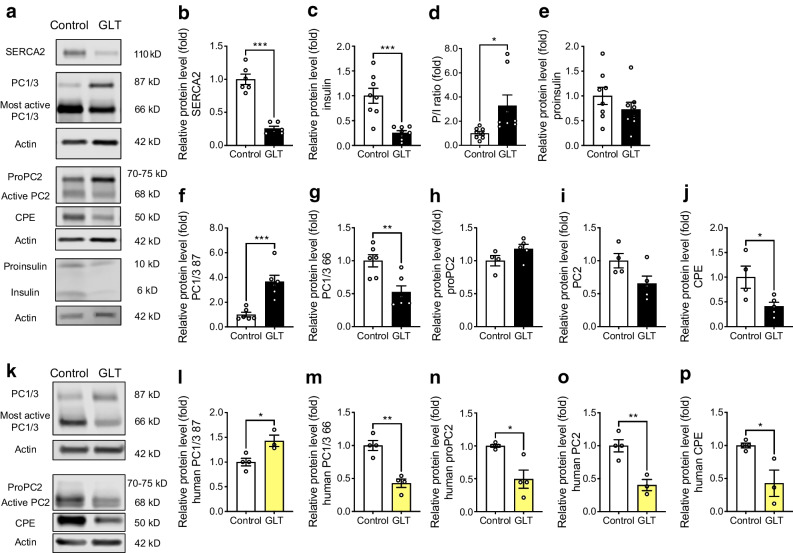
Decreased expression of active prohormone convertase isoforms in GLT-treated mouse and human islets. (**a–j**) Isolated WT mouse islets were subjected to GLT treatment (25 mmol/l glucose + 0.5 mmol/l palmitate) for 24 h. (**a**) Representative immunoblots of SERCA2, PC1/3, PC2, CPE, proinsulin and insulin and (**b–j**) quantification of immunoblotting results: (**b**) SERCA2, (**c**) insulin, (**d**) P/I ratio, (**e**) proinsulin, (**f**) PC1/3 87 kDa, (**g**) PC1/3 66 kDa, (**h**) proPC2, (**i**) PC2, (**j**) CPE. Expression of proteins was normalised to actin expression and FC is relative to the mean control value (*n*=4–8). (**k–p**) Human islets from non-diabetic donors were subjected to GLT treatment for 24 h. (**k**) Representative immunoblots of PC1/3, PC2 and CPE and (**l–p**) quantification of immunoblotting results: (**l**) PC1/3 87 kDa, (**m**) PC1/3 66 kDa, (**n**) proPC2, (**o**) PC2, (**p**) CPE. Expression of proteins was normalised to actin expression and FC is relative to the mean control value (*n*=3–4). Results are presented as means ± SEM. Replicate samples are indicated by open circles. *p<0.05, **p<0.01, ***p<0.001 vs control by Student’s *t* test

In mouse islets treated with GLT, SERCA2 protein expression was reduced by ~80% and the intracellular PI/I ratio was increased (Fig. 7a,b,e). In addition, GLT reduced the levels of the most active form of PC1/3 (66 kDa) and increased levels of the less active/intermediate form (87 kDa) (Fig. 7f,g). Changes in active PC2 levels in response to GLT treatment did not reach statistical significance (Fig. 7h,i), while levels of the active form of CPE were significantly reduced by GLT in mouse islets (Fig. 7j). Finally, a similar immunoblot analysis was performed in islets from human donors without diabetes. Overall, levels of the active forms of PC1/3, PC2 and CPE were all significantly reduced in GLT-treated human islets (Fig. 7k–p).

## Discussion

In this study we developed a mouse model with beta cell-specific deletion of SERCA2 (βS2KO) to test how chronic ER Ca^2+^ depletion impacts beta cell function and insulin production and maturation. Our results show that loss of SERCA2 was sufficient to reduce steady-state beta cell ER Ca^2+^ levels, leading to impaired beta cell glucose-stimulated Ca^2+^ responses and reduced synchronisation between beta cells within islets. This finding was accompanied by a reduction in connexin-36 expression. In addition, βS2KO mice exhibited islet ER stress and mild age-dependent glucose intolerance and had reduced in vivo insulin levels before and after glucose stimulation. Consistent with these findings, dynamic perifusion experiments revealed an insulin secretory defect in islets from βS2KO mice. While this overall phenotype is consistent with our previous study of mice with global SERCA2 haploinsufficiency fed a high-fat diet [26], the beta cell-specific knockout reported here allows us to attribute defects in metabolic function to SERCA2 activity specifically in the beta cell. Moreover, data presented here suggest that SERCA2 deficiency is sufficient to reduce beta cell function with ageing and in the absence of diet-induced obesity. Overall, our results underscore the importance of the ER Ca^2+^ pool in maintaining normal beta cell function, and our findings are consistent with other studies that have documented the importance of SERCA2 and ER Ca^2+^ homeostasis in other disease states, including dermatological disorders [56] and Alzheimer’s disease [57].

Notably, our study provides new insights into the link between SERCA2 deficiency and impaired proinsulin processing. We found that SERCA2 deletion in beta cells led to a prominent defect in proinsulin processing that was reflected in both the pancreas and the serum of βS2KO mice. Alterations in proinsulin processing have been observed in both type 1 and type 2 diabetes [22], and activation of a variety of beta cell stress pathways, including ER, oxidative and lipotoxic stress, have been associated with ER Ca^2+^ depletion and dysfunctional proinsulin processing [58–60]. However, the specific molecular mechanisms underlying defective proinsulin processing have not been well characterised. Previous studies have demonstrated reduced mRNA expression of PC1/3, PC2 and CPE in human islets treated with proinflammatory cytokines and other metabolic stressors [7], but mRNA expression levels of the major processing enzymes were unaffected in βS2KO islets. Similarly, preproinsulin mRNA levels were not reduced. Instead, our data indicate distinct changes in the post-transcriptional processing of both proinsulin and the prohormone processing enzymes. Under normal conditions, PC1/3, PC2 and CPE are synthesised as proenzymes and undergo sequential maturation by autocleavage within different domains of the secretory pathway. This process is closely regulated by both pH and Ca^2+^ (illustrated in Fig. 4i), thus providing spatial regulation of proenzyme activation and proinsulin processing (reviewed in [61]). In islets from βS2KO mice, we observed reductions in the level of the most active form of PC1/3 and accumulation of the proenzyme (inactive) proPC2. In INS-1 cells lacking SERCA2, we showed that these changes were associated with reduced PC1/3 and PC2 enzyme activity, while SERCA2 adenoviral overexpression rescued enzyme activity and proenzyme maturation.

Based on these findings, we suggest a model whereby chronic ER Ca^2+^ depletion and ER stress due to SERCA2 deficiency impairs the spatial regulation of prohormone trafficking, processing and maturation within the beta cell secretory pathway. In addition to immunoblot data showing altered expression patterns of the proinsulin processing enzymes and fluorogenic assays showing reduced activity, several other lines of evidence support this model. First, results from RNA-seq performed using islets from male βS2KO and control mice showed downregulation of genes that were enriched for terms related to vesicle-mediated trafficking and secretory function. Second, we found increased expression of genes associated with ER stress signalling in islets from βS2KO mice. Third, BFA, which inhibits ER-to-Golgi vesicle trafficking, partially reproduced the impairments in PC1/3 and PC2 expression observed in our SERCA2-deficient models and led to an increase in the P/I ratio. Fourth, in S2KO INS-1 cells, we observed an increased half-life of both proPC2 and the less active/intermediate 87 kDa form of PC1/3. Finally, in pancreas sections from βS2KO mice, we found increased co-localisation of proinsulin and proPC2 with markers of the early compartments of the secretory pathway, including the ERGIC and the cis-Golgi.

The mechanisms by which SERCA2 and ER Ca^2+^ regulate hormone maturation and trafficking in the beta cell remain a matter of some speculation. Ca^2+^ is a required cofactor for PC1/3 and PC2 activity (reviewed in [62]). Thus, decreased availability of ER Ca^2+^ could directly affect convertase activity on prohormone substrates, as well as indirectly impact prohormone maturation via reduced intermolecular cleavage of the 87 kDa form of PC1/3 to the more active 66 kDa form [63]. However, independent of reduced convertase activity due to lower availability of Ca^2+^, we suggest that loss of SERCA2 also results in impaired or inefficient anterograde trafficking of both processing enzymes and proinsulin through the various compartments of the secretory pathway, where they undergo sequential maturation, processing and activation.

The importance of ER-to-Golgi trafficking for the development of diabetes has been highlighted recently in a study of neonatal diabetes caused by mutation of *YIPF5* [64], which encodes a protein that cycles between the ER and the Golgi and is predominantly localised in the ER and the ER–Golgi intermediate compartments. Deletion of *YIPF5* caused a 5.5-fold increase in proinsulin staining and a 70% reduction in insulin staining and was associated with a newly described monogenic form of diabetes [64]. At least one previous study has linked lipotoxic stress with impaired ER-to-Golgi trafficking in beta cells [65], and we have shown that SERCA2 expression is reduced in human islets from donors with type 2 diabetes [26]. Here, GLT treatment of human islets partially phenocopied the altered PC1/3 and PC2 expression patterns observed in our SERCA2-deficient mouse model. Finally, a recent study by Ramzy et al demonstrated an unexplained accumulation of PC2 in islets from human organ donors with type 2 diabetes [66]. Our data extend these findings by showing a prolonged protein half-life for the 87 kDa form of PC1/3 and for proPC2 in SERCA2-deficient cells, which we hypothesise may arise from inefficient trafficking through the secretory pathway.

Luminal levels of ER Ca^2+^ as well as Ca^2+^ oscillations have been shown to affect ER-to-Golgi trafficking and coat protein complex II (COPII) vesicle budding, delivery and fusion with the Golgi in other cell types [67]. In keratinocytes, SERCA2 deficiency reduced ER-to-Golgi transport of desmosomal cadherin cargo [68]. Consistent with this observation, in individuals with Darier–White disease, a rare genetic icthyosis disorder resulting from SERCA2 haploinsufficiency, lesion keratinocytes exhibit ER retention of cadherin, a transmembrane protein located at the plasma membrane that typically requires trafficking to the membrane [56]. Only one group has examined PI/I ratios in individuals with Darier–White disease; Ahanian et al described a non-significant trend towards increased ratios in a small cohort of affected individuals compared with matched control participants [69]. Interestingly, this same group found that individuals with Darier–White disease have an increased relative risk of developing type 1 diabetes [70].

There are some limitations in our study that should be acknowledged. Our findings may be relevant to other proteins and hormones that require processing in beta cells and other secretory cell types, such as salivary glands, alpha cells and intestinal cells, which rely heavily on proper protein processing and vesicle trafficking. However, additional experiments are required to understand whether defects in trafficking are generalisable or cell-specific and whether they are cargo-specific. Additionally, during the course of our study, we uncovered a polymorphism in the *P2rx7* gene that impacts many 129 strain-originating transgenic mouse lines [29]. To account for this passenger mutation, all comparisons in this study were made using *Atp2a2*^*fl/fl*^*;Ins1*^*Cre*^-negative mice as controls. Furthermore, it is possible that other ER Ca^2+^ pumps, including SERCA3, may partially compensate for the absence of SERCA2 in our model. However, we note that previous studies have shown that SERCA3 deletion does not result in a glucose-intolerant phenotype [71], suggesting that SERCA3 does not play a major role in beta cell function under normal conditions. Additionally, we did not observe a compensatory increase in SERCA3 mRNA levels (*Atp2a3*) in islets from our βS2KO mice.

Notwithstanding these limitations, our results provide additional insight into the function of SERCA2 and the importance of luminal Ca^2+^ in the regulation of secretory pathway function in pancreatic beta cells. Our results also illustrate a unique relationship between SERCA2 activity and the spatial regulation of proenzyme and prohormone processing in the beta cell.

### Supplementary Information

Below is the link to the electronic supplementary material.

Supplementary file1 (PDF 2.05 MB)

## Data Availability

All data generated or analysed during this study are included in the manuscript and supporting files. Further information and requests for resources and/or reagents should be directed to CE-M (cevansmo@iu.edu). This study did not generate any new unique reagents. RNA-seq data have been deposited in the Gene Expression Omnibus (GEO; accession no.: GSE207498). This paper does not report original code.

## Abbreviations

βS2KO mice: Beta cell-specific SERCA2 knockout mouse
BFA: Brefeldin A
CPE: Carboxypeptidase E
ER: Endoplasmic reticulum
ERGIC: Intermediate region between the ER and the Golgi
FC: Fold change
FRET: Förster resonance energy transfer
Fura-2-AM: Fura-2-acetoxymethylester
GLT: Glucolipotoxic treatment
GO: Gene Ontology
LMAN1: Lectin mannose-binding 1
MIN6: Mouse insulinoma
PC: Prohormone convertase
PI/I: Proinsulin/insulin
qRT-PCR: Quantitative real-time reverse transcription PCR
ROIs: Regions of interest
S2KO: SERCA2-null rat insulinoma cell line
SERCA2: Sarcoendoplasmic reticulum Ca^2+^ ATPase-2
TG: Thapsigargin
TGN: Trans-Golgi network
TM: Tunicamycin
WT: Wild-type

## Authors’ relationships and activities

MCA is currently an employee at Cooke Medical. RNB is currently an employee at Cardinal Health. IL received consulting fees from Weil, Gotshal & Manges. CE-M received grants from Astellas Pharmaceuticals and Lilly Pharmaceuticals and also received consulting fees from Dompe Pharmaceuticals. CE-M also has the following patents: ‘A discovery-based proteomics approach identifies protein disulfide isomerase (PDIA1) as a biomarker of β cell stress in type 1 diabetes’ and ‘Extracellular vesicle ribonucleic acid (RNA) cargo as a biomarker of hyperglycaemia and type 1 diabetes’. CE-M previously participated in or actively participates in the Provention Bio Advisory Board, Isla Technologies Advisory Board, Mai Cell Technologies Advisory Board, Avotres Advisory Board, Neurodon Advisory Board and Innodia Advisory Board. The authors declare that there are no other relationships or activities that might bias, or be perceived to bias, their work.
