# Supplementary material for: SERCA2 regulates proinsulin processing and processing enzyme maturation in pancreatic beta cells
Source: Diabetologia. Author manuscript; Available in PMC 2023 Nov 21. (PMC10542743; doi:10.1007/s00125-023-05979-4)
Supplement: EMS [file NIHMS1931695-supplement-EMS.pdf]

Electronic Supplementary Material

SERCA2 regulates proinsulin processing and processing enzyme maturation in the pancreatic beta cell

Hitoshi Iida, Tatsuyoshi Kono, Chih-Chun Lee, Preethi Krishnan, Matthew C. Arvin, Staci A. Weaver, Timothy S. Jarvela, Renato C. S. Branco, Madeline R. McLaughlin, Robert N. Bone, Xin Tong, Peter Arvan, Iris Lindberg, and Carmella Evans-Molina

Contents:
ESM Table 1. KEY RESOURCES
ESM Table 2. Human Donor Information
ESM Table 3. List of differentially expressed mRNAs (Partial)
ESM Table 4. List of significant pathways identified for differentially expressed mRNAs (Partial)
ESM Table 5. List of significant gene ontology terms identified for differentially expressed mRNAs
ESM Table 6. Checklist for reporting human islet preparations used in research
ESM Fig. 1. SERCA2 deficiency did not impact weight, lean mass, or glucose tolerance in female mice.
ESM Fig. 2. Beta cell-specific SERCA2 deficiency did not alter transcription of prohormone convertase isoforms in islets.
ESM Fig. 3. Markers of ER stress were mildly increased, while insulin gene mRNA levels were not altered in islets isolated from βS2KO mice.
ESM Fig.4. ER stress and inhibition of protein trafficking reduced active prohormone convertases isoforms in beta cell lines.
ESM Fig. 5. siRNA-based SERCA2 knockdown reduced active prohormone convertases isoforms in beta cell lines.

ESM Table 1.
Key Resources

REAGENT or RESOURCE	SOURCE	IDENTIFIER
Antibodies		
Rabbit polyclonal anti-PC1/3	Cell Signaling Technology	Cat# 11914; RRID:AB_2631284)
Rabbit polyclonal PC1/3 C-terminal	Gift from Dr. Iris Lindberg, University of Maryland Vindrola et al., 1992	http://thelindberglab.com/antibodies/
Rabbit monoclonal anti-PC2	Cell Signaling Technology	Cat# 14013; RRID:AB_2631285
Rabbit polyclonal pro-PC2	Gift from Dr. Iris Lindberg, University of Maryland Muller et al., 2000	http://thelindberglab.com/antibodies/
Rabbit polyclonal anti-CPE	Abcam	Cat# ab11044; RRID:AB_297698
Goat polyclonal anti-SERCA2	Santa Cruz Biotechnology	Cat# sc-8095; RRID:AB_2290108
Mouse monoclonal anti-SERCA2	Santa Cruz Biotechnology	Cat# sc-376235; RRID:AB_10989947
Mouse monoclonal anti-Actin	Millipore	Cat# MAB1501; RRID:AB_2223041
Mouse monoclonal anti-beta-Actin	Santa Cruz Biotechnology	Cat# sc-47778; RRID:AB_626632
Guinea pig polyclonal anti-Insulin	Agilent Dako	Cat# A0564; RRID:AB_10013624
Mouse monoclonal anti-proInsulin	Developmental Studies Hybridoma Bank	Cat# GS-9A8-C
Rabbit monoclonal anti-Insulin (C27C9)	Cell Signaling Technology	Cat# 3014, RRID:AB_2126503
Rabbit monoclonal anti-LMAN1 (ERGIC53)	Abcam	Cat# ab125006; RRID:AB_10973984
Rabbit polyclonal anti-Connexin36 (GJD2)	Novus Biological	Cat# NBP1-59254; RRID:AB_11039152
Goat polyclonal Anti-Giantin	Santa Cruz Biotechnology	Cat# sc-46993; RRID:AB_2279271
IIRDye® 800CW Donkey anti-Mouse IgG Secondary Antibody	LI-COR Bioscience	Cat# 926-32212, RRID:AB_621847
IRDye® 800CW Donkey anti-Goat IgG Secondary Antibody	LI-COR Bioscience	Cat# 926-32214, RRID:AB_621846
IRDye® 680RD Donkey anti-Rabbit IgG Secondary Antibody	LI-COR Bioscience	Cat# 926-68073, RRID:AB_10954442
Donkey anti-guinea pig IgG Secondary Antibody (Alexa Fluor-647)	Jackson ImmunoResearch Laboratories, Inc	Cat# 706-605-148, RRID: AB_2340476
Donkey anti-goat IgG Secondary Antibody (Alexa Fluor-568)	Thermo Fisher Scientific	Cat# A11057, RRID: AB_ 2534104
Donkey anti-rabbit IgG Secondary Antibody (Alexa Fluor-647)	Thermo Fisher Scientific	Cat# A31573, RRID: AB_2536183

Donkey anti-mouse IgG Secondary Antibody (Alexa Fluor-488)	Thermo Fisher Scientific	Cat# A-21202, RRID: AB_141607
Mouse-on-Mouse IgG Blocking Solution	Vector Laboratories	Cat# MKB-2213-1
Vector® NovaRED™ Substrate Kit, Peroxidase	Vector Laboratories	Cat# SK-4800
Bacterial and virus strains		
adenovirus expressing human SERCA2b	Gift from Dr. Umut Ozcan, Harvard Medical School	N/A
adenovirus expressing RIP-D4ER Cameleon probe	Gift from Dr. Richard Benninger, University of Colorado<br>Greotti et al., 2016	N/A
Adenovirus expressing RIP-GCaMP6s	VectorBuilder	N/A
Biological samples		
Human donor islets	See Supplemental Table S2	N/A
Chemicals, peptides, and recombinant proteins		
Insulin (regular, short-acting)	Novo Nordisk	NC0769896
Humulin R	Eli Lilly	NDC 0002-8215-01
D-Glucose	Sigma Aldrich	Cat# G7528
Brefeldin A (BFA)	Sigma Aldrich	Cat# B6542
Thapsigargin (TG)	Cayman Chemical	Cat# 10522
Tunicamycin (TM)	Cayman Chemical	Cat# 11445
Palmitate	Sigma Aldrich	Cat# P0500
Bovine aprotinin	Cayman Chemical	Cat# 14716
Cyclohexamide	Sigma Aldrich	Cat# C4859
MG132	Sigma Aldrich	Cat# M7449
RPMI1640	Gibco	Cat# 11875-093
DMEM	Gibco	Cat# 11965-092
Opti Mem I	Gibco	Cat# 31985-070
pERTKR-aminomethylcumarin	Peptide International	Cat# MPR-3159-v
Aminomethylcumarin	Sigma Aldrich	Cat# A9891
N-p-tosyl-L-phenylalanine chloromethyl ketone	Sigma Aldrich	Cat# T4376
trans-epoxysuccinyl-L-leucylamido(4-guanidino) butane	Sigma Aldrich	Cat# 66701
Nα-tosyl-L-lysine chloromethyl ketone	Sigma Aldrich	Cat# T7254
7B2-CT peptide	Gift from Dr. Iris Lindberg, University of Maryland	N/A

ProSAAS-CT peptide	Gift from Dr. Iris Lindberg, University of Maryland	N/A
Lipofectamin RNAiMAX	Thermo Fisher Scientific	Cat# 13778-075
Moloney murine leukaemia virus reverse transcriptase	Thermo Fisher Scientific	Cat# 28025013
DC protein assay kit II	Bio-Rad	Cat# 5000112
4-20% Mini-Protean TGX gel	Bio-Rad	Cat# 4561096
Odyssey blocking buffer	LI-COR Biosciences	Cat# 927-40000
Signal Enhancer HIKARI 250	nacalai tesque	Cat# 2994
Bovine Aprotinin	Cayman Chemical	Cat# 14716
Animal-Free Blocker and Diluent, R.T.U.	Vector Laboratories	Cat# SP-5035-100
FluorSave Reagent	Merck Millipore	Cat# 345789
cOmplete protease inhibitor cocktail, EDTA-free	Roche	Cat# 04693132001
PhosSTOP	Roche	Cat# 4906845001
TRIS	Sigma Aldrich	Cat# 11814273001
Deoxycholate	Sigma Aldrich	Cat# 6750
IGEPAL CA-630	Sigma Aldrich	Cat# I8896
SDS	Sigma Aldrich	Cat# L3771
Sarcosyl,	Sigma Aldrich	Cat# L7414
Glycerol	Sigma Aldrich	Cat# G5516
Dithiothreitol	Roche	Cat# 10197777001
EDTA	Sigma Aldrich	Cat# E9884
Sodium flouride	Sigma Aldrich	Cat# 201154
$MgCl_2$	Sigma Aldrich	Cat# M8266
Benzonase nuclease	Millipore	Cat# 70746-3CN
Critical commercial assays		
RNeasy Mini Kit	Qiagen	Cat# 74136
RNeasy Micro Kit	Qiagen	Cat# 74034
DNeasy Blood and Tissue kit	Qiagen	Cat# 69504
SensiFASTÔSYBR Lo-ROX kit	Bioline	Cat# BIO-94020
BCA protein determination	BioRad	Cat# 500-0112
Mouse Insulin ELISA	Mercodia	Cat# 10-1247-10
Fura-2 acetoxymethylester	Invitrogen	Cat# F1221
Mouse ProInsulin ELISA	Alpco Diagnostics	Cat# 80-PINMS-E01
KAPA mRNA Hyper Prep Kit	Roche	Cat# KK8540
Experimental models: Cell lines		
Rat (male) SERCA2 null INS-1 832/13	This paper	N/A
Rat (male) INS-1 832/13	H E Hohmeier et al., 2000	N/A

Mouse (sex unspecified) MIN6	J Miyazaki et al, 1990	N/A
Experimental models: Organisms/strains		
Mouse: β cell specific SERCA2-null (βS2KO) in C57BL6/J background	This paper	N/A
Mouse: C57BL6/J	The Jackson Laboratory	JAX # 000664
Teklad 2018SX	Inotivco	N/A
Oligonucleotides		
Mouse PC1/3-F	This paper	AGTTGGAGGCATAAGAATGCTG
Mouse PC1/3-R	This paper	GCCTTCTGGGCTAGTCTGC
Mouse PC2-F	This paper	AGAGAGACCCCAGGATAAAGATG
Mouse PC2-R	This paper	CTTGCCCAGTGTTGAACAGGT
Mouse CPE-F	This paper	GCTCAGGTAATTGAAGTCTT
Mouse CPE-R	This paper	TACTGCTCACGAATACAGTT
Mouse SERCA2b-F	This paper	GATCCTCTACGTGGAACCTTTG
Mouse SERCA2b-R	This paper	CCACAGGGAGCAGGAAGAT
Mouse SERCA3-F	This paper	AGGGGAAGCTAAGAAGCCAG
Mouse SERCA3-R	This paper	CCCTCAGACTCCTCCTACCC
Mouse beta-actin-F	This paper	AGGTCATCACTATTGGCAACGA
Mouse beta-actin-R	This paper	CACTTCATGATGGATTGAATGTAGTT
Mouse spriced-XBP1-F	This paper	CTGAGTCCGAATCAGGTGCAG
Mouse spriced-XBP1-R	This paper	GTCCATGGGAAGATGTTCTGG
Mouse unspriced-XBP1-F	This paper	TGGCCGGGTCTGCTGAGTCCG
Mouse unspriced-XBP1-R	This paper	GTCCATGGGAAGATGTTCTGG
Mouse HSP90ab1-F	This paper	TGTATGTACGCCGCGTATTCA
Mouse HSP90ab1-R	This paper	TCGGAATCCACAACACCTTTG
Mouse Dnajc3-F	This paper	GACAGCTAGCCGACGCCTTA
Mouse Dnajc3-R	This paper	GTCACCATCAACTGCAGCGT
Mouse Bip-F	This paper	TTCAGCCAATTATCAGCAAACTCT
Mouse Bip-R	This paper	TTTTCTGATGTATCCTCTTCACCAGT
Mouse Pdia4-F	This paper	TGACCCGGCCTACTTGCA
Mouse Pdia4-R	This paper	GTGTGGTGAAACTTGTAATCTTCTCTCA
Mouse Edem2-F	This paper	ATGCCTTTCCGGCTACTCATC
Mouse Edem2-R	This paper	CCTTGACTCGCTCCCTGTAGT
Atp2a2 Mouse siRNA Oligo Duplex	OriGene	CAT#: SR421850 GCAGUUUGAAGACUUACUAGUUAGA
Software and algorithms		
Zen Blue edition ver2.3	Carl Zeiss	https://www.zeiss.com/microscopy/int/products/microscope-software.html; RRID:SCR_013672
Axio-Vision Software	Carl Zeiss	https://www.zeiss.com/microscopy/int/products/microscope-software.html; RRID:SCR_002677
ImageJ ver1.52p	Fiji; Schneider et al., 2012	Open source: https://imagej.net/Fiji; RRID:SCR_002285
Image Studio	LI-COR	https://www.licor.com/bio/image-studio-lite/download; RRID:SCR_013715

FastQC 0.11.5.	Babraham Bioinformatics	https://www.bioinformatics.babraham.ac.uk/projects/download.html RRID:SCR_011106
Prism 7.0	GraphPad Software	https://www.graphpad.com/; RRID:SCR_002798
Flow software version 10.0.20.1231	Partek	https://www.partek.com/partek-flow/; RRID:SCR_011860
STAR aligner ver. 2.7.3a	Dobin A et al., 2013	https://github.com/alexdobin/STAR/releases; RRID:SCR_004463
RefSeq release 93	NCBI	https://www.ncbi.nlm.nih.gov/refseq/; RRID:SCR_003496
DESeq2	Bioconductor	Open source: https://bioconductor.org/packages/release/bioc/html/DESeq2.html; RRID:SCR_015687
Ingenuity Pathway Analysis	Qiagen	https://digitalinsights.qiagen.com/product-login/; RRID:SCR_008653
Metascape	Metascape	https://metascape.org; RRID:SCR_016620
ggplot2	MIT	https://ggplot2.tidyverse.org; RRID:SCR_014601
CellProfiler 4.1.3	Cellprofiler.org	https://cellprofiler.org/previous-releases RRID:SCR_007358
Qubit Fluorometer	Thermo Fisher Scientific	RRID:SCR_020553
Perifusion System	Biorep Technologies, Inc	N/A
LSM 800 confocal imaging system	Carl Zeiss	RRID:SCR_015963
LSM-700 confocal microscope	Carl Zeiss	RRID:SCR_017377
Bioanalyzer 2100	Agilent	Cat# G2939BA; RRID:SCR_019715
HiSeq 4000 sequencer	Illumina	RRID:SCR_016386
Odyssey CLx scanner	LI-COR	RRID:SCR_014579
EchoMRI-500	EchoMRI	RRID:SCR_017104
Contour	Bayer	N/A
QuantStudio 3 thermal cycle	Applied Biosystems	RRID:SCR_018712
Zeiss Lightsheet Z.1 Lightsheet Fluorescence Microscope	Carl Zeiss	RRID:SCR_020919
Zeiss LSM 880 with Airyscan Confocal Laser Scanning Microscope	Carl Zeiss	RRID:SCR_020925
SpectraMax M5 plate reader	Molecular Devices	RRID:SCR_020300
SpectraMax iD5 plate reader	Molecular Devices	N/A
Mini-Protean Tetra apparatus	Bio-Rad	Cat# 1658005JA
Odyssey 1828 Scanner	LI-COR Biosciences	RRID:SCR_014579

ESM Table 2.
Human Donor Information

Arrival Date	Unique Identifier	Donor UNOS ID	Gender	Ethnicity/Race	Age	BMI	Diabetic donor status	Islett purity	Islet viability
2018.1.10	AEL5345	AEL5345	M	Hispanic/Latino	48	32.4	No	95	95
2018.2.5	AFA3256	AEDZ307	M	White	45	29.8	No	95	95
2017.10.	RRID:SAMN08768997	AEIY348	M	Native Hawaiian	24	38.1	No	50	98
2017.9	AEIL428A	AEIL428A	M	White	27	30	No	95	99

ESM Table 3.
List of differentially expressed mRNAs (Partial)

Supplementary Table S3: List of differentially expressed mRNAs											
mRNA	Linear scale fold change	pvalue	mRNA	Linear scale fold change	pvalue	mRNA	Linear scale fold change	pvalue	mRNA	Linear scale fold change	pvalue
Cd22	-22.12	2.37E-05	Msr1	-4.48	1.55E-03	Nlrc5	-1.69	6.83E-04	Tgfbi	-1.54	6.18E-03
Pax5	-22.05	3.09E-03	Klrk1	-4.44	4.32E-02	Amot	-1.69	1.05E-02	Ifitm3	-1.54	7.47E-03
Timd4	-19.74	2.94E-03	Sp110	-4.38	3.14E-02	Cnn2	-1.69	2.17E-02	Gpat3	-1.53	3.41E-02
H2-Ob	-11.87	1.22E-02	C3	-4.38	9.35E-03	Eps8l1	-1.69	2.68E-03	Tgfbr2	-1.53	1.50E-02
Fcmr	-11.64	2.03E-03	Plac8	-4.36	2.97E-02	Usp43	-1.68	3.33E-02	Acot7	-1.53	1.45E-02
Fcrl1	-11.46	5.97E-03	Slc9a4	-4.33	3.58E-04	S100a16	-1.68	2.38E-02	Tinagl1	-1.53	2.75E-02
Cd37	-11.16	9.42E-04	Xlr4a	-4.23	9.68E-05	Inpp5d	-1.67	4.68E-02	Avpi1	-1.53	1.22E-02
Slpi	-11.06	2.00E-05	Cd52	-4.23	5.99E-03	Lilr4b	-1.67	1.94E-03	Adgre5	-1.52	1.39E-02
Xlr4b	-10.84	3.11E-08	Gja5	-4.20	1.73E-02	Card10	-1.66	3.37E-02	Bgn	-1.51	3.65E-03
Cr2	-10.45	1.05E-03	Gsta3	-4.17	2.13E-06	Btc	-1.66	3.03E-02	Tnfaip2	-1.51	9.00E-03
Slc26a9	-9.87	1.04E-04	Slc39a4	-4.14	5.83E-04	Lilrb4a	-1.66	6.00E-03	Pkp3	-1.51	3.47E-02
Pou2af1	-9.77	3.78E-02	Cxcl5	-4.10	1.12E-02	Serpinf1	-1.65	4.19E-02	Sh3bgrl3	-1.51	6.88E-03
Ly6d	-9.52	5.36E-05	Themis2	-4.05	3.20E-03	Hyal1	-1.65	2.76E-02	Fxyd3	-1.51	2.72E-02
Wfdc2	-9.47	9.01E-05	Lair1	-4.03	6.17E-03	Tuba1c	-1.65	1.74E-03	Arhgap28	-1.51	4.58E-02
Cxcr5	-9.21	5.95E-04	Slc9a2	-4.02	3.50E-04	Plau	-1.64	4.99E-03	Myof	-1.51	5.23E-03
Ifi214	-8.99	2.93E-03	Pglyrp1	-4.01	3.18E-03	Ano1	-1.64	2.01E-02	Cadm3	-1.50	7.18E-03
Folr2	-8.81	1.06E-02	Ccl9	-4.00	5.09E-04	Shisa4	-1.64	2.89E-02	Rit2	-1.50	2.75E-02
Tacstd2	-8.57	5.09E-03	Klk1b3	-4.00	2.40E-02	Blnk	-1.64	4.97E-02	Tnfrsf1b	-1.50	2.19E-02
Clec4n	-8.47	1.04E-02	Adgrb2	-3.99	7.78E-03	C3ar1	-1.63	1.34E-02	Cdk6	-1.50	1.61E-02
Gm2663	-8.42	2.64E-03	Kctd14	-3.93	6.43E-03	Epha1	-1.63	2.13E-02	Cfap61	1.53	3.20E-02
Cd79b	-8.15	2.00E-02	Gm5771	-3.91	2.94E-02	F3	-1.63	6.50E-03	Dapl1	1.53	2.72E-02
Anxa8	-8.01	2.96E-05	C1qtnf5	-3.85	3.30E-02	Nes	-1.63	2.82E-04	Arhgap36	1.54	1.60E-02
Spink4	-8.00	2.43E-04	Vnn3	-3.81	1.54E-02	Mpeg1	-1.63	1.11E-02	Rasl10b	1.54	6.64E-03
Duox2	-7.94	4.63E-03	Trim30b	-3.80	3.04E-02	Onecut2	-1.62	5.45E-03	Frmd5	1.55	1.09E-03
Ltf	-7.93	2.50E-02	Mpzl2	-3.79	1.12E-04	Ctss	-1.62	2.95E-02	Gm14288	1.58	5.04E-03
Wnt7a	-7.60	4.20E-04	Phf11b	-3.78	1.59E-02	Itga2	-1.61	9.34E-04	Cpne4	1.58	4.50E-02
Serpinb5	-7.53	2.13E-03	Il33	-3.77	3.48E-02	Itgb6	-1.60	7.20E-03	Dach2	1.59	2.15E-02
Wdfy4	-7.43	1.63E-03	Tmc5	-3.77	1.35E-04	Myc	-1.60	9.57E-03	Trpc4	1.62	1.98E-03
Blk	-7.22	1.56E-03	Arhgap15	-3.72	4.55E-02	Cldn2	-1.60	3.94E-02	AI182371	1.63	2.36E-02
Ugt1a1	-7.22	2.43E-05	Arhgap45	-3.69	1.76E-02	Smad3	-1.60	3.86E-03	Gm37240	1.83	4.77E-02
H2-Oa	-7.08	2.69E-02	Reg3g	-3.67	5.96E-04	Il18r1	-1.59	2.21E-02	Adam32	1.90	3.86E-02
Mmp7	-6.96	1.64E-02	Cd79a	-3.65	6.53E-04	Mlph	-1.59	7.34E-05	Kcnh5	1.98	2.25E-02
Gabrp	-6.92	2.12E-03	Ly86	-3.64	1.83E-02	Gvin1	-1.59	9.25E-03	Rbm20	2.06	1.52E-06
Napsa	-6.43	5.64E-03	Klk1b4	-3.62	1.82E-02	Gdnf	-1.59	2.15E-02	Vmn2r29	2.16	4.20E-02
Fgr	-6.17	1.22E-02	Atp2a2	-3.61	8.75E-71	Olfml2a	-1.59	2.70E-02	Il12b	2.32	1.73E-02
Slc10a2	-5.96	1.66E-04	Klhdc7a	-3.56	3.57E-02	Ltbp1	-1.59	1.57E-02	Gm3500	2.36	2.90E-02
Duoxa2	-5.94	7.66E-05	Cmah	-3.54	2.94E-02	Col18a1	-1.58	8.74E-03	Pmfbp1	3.15	3.40E-02
Traf3ip3	-5.81	3.27E-02	Epn3	-3.54	5.33E-04	St3gal4	-1.58	3.74E-02	Gm20517	3.94	7.71E-03
Fcna	-5.53	1.15E-02	Egr4	-3.53	3.89E-02	Prph	-1.58	3.70E-03	Apob	4.61	1.76E-02
Cnr2	-5.50	2.85E-02	Adgrg7	-3.53	1.52E-02	Itga5	-1.58	5.68E-03	Apol7c	5.09	3.11E-02
Lcn2	-5.35	9.06E-04	Lamb3	-3.46	4.43E-04	Igfbp4	-1.58	6.51E-03	Clca1	8.95	9.36E-03
Trim30c	-5.27	3.13E-02	Cd300ld3	-3.45	1.93E-02	Apoe	-1.58	2.73E-03			
Reg3b	-5.26	1.62E-03	Dsg3	-3.41	1.39E-02	Metrnl	-1.57	3.01E-02			
ccdc198	-5.23	1.99E-02	Krt23	-3.40	1.56E-08	Htr3a	-1.57	6.86E-03			
Itgam	-5.19	3.91E-06	Nos2	-3.40	1.72E-02	Fxyd5	-1.56	2.98E-02			
Sema3a	-5.07	3.45E-02	Tmem171	-3.38	1.40E-02	Procr	-1.56	6.59E-04			
Ctse	-5.05	2.18E-04	Cdh20	-3.38	1.95E-02	Rest	-1.56	2.13E-02			
Hamp2	-5.03	6.36E-03	Trh	-3.38	4.29E-03	Kcnq5	-1.56	2.30E-02			
Cfb	-4.97	3.90E-02	Tns4	-3.37	4.24E-02	Itpripl2	-1.56	3.33E-03			
Reg2	-4.85	1.39E-03	Bglap3	-3.36	2.54E-02	Ngf	-1.56	3.26E-02			
Gjb4	-4.85	2.52E-03	C4b	-3.35	1.27E-05	Vim	-1.55	9.19E-03			
Tdh	-4.82	2.91E-02	Itgb2	-3.35	4.89E-04	Pdgfb	-1.55	9.54E-04			
Car9	-4.74	2.88E-02	Raet1e	-3.35	2.45E-02	Cd9	-1.55	1.00E-03			
Sema3d	-4.67	1.21E-02	Pzp	-3.34	3.01E-02	Avil	-1.55	2.74E-03			
Mcoln2	-4.66	2.81E-02	S100a14	-3.33	1.14E-03	Col12a1	-1.54	8.55E-03			
Wnt7b	-4.66	3.05E-05	Ugt1a7c	-3.33	3.38E-05	Plpp2	-1.54	1.70E-02			
Gsta4	-4.59	3.33E-06	Myo5c	-3.32	3.54E-02	Gpm6b	-1.54	1.48E-02			
Lrmp	-4.52	3.43E-03	Rac2	-3.30	2.22E-02	Epha2	-1.54	2.76E-02			

RNA isolated at 17-weeks of age from control and βS2KO islets was subjected to bulk RNA sequencing analysis. Sequencing files were analyzed using the Flow software. The raw sequencing files were aligned to the mouse genome (mm10) using STAR aligner. Uniquely mapped reads were annotated using RefSeq. mRNAs with a total of at least 10 read counts were retained for further analysis. mRNAs with linear scale fold-change ≥ 1.5 and $p < 0.05$ were identified using DESeq2 and were considered as differentially expressed mRNAs. Biological pathways were identified using QIAGEN Ingenuity Pathway Analysis, and gene ontology enrichment was performed using Metascape. Functional terms with $p < 0.05$ were considered significant.

ESM Table 4.

List of significant pathways identified for differentially expressed mRNAs (Partial)

Supplementary table S4: List of significant pathways identified for differentially expressed mRNAs

Ingenuity Canonical Pathways	p-value	Gene Ratio	Molecules
Phagosome Formation	6.31E-12	0.143	C3AR1,CR2,FCER2,FCGR2A,FCGR2B,ITGA2,ITGA5,ITGAM,ITGB2,ITGB4,MSR1,PIK3CD,RAC2,SCARA3,SYK,TLR4,TLR7,TLR9,VTN
TREM1 Signaling	1.29E-08	0.160	CIITA,CXCL3,FCGR2B,IL1B,ITGA5,LAT2,Naip1 (includes others),NLRC4,NLRC5,TLR4,TLR7,TLR9
STAT3 Pathway	8.32E-06	0.089	CSF2RB,FGFR2,FGFR3,IGF1,IL10RA,IL18R1,IL1A,IL1B,MYC,NGFR,PDGFB,TGFBR2
MSP-RON Signaling Pathway	1.12E-05	0.138	CSF2RB,IL12B,ITGAM,ITGB2,KLK3,NOS2,PIK3CD,TLR4
Macropinocytosis Signaling	1.12E-05	0.118	CSF1R,ITGA5,ITGB2,ITGB4,ITGB6,NGF,PDGFB,PDGFC,PIK3CD
PTEN Signaling	4.79E-05	0.081	FGFR2,FGFR3,INPP5D,ITGA2,ITGA5,ITGB2,ITGB4,NGFR,PIK3CD,RAC2,TGFBR2
Caveolar-mediated Endocytosis Signaling	6.17E-05	0.110	CD55,FLNC,ITGA2,ITGA5,ITGAM,ITGB2,ITGB4,ITGB6
GP6 Signaling Pathway	5.50E-04	0.072	APBB1IP,BTK,COL12A1,COL18A1,COL6A5,LAMB3,PIK3CD,SYK,VAV1
Antigen Presentation Pathway	7.41E-04	0.128	CIITA,HLA-DMB,HLA-DOA,HLA-DOB,NLRC5
Type I Diabetes Mellitus Signaling	1.10E-03	0.072	HLA-DMB,HLA-DOA,HLA-DOB,IL12B,IL1B,NGFR,NOS2,TNFRSF1B
Phagosome Maturation	7.24E-03	0.053	CTSC,CTSE,CTSH,CTSS,CYBB,RAB7B,TUBA1C,TUBB2B
HIPPO signaling	2.09E-02	0.059	AMOT,SFN,SMAD3,TEAD2,WWC1
Glutathione-mediated Detoxification	2.09E-02	0.094	GSTA3,Gsta4,GSTO1
Autophagy	2.63E-02	0.066	CTSC,CTSE,CTSH,CTSS
Calcium-induced T Lymphocyte Apoptosis	3.39E-02	0.061	ATP2A2,HLA-DMB,HLA-DOA,HLA-DOB
Clathrin-mediated Endocytosis Signaling	1.00E-03	0.057	APOB,APOE,IGF1,ITGA5,ITGB2,ITGB4,ITGB6,PDGFB,PDGFC,PIK3CD,TF
NF-κB Signaling	1.35E-06	0.084	CARD10,FGFR2,FGFR3,IL1A,IL1B,IL1RN,IL33,NGF,NGFR,PIK3CD,TGFBR2,TLR4,TLR7,TLR9,TNFRSF1B
Glucocorticoid Receptor Signaling	2.00E-05	0.050	ANXA1,BGLAP,CEBPA,CSF2RB,CXCL3,IGF1,IL10RA,IL18R1,IL1A,IL1B,IL1RN,KRT17,KRT19,KRT23,KRT80,MYC,NOS2,PIK3CD,PLAU,PTGS2,SLPI,SMAD3,TGFBR2
Role of NFAT in Regulation of the Immune Response	5.89E-04	0.061	BLNK,BTK,CD79A,CD79B,FCGR2A,FCGR2B,HLA-DMB,HLA-DOA,HLA-DOB,PIK3CD,SYK
Sphingosine-1-phosphate Signaling	2.19E-02	0.051	CASP4,PDGFB,PDGFC,PIK3CD,RAC2,S1PR2
LXR/RXR Activation	1.00E-10	0.140	APOB,APOE,C3,C4A/C4B,IL1A,IL1B,IL1RN,IL33,MSR1,NGFR,NOS2,PTGS2,SERPINF1,TF,TLR4,TNFRSF1B,VTN
Granulocyte Adhesion and Diapedesis	6.03E-10	0.110	Ccl6,Ccl7,Ccl9,CLDN2,CLDN8,CSF3,CXCL3,CXCL6,IL1A,IL1B,IL1RN,IL33,ITGAM,ITGB2,MMP19,MMP7,NGFR,SDC1,TNFRSF1B
Hepatic Fibrosis / Hepatic Stellate Cell Activation	1.29E-08	0.097	COL12A1,COL18A1,COL6A5,CXCL3,EDN1,FGFR2,IGF1,IGFBP4,IL10RA,IL1A,IL1B,NGFR,PDGFB,PDGFC,SMAD3,TGFBR2,TLR4,TNFRSF1B
Complement System	1.86E-08	0.243	C1QC,C3,C3AR1,C4A/C4B,CD55,CFB,CR2,ITGAM,ITGB2
Communication between Innate and Adaptive Immune Cells	2.14E-07	0.125	Ccl9,CD79A,CD79B,IL12B,IL1A,IL1B,IL1RN,IL33,TLR4,TLR7,TLR9,TNFSF13
PI3K Signaling in B Lymphocytes	3.02E-07	0.101	BLNK,BTK,C3,CARD10,CD79A,CD79B,CR2,FCGR2B,INPP5D,PIK3AP1,PIK3CD,SYK,TLR4,VAV1
Dendritic Cell Maturation	3.63E-07	0.087	COL18A1,FCGR2A,FCGR2B,HLA-DMB,HLA-DOA,HLA-DOB,IL12B,IL1A,IL1B,IL1RN,IL33,NGFR,PIK3CD,TLR4,TLR9,TNFRSF1B
B Cell Receptor Signaling	4.17E-07	0.086	APBB1IP,BLNK,BTK,CARD10,CD22,CD79A,CD79B,FCGR2A,FCGR2B,INPP5D,PAX5,PIK3AP1,PIK3CD,RAC2,SYK,VAV1
FXR/RXR Activation	6.46E-07	0.103	APOB,APOE,C3,C4A/C4B,IL1A,IL1B,IL1RN,IL33,SDC1,SERPINF1,SLC10A2,TF,VTN
Agranulocyte Adhesion and Diapedesis	6.92E-07	0.083	Ccl6,Ccl7,Ccl9,CLDN2,CLDN8,CXCL3,CXCL6,IL1A,IL1B,IL1RN,IL33,ITGA2,ITGA5,ITGB2,MMP19,MMP7
T Helper Cell Differentiation	9.55E-07	0.137	CXCR5,HLA-DMB,HLA-DOA,HLA-DOB,IL10RA,IL12B,IL18R1,NGFR,TGFBR2,TNFRSF1B
MSP-RON Signaling In Macrophages Pathway	1.29E-06	0.106	CIITA,HLA-DMB,HLA-DOA,HLA-DOB,IL12B,ITGAM,ITGB2,KLK3,NOS2,PIK3CD,PTGS2,TLR4
Th1 and Th2 Activation Pathway	3.98E-06	0.082	HLA-DMB,HLA-DOA,HLA-DOB,IL10RA,IL12B,IL18R1,IL33,ITGB2,NOTCH2,PIK3CD,SPI1,TGFBR2,TIMD4,VAV1
Th2 Pathway	9.12E-06	0.088	HLA-DMB,HLA-DOA,HLA-DOB,IL12B,IL33,ITGB2,NOTCH2,PIK3CD,SPI1,TGFBR2,TIMD4,VAV1
IL-17 Signaling	4.90E-05	0.070	CSF3,CXCL3,IL12B,IL1A,IL1B,IL33,LCN2,LIF,NOS2,PDGFC,PIK3CD,PTGS2,TNFSF13
Differential Regulation of Cytokine Production in Intestinal Epithelial Cells by IL-17A and IL-17F	5.50E-05	0.217	CSF3,IL12B,IL1A,IL1B,LCN2
FcγRIIB Signaling in B Lymphocytes	7.41E-05	0.107	BLNK,BTK,CD79A,CD79B,FCGR2B,INPP5D,PIK3CD,SYK
Toll-like Receptor Signaling	8.13E-05	0.105	IL12B,IL1A,IL1B,IL1RN,IL33,TLR4,TLR7,TLR9
Th1 Pathway	8.71E-05	0.083	HLA-DMB,HLA-DOA,HLA-DOB,IL10RA,IL12B,IL18R1,ITGB2,NOTCH2,PIK3CD,VAV1
Virus Entry via Endocytic Pathways	1.20E-04	0.088	CD55,FLNC,ITGA2,ITGA5,ITGB2,ITGB4,ITGB6,PIK3CD,RAC2
Acute Phase Response Signaling	1.41E-04	0.067	C3,C4A/C4B,CFB,IL1A,IL1B,IL1RN,IL33,NGFR,PIK3CD,SERPINF1,TF,TNFRSF1B
Neuregulin Signaling	1.48E-04	0.086	AREG,BTC,DCN,ERBB2,ITGA2,ITGA5,ITGB2,ITGB4,MYC
PPAR Signaling	1.48E-04	0.086	IL1A,IL1B,IL1RN,IL33,NGFR,PDGFB,PDGFC,PTGS2,TNFRSF1B
Regulation Of The Epithelial Mesenchymal Transition By Growth Factors Pathway	2.14E-04	0.064	ERBB2,FGFR2,FGFR3,NGFR,PDGFB,PDGFC,PIK3CD,SMAD3,TGFBR2,TNFRSF1B,TNFSF13,VIM
HMGB1 Signaling	2.69E-04	0.067	IL12B,IL1A,IL1B,IL33,LIF,NGFR,PIK3CD,RAC2,TLR4,TNFRSF1B,TNFSF13
IL-10 Signaling	3.16E-04	0.100	FCGR2A,FCGR2B,IL10RA,IL1A,IL1B,IL1RN,IL33
LPS/IL-1 Mediated Inhibition of RXR Function	3.16E-04	0.058	ABCC3,APOE,FABP5,GSTA3,GSTO1,HS3ST1,IL1A,IL1B,IL1RN,IL33,NGFR,TLR4,TNFRSF1B
IL-15 Production	4.27E-04	0.074	BTK,CSF1R,EPHA1,EPHA2,ERBB2,FGFR2,FGFR3,FGR,SYK
HIF1α Signaling	4.68E-04	0.059	CYBB,EDN1,HK2,IGF1,MMP19,MMP7,NOS2,PDGFB,PDGFC,PIK3CD,TF,VIM
Role of Cytokines in Mediating Communication between Immune Cells	4.79E-04	0.111	CSF3,IL12B,IL1A,IL1B,IL1RN,IL33
B Cell Development	5.01E-04	0.139	CD79A,CD79B,HLA-DMB,HLA-DOA,HLA-DOB
PI3K/AKT Signaling	6.76E-04	0.060	CSF2RB,IL10RA,IL18R1,INPP5D,ITGA2,ITGA5,ITGB2,ITGB4,PIK3CD,PTGS2,SFN

ESM Table 5.

List of significant gene ontology terms identified for differentially expressed mRNAs

<table>
<tr><th colspan="4">Supplementary table S5: List of significant gene ontology terms identified for differentially expressed mRNAs</th></tr>
<tr><th>Gene ontology term</th><th>p-value</th><th>Number of genes</th><th>List of genes</th></tr>
<tr><td>exocytosis</td><td>6.81E-04</td><td>19</td><td>Anxa3|Atp2a2|Btk|Cftr|Fgr|Itgam|Itgb2|Lgals9|Anxa1|P2rx1|Rac2|Rest|Syk|Sdc1|Tnfaip2|Wnt7a|Lat2|Rab3il1|Rims4</td></tr>
<tr><td>regulation of regulated secretory pathway</td><td>2.80E-03</td><td>11</td><td>Atp2a2|Fgr|Itgam|Itgb2|Lgals9|P2rx1|Rac2|Rest|Syk|Wnt7a|Rims4</td></tr>
<tr><td>protein secretion</td><td>4.72E-06</td><td>28</td><td>Apoe|Cckar|Cd22|Cftr|Csf1r|Fgr|Gja5|Igf1|Il12b|Il1a|Il1b|Il1rn|Itgb6|Kcnn4|Kcnq1|Anxa1|Nos2|Rest|Syk|Tlr4|Trh|Clec4e|Clec4n|Il33|Tlr9|Ano1|C1qtnf5|Fermt1</td></tr>
<tr><td>regulation of vesicle-mediated transport</td><td>5.46E-07</td><td>33</td><td>Apoe|Arc|Atp2a2|C3|Anxa2|Cd22|Cdh13|Cftr|Cnn2|Cyba|Fcgr2b|Fcgr3|Fgr|Il1b|Itga2|Itgam|Itgb2|Lgals3|Lgals9|Anxa1|P2rx1|Rac2|Rest|Rit2|Sftpd|Syk|Sdc1|Trf|Vtn|Wnt7a|Stap1|Nckap1l|Rims4</td></tr>
<tr><td>endocytosis</td><td>6.06E-07</td><td>39</td><td>Anxa3|Apoe|Arc|C3|Anxa2|Cd22|Cd9|Cdh13|Cnn2|Dmbt1|Cyba|Fcgr2b|Fcgr3|Fgr|Il10ra|Il1b|Itga2|Itgam|Itgb2|Lgals3|Anxa1|Rac2|Rit2|Msr1|Sftpd|Syk|Tgfbr2|Tgm2|Tlr4|Trf|Vav1|Vtn|Stap1|Ifitm3|Epn3|Nckap1l|Colec12|Rin1|Rab7b</td></tr>
<tr><td>phagocytosis</td><td>2.64E-06</td><td>23</td><td>Anxa3|C3|Cnn2|Cyba|Fcgr2b|Fcgr3|Fgr|Il1b|Itga2|Itgam|Itgb2|Anxa1|Rac2|Msr1|Sftpd|Syk|Tgm2|Tlr4|Vav1|Stap1|Nckap1l|Colec12|Rab7b</td></tr>
<tr><td>antigen processing and presentation</td><td>8.63E-04</td><td>9</td><td>Ctse|Ctss|Fcgr2b|Fcgr3|H2-DMb1|H2-Oa|H2-Ob|Rab32|Wdfy4</td></tr>
<tr><td>calcium-mediated signaling</td><td>2.10E-03</td><td>12</td><td>Atp2a2|Cxcr5|Cd22|Cdh13|Edn1|Gsto1|Igf1|Rit2|Syk|Homer2|Lat2|Adgrb2</td></tr>
<tr><td>protein processing</td><td>6.83E-04</td><td>14</td><td>C3|Ctse|Ctsh|Ctss|Cd55|F3|Il1b|Mme|Myc|Klk1b4|Klk1b3|Plau|Fcmr|Nlrc4</td></tr>
<tr><td>positive regulation of oxidative stress-induced cell death</td><td>9.43E-03</td><td>3</td><td>Itgam|Rest|Tlr4</td></tr>
<tr><td>superoxide anion generation</td><td>9.45E-08</td><td>9</td><td>Acp5|Cyba|Cybb|Edn1|Itgam|Itgb2|Syk|Hvcn1|Duox2</td></tr>
<tr><td>cell killing</td><td>3.02E-10</td><td>22</td><td>C3|Ctsc|Ctsh|Cd55|Fcer2a|Fcgr3|Il12b|Itgam|Lgals3|Lgals9|Ltf|Nos2|Reg3g|Cxcl2|Cxcl5|Syk|Pglyrp1|Vav1|Klrk1|Stap1|Hamp2|Raet1e</td></tr>
<tr><td>reactive oxygen species metabolic process</td><td>3.73E-09</td><td>25</td><td>Acp5|Cryab|Cyba|Cybb|Cyp1b1|Edn1|Hk2|Igf1|Il1b|Itgam|Itgb2|Smad3|Ngfr|Nos2|Pdgfb|Plau|Ptgs2|Syk|Tgfbr2|Tlr4|Vav1|Klrk1|Duoxa2|Hvcn1|Duox2</td></tr>
<tr><td>regulation of secretion by cell</td><td>3.87E-09</td><td>43</td><td>Apoe|Atp2a2|Runx1|Cckar|Cd22|Cftr|Csf1r|Edn1|Fgr|Gal|Gja5|Igf1|Il12b|Il1a|Il1b|Il1rn|Itgam|Itgb2|Kcnn4|Kcnq1|Lgals3|Lgals9|Lif|Anxa1|Ngf|Nos2|P2rx1|Rac2|Rest|Syk|Sdc1|Tlr4|Tnfrsf1b|Trh|Wnt7a|Clec4e|Clec4n|Il33|Tlr9|Ano1|Fermt1|Rims4|Rasl10b</td></tr>
<tr><td>stress-activated protein kinase signaling cascade</td><td>6.37E-03</td><td>13</td><td>Cryab|Edn1|Fcgr2b|Fgfr3|Il1a|Il1b|Il1rn|Syk|Tlr4|Trf|Wnt7a|Wnt7b|Tlr9</td></tr>
<tr><td>actin cytoskeleton organization</td><td>1.16E-06</td><td>36</td><td>Avil|Arhgdib|Capg|Casp4|Cckar|Cnn2|Csf1r|Csf3|Edn1|Eln|Epha1|Fgr|Fhl3|S1pr2|Gpm6b|Hcls1|Il1a|Krt19|Stmn1|Anxa1|Smad3|Notch2|Pdgfb|Rac2|Trf|Xirp1|Amot|Pdlim1|Tacstd2|Stap1|Inf2|Cdc42ep1|Nckap1l|Myo5c|Frmd5|Arhgap28</td></tr>
<tr><td>apoptotic signaling pathway</td><td>1.28E-04</td><td>28</td><td>Casp4|Ctsc|Ctsh|Cyp1b1|Epha2|Fgfr3|Gdnf|Ifi204|Igf1|Il1a|Il1b|Itgam|Lcn2|Lgals3|Smad3|Mal|Myc|Ngf|Ngfr|Plaur|Ptgs2|Tnfrsf1b|Vnn1|Sfn|Fcmr|Dapl1|Il33|Cth</td></tr>
<tr><td>glucose transmembrane transport</td><td>9.35E-03</td><td>7</td><td>C3|Edn1|Hk2|Igf1|Il1b|Fabp5|Myc</td></tr>
<tr><td>endocrine pancreas development</td><td>1.96E-03</td><td>6</td><td>Cdk6|Cftr|Anxa1|Ptf1a|Sox9|Onecut2</td></tr>
<tr><td>small GTPase mediated signal transduction</td><td>4.48E-04</td><td>21</td><td>Apoe|Arhgdib|Cdh13|Erbb2|Pdpn|Igf1|Stmn1|Klk1b4|Ngf|Ngfr|Notch2|Rac2|Rit2|Vav1|Amot|Fgd3|Eps8l1|Cyth4|Cdc42ep1|Arhgap30|Arhgap28</td></tr>
<tr><td>inflammatory response</td><td>4.00E-27</td><td>71</td><td>Acp5|Adam8|Apoe|Btk|Ciita|C3|C3ar1|Casp4|Cebpa|Cnr2|Csf1r|Ctsc|Ctss|Cyba|Cybb|Cd55|Ecm1|Epha2|F3|Fcgr2b|Fcgr3|Gal|Lilrb4a|Hyal1|Igf1|Il12b|Il16|Il1a|Il1b|Il1rn|Il18r1|Itga2|Itgam|Itgb2|Itgb6|Lgals9|Anxa1|Ly86|Smad3|Naip6|Nos2|Notch2|P2rx1|Reg3b|Pik3cd|Ptgs2|Reg3g|Ccl6|Ccl7|Ccl9|Cxcl2|Cxcl5|Serpinf1|Syk|Sdc1|Tgm2|Tlr4|Tnfrsf1b|Pglyrp1|Vnn1|Stap1|Serpinb1a|Duoxa2|Sting1|Il33|Tlr9|Pik3ap1|Tlr7|Metrnl|Themis2|Nlrc4</td></tr>
<tr><td>regulation of cytokine production</td><td>1.26E-17</td><td>57</td><td>Acp5|Adam8|Btk|C3|C3ar1|Casp4|Runx1|Csf1r|Cyba|Cybb|Cyp1b1|Epha2|F3|Fcgr2b|Fcgr3|Fgr|Ifi204|Igf1|Il12b|Il16|Il1a|Il1b|Il18r1|Inpp5d|Itgb6|Lgals9|Anxa1|Ltf|Smad3|Nos2|Pou2af1|Ptgs2|Cxcl5|Sftpd|Syk|Tlr4|Tnfrsf1b|Pglyrp1|Homer2|Klrk1|Clec4e|Clec4n|Serpinb1a|Mcoln2|Sting1|Il33|Tlr9|Trim16|Nckap1l|Tlr7|Rab7b|Sulf1|Fermt1|Nlrc4|Ltbp1|Ifi211|Isg15</td></tr>
<tr><td>positive regulation of cytokine production</td><td>2.99E-13</td><td>39</td><td>Adam8|C3|C3ar1|Casp4|Runx1|Csf1r|Cyba|Cybb|Cyp1b1|F3|Fcgr3|Fgr|Ifi204|Il12b|Il16|Il1a|Il1b|Il18r1|Lgals9|Anxa1|Smad3|Pou2af1|Ptgs2|Syk|Tlr4|Klrk1|Clec4e|Clec4n|Mcoln2|Sting1|Il33|Tlr9|Trim16|Tlr7|Rab7b|Sulf1|Fermt1|Nlrc4|Ifi211</td></tr>
</table>

Gene ontology terms highlighted in yellow indicate the terms selected for representation

ESM Table 6.
Checklist for reporting human islet preparations used in research

Islet preparation	1	2	3	4	5	6	7	8[a]
MANDATORY INFORMATION								
Unique identifier	AEL5345 (*SAMN08611141*)	AFA3256 (*SAMN08611211*)	AEIY348 (SAMN08768997)	AEIL428A (*SAMN08768972*)				
Donor age (years)	48	45	24	27				
Donor sex (M/F)	M	M	M	M				
Donor BMI (kg/m^2)	32.4	29.8	38.1	30				
Donor HbA_{1c} or other measure of blood glucose control	5.6	5.1	5.4	5.4				
Origin/source of islets[b]	IIDP program	IIDP program	IIDP program	IIDP program				
Islet isolation centre	*The Scharp-Lacy Research Institute*	*The Scharp-Lacy Research Institute*	*Southern California Islet Cell Resource Center*	*University of Wisconsin*				
Donor history of diabetes? Please select yes/no from drop down list	No	No	No	No				
If Yes, complete the next two lines if this information is available								
Diabetes duration (years)								
Glucose-lowering therapy at time of death[c]								
RECOMMENDED INFORMATION								
Donor cause of death			*Cerebrovascular/stroke*	*Head trauma*				
Warm ischaemia time (h)				*Yes, 32 minutes*				

Cold ischaemia time (h)				*4 Hour(s) 20 Minute(s)*				
Estimated purity (%)				*95%*				
Estimated viability (%)				*99%*				
Total culture time (h)[d]								
Glucose-stimulated insulin secretion or other functional measurement[e]								
Handpicked to purity? Please select yes/no from drop down list								
Additional notes								

Adapted from Hart NJ, Powers AC (2018) Progress, challenges, and suggestions for using human islets to understand islet biology and human diabetes. Diabetologia https://doi.org/10.1007/s00125-018-4772-2

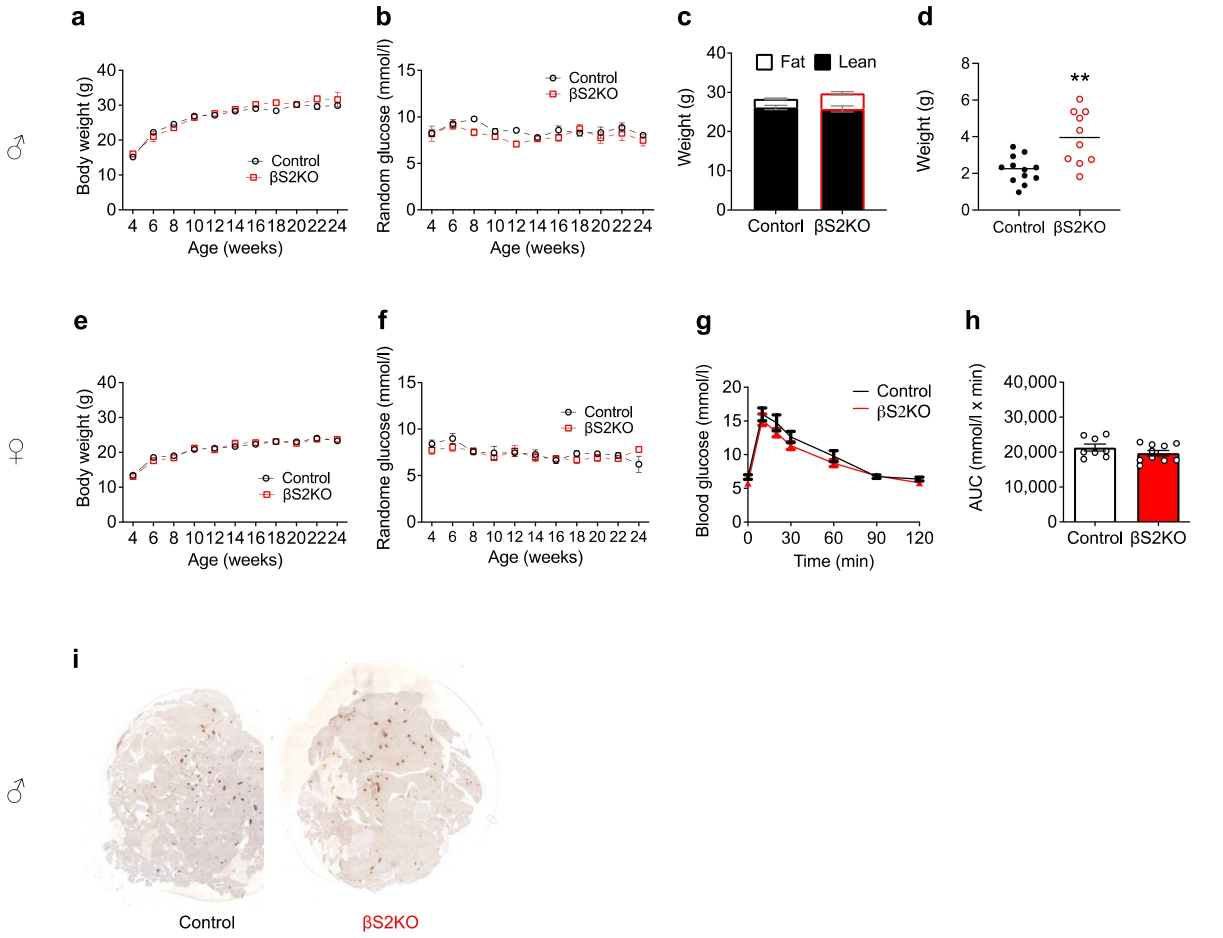

ESM Fig. 1. SERCA2 deficiency did not impact weight, lean mass, or glucose tolerance in female mice.

Beta cell-specific SERCA2 KO (βS2KO) and SERCA2$^{flox/flox}$ mice (Control) were fed a normal chow diet for 25 weeks. Longitudinal changes in whole body weight and random blood glucose in male (A and B) and female mice (E and F) was monitored until 24-week-old, n= at least 6.

(C-D) Lean mass and fat mass were measured in male mice at 25 weeks of age using the EchoMRI 500 Body Composition Analyzer, n=10-12.

(G-H) GTT was performed (2 g/kg glucose dosed to lean mass) in female mice at 24 weeks of age. Area under curve (AUC) analysis is shown graphically (H), n=8-10.

(I) Representative images of insulin immunostaining in pancreatic sections from male control and βS2KO mice at 25 weeks-of-age.

Results are displayed as mean ± SEM. *p < 0.05; **p < 0.01 vs control.

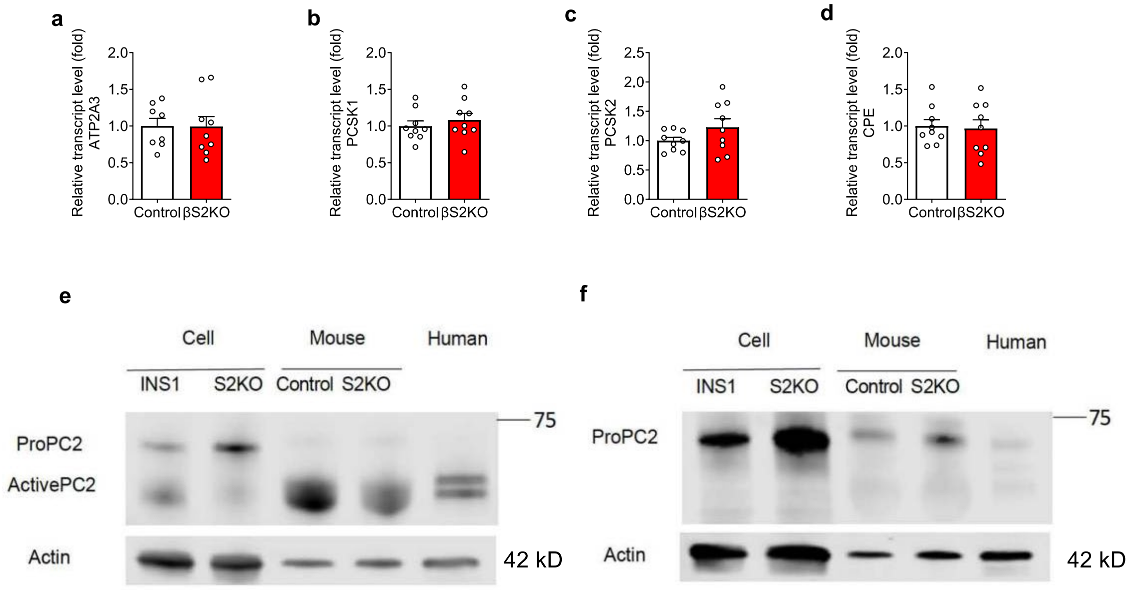

ESM Fig. 2. Beta cell-specific SERCA2 deficiency did not alter transcription of prohormone convertase isoforms in islets.

(A-D) Transcript levels of *ATP2A3*, *PCSK1*, *PCSK2*, and *CPE* in islets of control and βS2KO mice (male, 24-weeks-of-age) were determined by RT-qPCR and normalized to actin levels. Replicates are indicated by open circles and squares. *Indicates statistically significant difference from control (***$p<0.001$) by a Student's *t*-test.

(E-F) Protein lysate from INS1 cells, isolated mouse islets, and human islets were used for western blot analysis. Representative immunoblots of PC2 (E) and ProPC2 (F) with Actin blot, which is the loading control. PC2 propeptide antiserum (LSU26) was raised against residues His^{58}-Asp^{80} of mouse pro-PC2, as described in the Methods.

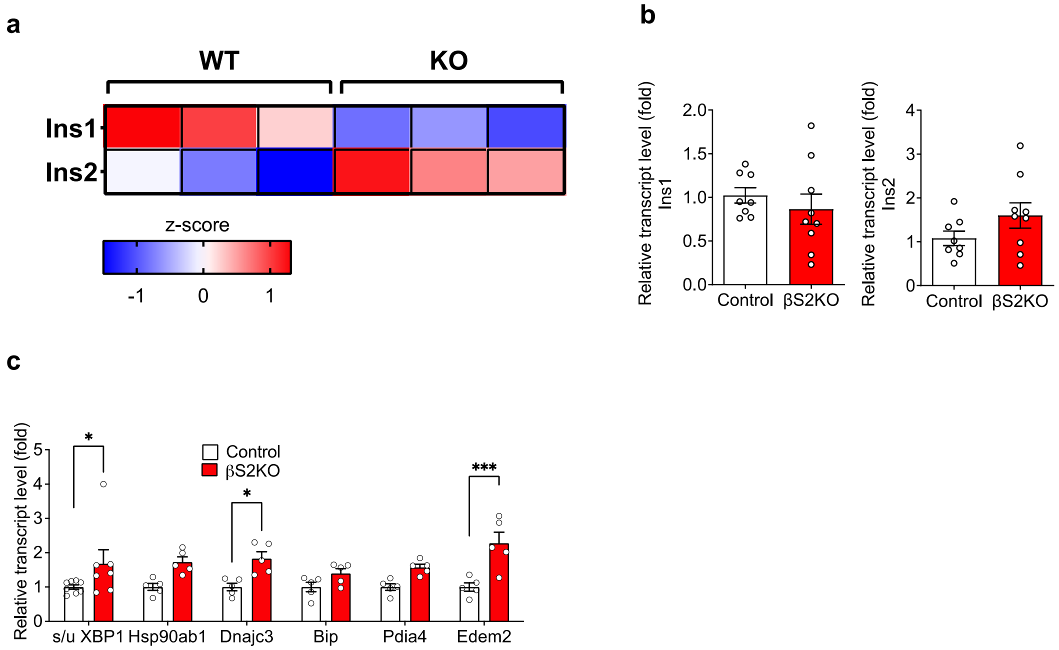

ESM Fig. 3. Markers of ER stress were mildly increased, while insulin gene mRNA levels were not altered in islets isolated from βS2KO mice.

(A) RNA isolated from control and βS2KO islets was subjected to bulk RNA sequencing analysis at 17-weeks of age. Heatmap of the fold changes for insulin genes.

(B) Transcript levels of *Ins1* and *Ins2* in islets of control and βS2KO mice (male, 24 weeks of age) were determined by RT-qPCR and normalized to actin levels. Replicates are indicated by open circles and squares. ns = not significantly different between groups.

(C) Transcript levels of spliced and unspliced *Xbp1, Hsp90ab1, Dnajc3, Bip* (also known as *Hspa5*), *Pdja4,* and *Edem2* were determined by RT-qPCR and normalized to actin levels. Replicates are indicated by open circles and squares. Results are presented as the mean ± S.E.M. *Indicates statistically significant difference (*$p<0.05$, ***$p<0.001$) by a student t-test.

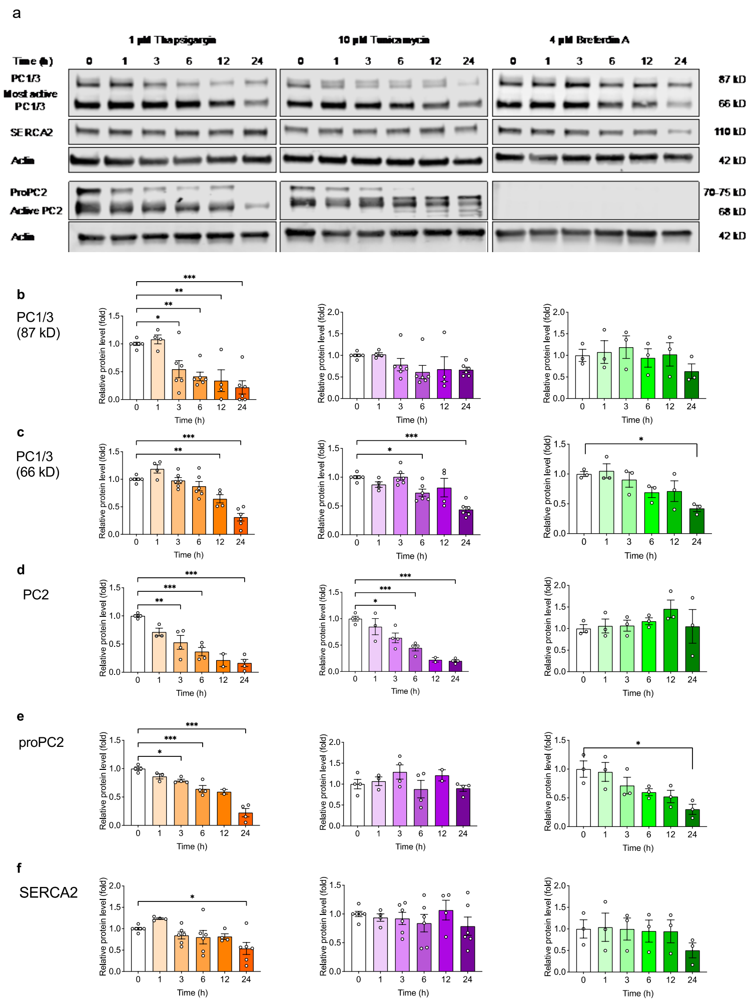

ESM Fig. 4. ER stress and inhibition of protein trafficking reduced active prohormone convertases isoforms in beta cell lines.

(A) Representative immunoblots of PC1/3, SERCA2, and PC2 in INS1 cells treated with 1 umol/L thapsigargin, 10 u mol/L tunicamycin, or 4 u mol/L brefeldin A for 0, 1, 3, 6, 12, or 24 h.

(B-F) Quantitation of immunoblotting results. Expression of proteins was normalized to actin expression; n=2-6. Results are presented as the mean ± S.E.M. Replicate samples are indicated by open circles. *Indicates statistically significant difference (*p<0.05, **p<0.01, ***p<0.001) by a two-way ANOVA and Sidak’s post-test.

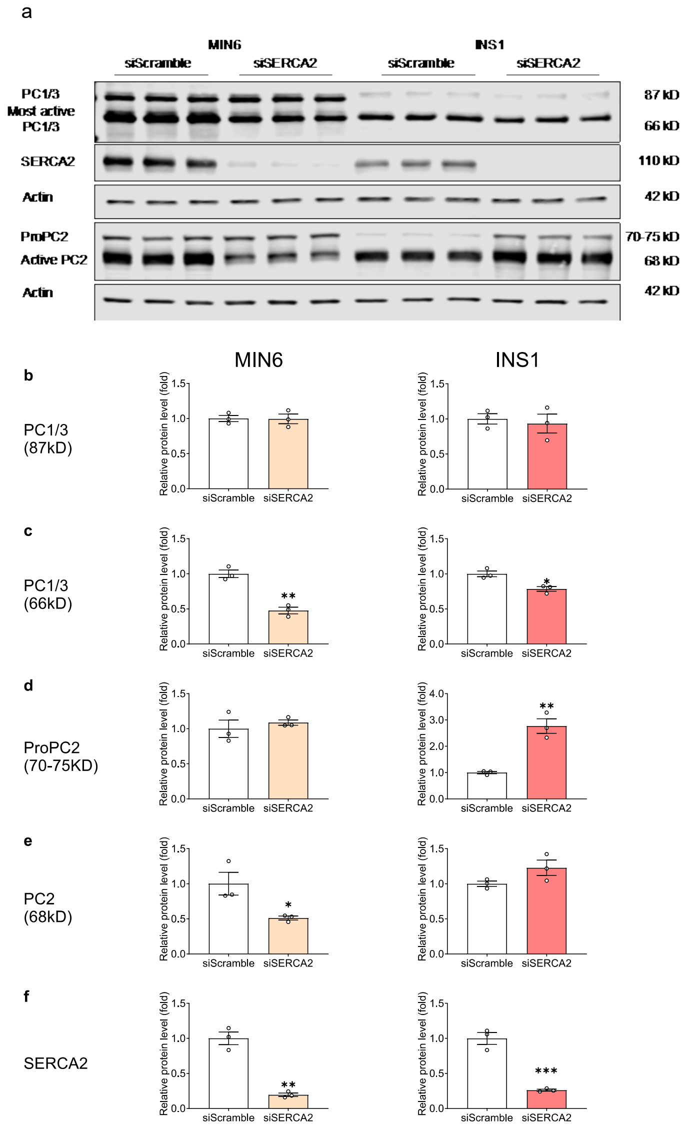

ESM Fig. 5. siRNA-based SERCA2 knockdown reduced active prohormone convertases isoforms in beta cell lines.

(A) Representative immunoblots of PC1/3, SERCA2, and PC2 in MIN6 or INS1 cells treated with siSERCA2 or control siRNA for 48 h.

(B-F) Quantitation of immunoblotting results. Expression of proteins was normalized to actin expression; n=3. Results are presented as the mean ± S.E.M. Replicate samples are indicated by open circles.

Indicates statistically significant difference ($p<0.05$, **$p<0.01$, ***$p<0.001$) by a student t-test.
