# Supplementary material for: SERCA2 regulates proinsulin processing and processing enzyme maturation in pancreatic beta cells
Source: Diabetologia. 2023 Aug 4;66(11):2042–61. doi: 10.1007/s00125-023-05979-4 (PMC10542743; doi:10.1007/s00125-023-05979-4)
Supplement: Supplementary file 1 [file 125_2023_5979_MOESM1_ESM.pdf]

# Electronic Supplementary Material

# SERCA2 regulates proinsulin processing and processing enzyme maturation in the pancreatic beta cell

Hitoshi Iida, Tatsuyoshi Kono, Chih-Chun Lee, Preethi Krishnan, Matthew C. Arvin, Staci A. Weaver, Timothy S. Jarvela, Renato C. S. Branco, Madeline R. McLaughlin, Robert N. Bone, Xin Tong, Peter Arvan, Iris Lindberg, and Carmella Evans-Molina

**Contents:**
**ESM Table 1. KEY RESOURCES**
**ESM Table 2. Human Donor Information**
**ESM Table 3. List of differentially expressed mRNAs (Partial)**
**ESM Table 4. List of significant pathways identified for differentially expressed mRNAs (Partial)**
**ESM Table 5. List of significant gene ontology terms identified for differentially expressed mRNAs**
**ESM Table 6. Checklist for reporting human islet preparations used in research**
**ESM Fig. 1. SERCA2 deficiency did not impact weight, lean mass, or glucose tolerance in female mice.**
**ESM Fig. 2. Beta cell-specific SERCA2 deficiency did not alter transcription of prohormone convertase isoforms in islets.**
**ESM Fig. 3. Markers of ER stress were mildly increased, while insulin gene mRNA levels were not altered in islets isolated from βS2KO mice.**
**ESM Fig.4. ER stress and inhibition of protein trafficking reduced active prohormone convertases isoforms in beta cell lines.**
**ESM Fig. 5. siRNA-based SERCA2 knockdown reduced active prohormone convertases isoforms in beta cell lines.**

**ESM Table 1.**
**Key Resources**

| REAGENT or RESOURCE | SOURCE | IDENTIFIER |
|---|---|---|
| Antibodies | | |
| Rabbit polyclonal anti-PC1/3 | Cell Signaling Technology | Cat# 11914; RRID:AB_2631284) |
| Rabbit polyclonal PC1/3 C-terminal | Gift from Dr. Iris Lindberg, University of Maryland Vindrola et al., 1992 | http://thelindberglab.com/antibodies/ |
| Rabbit monoclonal anti-PC2 | Cell Signaling Technology | Cat# 14013; RRID:AB_2631285 |
| Rabbit polyclonal pro-PC2 | Gift from Dr. Iris Lindberg, University of Maryland Muller et al., 2000 | http://thelindberglab.com/antibodies/ |
| Rabbit polyclonal anti-CPE | Abcam | Cat# ab11044; RRID:AB_297698 |
| Goat polyclonal anti-SERCA2 | Santa Cruz Biotechnology | Cat# sc-8095; RRID:AB_2290108 |
| Mouse monoclonal anti-SERCA2 | Santa Cruz Biotechnology | Cat# sc-376235; RRID:AB_10989947 |
| Mouse monoclonal anti-Actin | Millipore | Cat# MAB1501; RRID:AB_2223041 |
| Mouse monoclonal anti-beta-Actin | Santa Cruz Biotechnology | Cat# sc-47778; RRID:AB_626632 |
| Guinea pig polyclonal anti-Insulin | Agilent Dako | Cat# A0564; RRID:AB_10013624 |
| Mouse monoclonal anti-proInsulin | Developmental Studies Hybridoma Bank | Cat# GS-9A8-C |
| Rabbit monoclonal anti-Insulin (C27C9) | Cell Signaling Technology | Cat# 3014, RRID:AB_2126503 |
| Rabbit monoclonal anti-LMAN1 (ERGIC53) | Abcam | Cat# ab125006; RRID:AB_10973984 |
| Rabbit polyclonal anti-Connexin36 (GJD2) | Novus Biological | Cat# NBP1-59254; RRID:AB_11039152 |
| Goat polyclonal Anti-Giantin | Santa Cruz Biotechnology | Cat# sc-46993; RRID:AB_2279271 |
| IIRDye® 800CW Donkey anti-Mouse IgG Secondary Antibody | LI-COR Bioscience | Cat# 926-32212, RRID:AB_621847 |
| IRDye® 800CW Donkey anti-Goat IgG Secondary Antibody | LI-COR Bioscience | Cat# 926-32214, RRID:AB_621846 |
| IRDye® 680RD Donkey anti-Rabbit IgG Secondary Antibody | LI-COR Bioscience | Cat# 926-68073, RRID:AB_10954442 |
| Donkey anti-guinea pig IgG Secondary Antibody (Alexa Fluor-647) | Jackson ImmunoResearch Laboratories, Inc | Cat# 706-605-148, RRID: AB_2340476 |
| Donkey anti-goat IgG Secondary Antibody (Alexa Fluor-568) | Thermo Fisher Scientific | Cat# A11057, RRID: AB_ 2534104 |
| Donkey anti-rabbit IgG Secondary Antibody (Alexa Fluor-647) | Thermo Fisher Scientific | Cat# A31573, RRID: AB_2536183 |

| | | |
|---|---|---|
| Donkey anti-mouse IgG Secondary Antibody (Alexa Fluor-488) | Thermo Fisher Scientific | Cat# A-21202, RRID: AB_141607 |
| Mouse-on-Mouse IgG Blocking Solution | Vector Laboratories | Cat# MKB-2213-1 |
| Vector® NovaRED™ Substrate Kit, Peroxidase | Vector Laboratories | Cat# SK-4800 |
| Bacterial and virus strains | | |
| adenovirus expressing human SERCA2b | Gift from Dr. Umut Ozcan, Harvard Medical School | N/A |
| adenovirus expressing RIP-D4ER Cameleon probe | Gift from Dr. Richard Benninger, University of Colorado<br>Greotti et al., 2016 | N/A |
| Adenovirus expressing RIP-GCaMP6s | VectorBuilder | N/A |
| | | |
| Biological samples | | |
| Human donor islets | See Supplemental Table S2 | N/A |
| | | |
| Chemicals, peptides, and recombinant proteins | | |
| Insulin (regular, short-acting) | Novo Nordisk | NC0769896 |
| Humulin R | Eli Lilly | NDC 0002-8215-01 |
| D-Glucose | Sigma Aldrich | Cat# G7528 |
| Brefeldin A (BFA) | Sigma Aldrich | Cat# B6542 |
| Thapsigargin (TG) | Cayman Chemical | Cat# 10522 |
| Tunicamycin (TM) | Cayman Chemical | Cat# 11445 |
| Palmitate | Sigma Aldrich | Cat# P0500 |
| Bovine aprotinin | Cayman Chemical | Cat# 14716 |
| Cyclohexamide | Sigma Aldrich | Cat# C4859 |
| MG132 | Sigma Aldrich | Cat# M7449 |
| RPMI1640 | Gibco | Cat# 11875-093 |
| DMEM | Gibco | Cat# 11965-092 |
| Opti Mem I | Gibco | Cat# 31985-070 |
| pERTKR-aminomethylcumarin | Peptide International | Cat# MPR-3159-v |
| Aminomethylcumarin | Sigma Aldrich | Cat# A9891 |
| N-p-tosyl-L-phenylalanine chloromethyl ketone | Sigma Aldrich | Cat# T4376 |
| trans-epoxysuccinyl-L-leucylamido(4-guanidino) butane | Sigma Aldrich | Cat# 66701 |
| Nα-tosyl-L-lysine chloromethyl ketone | Sigma Aldrich | Cat# T7254 |
| 7B2-CT peptide | Gift from Dr. Iris Lindberg, University of Maryland | N/A |

| | | |
|---|---|---|
| ProSAAS-CT peptide | Gift from Dr. Iris Lindberg, University of Maryland | N/A |
| Lipofectamin RNAiMAX | Thermo Fisher Scientific | Cat# 13778-075 |
| Moloney murine leukaemia virus reverse transcriptase | Thermo Fisher Scientific | Cat# 28025013 |
| DC protein assay kit II | Bio-Rad | Cat# 5000112 |
| 4-20% Mini-Protean TGX gel | Bio-Rad | Cat# 4561096 |
| Odyssey blocking buffer | LI-COR Biosciences | Cat# 927-40000 |
| Signal Enhancer HIKARI 250 | nacalai tesque | Cat# 2994 |
| Bovine Aprotinin | Cayman Chemical | Cat# 14716 |
| Animal-Free Blocker and Diluent, R.T.U. | Vector Laboratories | Cat# SP-5035-100 |
| FluorSave Reagent | Merck Millipore | Cat# 345789 |
| cOmplete protease inhibitor cocktail, EDTA-free | Roche | Cat# 04693132001 |
| PhosSTOP | Roche | Cat# 4906845001 |
| TRIS | Sigma Aldrich | Cat# 11814273001 |
| Deoxycholate | Sigma Aldrich | Cat# 6750 |
| IGEPAL CA-630 | Sigma Aldrich | Cat# I8896 |
| SDS | Sigma Aldrich | Cat# L3771 |
| Sarcosyl, | Sigma Aldrich | Cat# L7414 |
| Glycerol | Sigma Aldrich | Cat# G5516 |
| Dithiothreitol | Roche | Cat# 10197777001 |
| EDTA | Sigma Aldrich | Cat# E9884 |
| Sodium flouride | Sigma Aldrich | Cat# 201154 |
| $MgCl_2$ | Sigma Aldrich | Cat# M8266 |
| Benzonase nuclease | Millipore | Cat# 70746-3CN |
| | | |
| Critical commercial assays | | |
| RNeasy Mini Kit | Qiagen | Cat# 74136 |
| RNeasy Micro Kit | Qiagen | Cat# 74034 |
| DNeasy Blood and Tissue kit | Qiagen | Cat# 69504 |
| SensiFASTÔSYBR Lo-ROX kit | Bioline | Cat# BIO-94020 |
| BCA protein determination | BioRad | Cat# 500-0112 |
| Mouse Insulin ELISA | Mercodia | Cat# 10-1247-10 |
| Fura-2 acetoxymethylester | Invitrogen | Cat# F1221 |
| Mouse ProInsulin ELISA | Alpco Diagnostics | Cat# 80-PINMS-E01 |
| KAPA mRNA Hyper Prep Kit | Roche | Cat# KK8540 |
| | | |
| Experimental models: Cell lines | | |
| Rat (male) SERCA2 null INS-1 832/13 | This paper | N/A |
| Rat (male) INS-1 832/13 | H E Hohmeier et al., 2000 | N/A |

| | | |
|---|---|---|
| Mouse (sex unspecified) MIN6 | J Miyazaki et al, 1990 | N/A |
| | | |
| Experimental models: Organisms/strains | | |
| Mouse: β cell specific SERCA2-null (βS2KO) in C57BL6/J background | This paper | N/A |
| Mouse: C57BL6/J | The Jackson Laboratory | JAX # 000664 |
| Teklad 2018SX | Inotivco | N/A |
| Oligonucleotides | | |
| Mouse PC1/3-F | This paper | AGTTGGAGGCATAAGAATGCTG |
| Mouse PC1/3-R | This paper | GCCTTCTGGGCTAGTCTGC |
| Mouse PC2-F | This paper | AGAGAGACCCCAGGATAAAGATG |
| Mouse PC2-R | This paper | CTTGCCCAGTGTTGAACAGGT |
| Mouse CPE-F | This paper | GCTCAGGTAATTGAAGTCTT |
| Mouse CPE-R | This paper | TACTGCTCACGAATACAGTT |
| Mouse SERCA2b-F | This paper | GATCCTCTACGTGGAACCTTTG |
| Mouse SERCA2b-R | This paper | CCACAGGGAGCAGGAAGAT |
| Mouse SERCA3-F | This paper | AGGGGAAGCTAAGAAGCCAG |
| Mouse SERCA3-R | This paper | CCCTCAGACTCCTCCTACCC |
| Mouse beta-actin-F | This paper | AGGTCATCACTATTGGCAACGA |
| Mouse beta-actin-R | This paper | CACTTCATGATGGATTGAATGTAGTT |
| Mouse spriced-XBP1-F | This paper | CTGAGTCCGAATCAGGTGCAG |
| Mouse spriced-XBP1-R | This paper | GTCCATGGGAAGATGTTCTGG |
| Mouse unspriced-XBP1-F | This paper | TGGCCGGGTCTGCTGAGTCCG |
| Mouse unspriced-XBP1-R | This paper | GTCCATGGGAAGATGTTCTGG |
| Mouse HSP90ab1-F | This paper | TGTATGTACGCCGCGTATTCA |
| Mouse HSP90ab1-R | This paper | TCGGAATCCACAACACCTTTG |
| Mouse Dnajc3-F | This paper | GACAGCTAGCCGACGCCTTA |
| Mouse Dnajc3-R | This paper | GTCACCATCAACTGCAGCGT |
| Mouse Bip-F | This paper | TTCAGCCAATTATCAGCAAACTCT |
| Mouse Bip-R | This paper | TTTTCTGATGTATCCTCTTCACCAGT |
| Mouse Pdia4-F | This paper | TGACCCGGCCTACTTGCA |
| Mouse Pdia4-R | This paper | GTGTGGTGAAACTTGTAATCTTCTCTCA |
| Mouse Edem2-F | This paper | ATGCCTTTCCGGCTACTCATC |
| Mouse Edem2-R | This paper | CCTTGACTCGCTCCCTGTAGT |
| Atp2a2 Mouse siRNA Oligo Duplex | OriGene | CAT#: SR421850<br>GCAGUUUGAAGACUUACUAGUUAGA |
| Software and algorithms | | |
| Zen Blue edition ver2.3 | Carl Zeiss | https://www.zeiss.com/microscopy/int/products/microscope-software.html; RRID:SCR_013672 |
| Axio-Vision Software | Carl Zeiss | https://www.zeiss.com/microscopy/int/products/microscope-software.html; RRID:SCR_002677 |
| ImageJ ver1.52p | Fiji; Schneider et al., 2012 | Open source: https://imagej.net/Fiji; RRID:SCR_002285 |
| Image Studio | LI-COR | https://www.licor.com/bio/image-studio-lite/download; RRID:SCR_013715 |

| | | |
|---|---|---|
| FastQC | Babraham Bioinformatics | https://www.bioinformatics.babraham.ac.uk/projects/download.html RRID:SCR_011106 |
| Prism 7.0 | GraphPad Software | https://www.graphpad.com/; RRID:SCR_002798 |
| Flow software version 10.0.20.1231 | Partek | https://www.partek.com/partek-flow/; RRID:SCR_011860 |
| STAR aligner ver. 2.7.3a | Dobin A et al., 2013 | https://github.com/alexdobin/STAR/releases; RRID:SCR_004463 |
| RefSeq release 93 | NCBI | https://www.ncbi.nlm.nih.gov/refseq/; RRID:SCR_003496 |
| DESeq2 | Bioconductor | Open source: https://bioconductor.org/packages/release/bioc/html/DESeq2.html; RRID:SCR_015687 |
| Ingenuity Pathway Analysis | Qiagen | https://digitalinsights.qiagen.com/product-login/; RRID:SCR_008653 |
| Metascape | Metascape | https://metascape.org; RRID:SCR_016620 |
| ggplot2 | MIT | https://ggplot2.tidyverse.org; RRID:SCR_014601 |
| CellProfiler 4.1.3 | Cellprofiler.org | https://cellprofiler.org/previous-releases RRID:SCR_007358 |
| Qubit Fluorometer | Thermo Fisher Scientific | RRID:SCR_020553 |
| | | |
| | | |
| Perifusion System | Biorep Technologies, Inc | N/A |
| LSM 800 confocal imaging system | Carl Zeiss | RRID:SCR_015963 |
| LSM-700 confocal microscope | Carl Zeiss | RRID:SCR_017377 |
| Bioanalyzer 2100 | Agilent | Cat# G2939BA; RRID:SCR_019715 |
| HiSeq 4000 sequencer | Illumina | RRID:SCR_016386 |
| Odyssey CLx scanner | LI-COR | RRID:SCR_014579 |
| EchoMRI-500 | EchoMRI | RRID:SCR_017104 |
| Contour | Bayer | N/A |
| QuantStudio 3 thermal cycle | Applied Biosystems | RRID:SCR_018712 |
| Zeiss Lightsheet Z.1 Lightsheet Fluorescence Microscope | Carl Zeiss | RRID:SCR_020919 |
| Zeiss LSM 880 with Airyscan Confocal Laser Scanning Microscope | Carl Zeiss | RRID:SCR_020925 |
| SpectraMax M5 plate reader | Molecular Devices | RRID:SCR_020300 |
| SpectraMax iD5 plate reader | Molecular Devices | N/A |
| Mini-Protean Tetra apparatus | Bio-Rad | Cat# 1658005JA |
| Odyssey 1828 Scanner | LI-COR Biosciences | RRID:SCR_014579 |
| | | |
| | | |

## ESM Table 2.
## Human Donor Information

| Arrival Date | Unique Identifier | Donor UNOS ID | Gender | Ethnicity/Race | Age | BMI | Diabetic donor status | Islett purity | Islet viability |
|---|---|---|---|---|---|---|---|---|---|
| | | | | | | | | | |
| 2018.1.10 | AEL5345 | AEL5345 | M | Hispanic/Latino | 48 | 32.4 | No | 95 | 95 |
| 2018.2.5 | AFA3256 | AEDZ307 | M | White | 45 | 29.8 | No | 95 | 95 |
| 2017.10. | RRID:SAMN08768997 | AEIY348 | M | Native Hawaiian | 24 | 38.1 | No | 50 | 98 |
| 2017.9 | AEIL428A | AEIL428A | M | White | 27 | 30 | No | 95 | 99 |

## ESM Table 3.
## List of differentially expressed mRNAs (Partial)

| Supplementary Table S3: List of differentially expressed mRNAs | | | | | | | | | | | |
|---|---|---|---|---|---|---|---|---|---|---|---|
| mRNA | Linear scale fold change | pvalue | mRNA | Linear scale fold change | pvalue | mRNA | Linear scale fold change | pvalue | mRNA | Linear scale fold change | pvalue |
| Cd22 | -22.12 | 2.37E-05 | Msr1 | -4.48 | 1.55E-03 | Nlrc5 | -1.69 | 6.83E-04 | Tgfbi | -1.54 | 6.18E-03 |
| Pax5 | -22.05 | 3.09E-03 | Klrk1 | -4.44 | 4.32E-02 | Amot | -1.69 | 1.05E-02 | Ifitm3 | -1.54 | 7.47E-03 |
| Timd4 | -19.74 | 2.94E-03 | Sp110 | -4.38 | 3.14E-02 | Cnn2 | -1.69 | 2.17E-02 | Gpat3 | -1.53 | 3.41E-02 |
| H2-Ob | -11.87 | 1.22E-02 | C3 | -4.38 | 9.35E-03 | Eps8l1 | -1.69 | 2.68E-03 | Tgfbr2 | -1.53 | 1.50E-02 |
| Fcmr | -11.64 | 2.03E-03 | Plac8 | -4.36 | 2.97E-02 | Usp43 | -1.68 | 3.33E-02 | Acot7 | -1.53 | 1.45E-02 |
| Fcrl1 | -11.46 | 5.97E-03 | Slc9a4 | -4.33 | 3.58E-04 | S100a16 | -1.68 | 2.38E-02 | Tinagl1 | -1.53 | 2.75E-02 |
| Cd37 | -11.16 | 9.42E-04 | Xlr4a | -4.23 | 9.68E-05 | Inpp5d | -1.67 | 4.68E-02 | Avpi1 | -1.53 | 1.22E-02 |
| Slpi | -11.06 | 2.00E-05 | Cd52 | -4.23 | 5.99E-03 | Lilr4b | -1.67 | 1.94E-03 | Adgre5 | -1.52 | 1.39E-02 |
| Xlr4b | -10.84 | 3.11E-08 | Gja5 | -4.20 | 1.73E-02 | Card10 | -1.66 | 3.37E-02 | Bgn | -1.51 | 3.65E-03 |
| Cr2 | -10.45 | 1.05E-03 | Gsta3 | -4.17 | 2.13E-06 | Btc | -1.66 | 3.03E-02 | Tnfaip2 | -1.51 | 9.00E-03 |
| Slc26a9 | -9.87 | 1.04E-04 | Slc39a4 | -4.14 | 5.83E-04 | Lilrb4a | -1.66 | 6.00E-03 | Pkp3 | -1.51 | 3.47E-02 |
| Pou2af1 | -9.77 | 3.78E-02 | Cxcl5 | -4.10 | 1.12E-02 | Serpinf1 | -1.65 | 4.19E-02 | Sh3bgrl3 | -1.51 | 6.88E-03 |
| Ly6d | -9.52 | 5.36E-05 | Themis2 | -4.05 | 3.20E-03 | Hyal1 | -1.65 | 2.76E-02 | Fxyd3 | -1.51 | 2.72E-02 |
| Wfdc2 | -9.47 | 9.01E-05 | Lair1 | -4.03 | 6.17E-03 | Tuba1c | -1.65 | 1.74E-03 | Arhgap28 | -1.51 | 4.58E-02 |
| Cxcr5 | -9.21 | 5.95E-04 | Slc9a2 | -4.02 | 3.50E-04 | Plau | -1.64 | 4.99E-03 | Myof | -1.51 | 5.23E-03 |
| Ifi214 | -8.99 | 2.93E-03 | Pglyrp1 | -4.01 | 3.18E-03 | Ano1 | -1.64 | 2.01E-02 | Cadm3 | -1.50 | 7.18E-03 |
| Folr2 | -8.81 | 1.06E-02 | Ccl9 | -4.00 | 5.09E-04 | Shisa4 | -1.64 | 2.89E-02 | Rit2 | -1.50 | 2.75E-02 |
| Tacstd2 | -8.57 | 5.09E-03 | Klk1b3 | -4.00 | 2.40E-02 | Blnk | -1.64 | 4.97E-02 | Tnfrsf1b | -1.50 | 2.19E-02 |
| Clec4n | -8.47 | 1.04E-02 | Adgrb2 | -3.99 | 7.78E-03 | C3ar1 | -1.63 | 1.34E-02 | Cdk6 | -1.50 | 1.61E-02 |
| Gm2663 | -8.42 | 2.64E-03 | Kctd14 | -3.93 | 6.43E-03 | Epha1 | -1.63 | 2.13E-02 | Cfap61 | 1.53 | 3.20E-02 |
| Cd79b | -8.15 | 2.00E-02 | Gm5771 | -3.91 | 2.94E-02 | F3 | -1.63 | 6.50E-03 | Dapl1 | 1.53 | 2.72E-02 |
| Anxa8 | -8.01 | 2.96E-05 | C1qtnf5 | -3.85 | 3.30E-02 | Nes | -1.63 | 2.82E-04 | Arhgap36 | 1.54 | 1.60E-02 |
| Spink4 | -8.00 | 2.43E-04 | Vnn3 | -3.81 | 1.54E-02 | Mpeg1 | -1.63 | 1.11E-02 | Rasl10b | 1.54 | 6.64E-03 |
| Duox2 | -7.94 | 4.63E-03 | Trim30b | -3.80 | 3.04E-02 | Onecut2 | -1.62 | 5.45E-03 | Frmd5 | 1.55 | 1.09E-03 |
| Ltf | -7.93 | 2.50E-02 | Mpzl2 | -3.79 | 1.12E-04 | Ctss | -1.62 | 2.95E-02 | Gm14288 | 1.58 | 5.04E-03 |
| Wnt7a | -7.60 | 4.20E-04 | Phf11b | -3.78 | 1.59E-02 | Itga2 | -1.61 | 9.34E-04 | Cpne4 | 1.58 | 4.50E-02 |
| Serpinb5 | -7.53 | 2.13E-03 | Il33 | -3.77 | 3.48E-02 | Itgb6 | -1.60 | 7.20E-03 | Dach2 | 1.59 | 2.15E-02 |
| Wdfy4 | -7.43 | 1.63E-03 | Tmc5 | -3.77 | 1.35E-04 | Myc | -1.60 | 9.57E-03 | Trpc4 | 1.62 | 1.98E-03 |
| Btk | -7.22 | 1.56E-03 | Arhgap15 | -3.72 | 4.55E-02 | Cldn2 | -1.60 | 3.94E-02 | AI182371 | 1.63 | 2.36E-02 |
| Ugt1a1 | -7.22 | 2.43E-05 | Arhgap45 | -3.69 | 1.76E-02 | Smad3 | -1.60 | 3.86E-03 | Gm37240 | 1.83 | 4.77E-02 |
| H2-Oa | -7.08 | 2.69E-02 | Reg3g | -3.67 | 5.96E-04 | Il18r1 | -1.59 | 2.21E-02 | Adam32 | 1.90 | 3.86E-02 |
| Mmp7 | -6.96 | 1.64E-02 | Cd79a | -3.65 | 6.53E-04 | Mlph | -1.59 | 7.34E-05 | Kcnh5 | 1.98 | 2.25E-02 |
| Gabrp | -6.92 | 2.12E-03 | Ly86 | -3.64 | 1.83E-02 | Gvin1 | -1.59 | 9.25E-03 | Rbm20 | 2.06 | 1.52E-06 |
| Napsa | -6.43 | 5.64E-03 | Klk1b4 | -3.62 | 1.82E-02 | Gdnf | -1.59 | 2.15E-02 | Vmn2r29 | 2.16 | 4.20E-02 |
| Fgr | -6.17 | 1.22E-02 | Atp2a2 | -3.61 | 8.75E-71 | Olfml2a | -1.59 | 2.70E-02 | Il12b | 2.32 | 1.73E-02 |
| Slc10a2 | -5.96 | 1.66E-04 | Klhdc7a | -3.56 | 3.57E-02 | Ltbp1 | -1.59 | 1.57E-02 | Gm3500 | 2.36 | 2.90E-02 |
| Duoxa2 | -5.94 | 7.66E-05 | Cmah | -3.54 | 2.94E-02 | Col18a1 | -1.58 | 8.74E-03 | Pmfbp1 | 3.15 | 3.40E-02 |
| Traf3ip3 | -5.81 | 3.27E-02 | Epn3 | -3.54 | 5.33E-04 | St3gal4 | -1.58 | 3.74E-02 | Gm20517 | 3.94 | 7.71E-03 |
| Fcna | -5.53 | 1.15E-02 | Egr4 | -3.53 | 3.89E-02 | Prph | -1.58 | 3.70E-03 | Apob | 4.61 | 1.76E-02 |
| Cnr2 | -5.50 | 2.85E-02 | Adgrg7 | -3.53 | 1.52E-02 | Itga5 | -1.58 | 5.68E-03 | Apol7c | 5.09 | 3.11E-02 |
| Lcn2 | -5.35 | 9.06E-04 | Lamb3 | -3.46 | 4.43E-04 | Igfbp4 | -1.58 | 6.51E-03 | Clca1 | 8.95 | 9.36E-03 |
| Trim30c | -5.27 | 3.13E-02 | Cd300ld3 | -3.45 | 1.93E-02 | Apoe | -1.58 | 2.73E-03 | | | |
| Reg3b | -5.26 | 1.62E-03 | Dsg3 | -3.41 | 1.39E-02 | Metrnl | -1.57 | 3.01E-02 | | | |
| ccdc198 | -5.23 | 1.99E-02 | Krt23 | -3.40 | 1.56E-08 | Htr3a | -1.57 | 6.86E-03 | | | |
| Itgam | -5.19 | 3.91E-06 | Nos2 | -3.40 | 1.72E-02 | Fxyd5 | -1.56 | 2.98E-02 | | | |
| Sema3a | -5.07 | 3.45E-02 | Tmem171 | -3.38 | 1.40E-02 | Procr | -1.56 | 6.59E-04 | | | |
| Ctse | -5.05 | 2.18E-04 | Cdh20 | -3.38 | 1.95E-02 | Rest | -1.56 | 2.13E-02 | | | |
| Hamp2 | -5.03 | 6.36E-03 | Trh | -3.38 | 4.29E-03 | Kcnq5 | -1.56 | 2.30E-02 | | | |
| Cfb | -4.97 | 3.90E-02 | Tns4 | -3.37 | 4.24E-02 | Itpripl2 | -1.56 | 3.33E-03 | | | |
| Reg2 | -4.85 | 1.39E-03 | Bglap3 | -3.36 | 2.54E-02 | Ngf | -1.56 | 3.26E-02 | | | |
| Gjb4 | -4.85 | 2.52E-03 | C4b | -3.35 | 1.27E-05 | Vim | -1.55 | 9.19E-03 | | | |
| Tdh | -4.82 | 2.91E-02 | Itgb2 | -3.35 | 4.89E-04 | Pdgfb | -1.55 | 9.54E-04 | | | |
| Car9 | -4.74 | 2.88E-02 | Raet1e | -3.35 | 2.45E-02 | Cd9 | -1.55 | 1.00E-03 | | | |
| Sema3d | -4.67 | 1.21E-02 | Pzp | -3.34 | 3.01E-02 | Avil | -1.55 | 2.74E-03 | | | |
| Mcoln2 | -4.66 | 2.81E-02 | S100a14 | -3.33 | 1.14E-03 | Col12a1 | -1.54 | 8.55E-03 | | | |
| Wnt7b | -4.66 | 3.05E-05 | Ugt1a7c | -3.33 | 3.38E-05 | Plpp2 | -1.54 | 1.70E-02 | | | |
| Gsta4 | -4.59 | 3.33E-06 | Myo5c | -3.32 | 3.54E-02 | Gpm6b | -1.54 | 1.48E-02 | | | |
| Lrmp | -4.52 | 3.43E-03 | Rac2 | -3.30 | 2.22E-02 | Epha2 | -1.54 | 2.76E-02 | | | |

RNA isolated at 17-weeks of age from control and βS2KO islets was subjected to bulk RNA sequencing analysis. Sequencing files were analyzed using the Flow software. The raw sequencing files were aligned to the mouse genome (mm10) using STAR aligner. Uniquely mapped reads were annotated using RefSeq. mRNAs with a total of at least 10 read counts were retained for further analysis. mRNAs with linear scale fold-change $\geq 1.5$ and $p < 0.05$ were identified using DESeq2 and were considered as differentially expressed mRNAs. Biological pathways were identified using QIAGEN Ingenuity Pathway Analysis, and gene ontology enrichment was performed using Metascape. Functional terms with $p < 0.05$ were considered significant.

# ESM Table 4.

# List of significant pathways identified for differentially expressed mRNAs (Partial)

**Supplementary table S4: List of significant pathways identified for differentially expressed mRNAs**

| Ingenuity Canonical Pathways | p-value | Gene Ratio | Molecules |
|---|---|---|---|
| Phagosome Formation | 6.31E-12 | 0.143 | C3AR1,CR2,FCER2,FCGR2A,FCGR2B,ITGA2,ITGA5,ITGAM,ITGB2,ITGB4,MSR1,PIK3CD,RAC2,SCARA3,SYK,TLR4,TLR7,TLR9,VTN |
| TREM1 Signaling | 1.29E-08 | 0.160 | CIITA,CXCL3,FCGR2B,IL1B,ITGA5,LAT2,Naip1 (includes others),NLRC4,NLRC5,TLR4,TLR7,TLR9 |
| STAT3 Pathway | 8.32E-06 | 0.089 | CSF2RB,FGFR2,FGFR3,IGF1,IL10RA,IL18R1,IL1A,IL1B,MYC,NGFR,PDGFB,TGFBR2 |
| MSP-RON Signaling Pathway | 1.12E-05 | 0.138 | CSF2RB,IL12B,ITGAM,ITGB2,KLK3,NOS2,PIK3CD,TLR4 |
| Macropinocytosis Signaling | 1.12E-05 | 0.118 | CSF1R,ITGA5,ITGB2,ITGB4,ITGB6,NGF,PDGFB,PDGFC,PIK3CD |
| PTEN Signaling | 4.79E-05 | 0.081 | FGFR2,FGFR3,INPP5D,ITGA2,ITGA5,ITGB2,ITGB4,NGFR,PIK3CD,RAC2,TGFBR2 |
| Caveolar-mediated Endocytosis Signaling | 6.17E-05 | 0.110 | CD55,FLNC,ITGA2,ITGA5,ITGAM,ITGB2,ITGB4,ITGB6 |
| GP6 Signaling Pathway | 5.50E-04 | 0.072 | APBB1IP,BTK,COL12A1,COL18A1,COL6A5,LAMB3,PIK3CD,SYK,VAV1 |
| Antigen Presentation Pathway | 7.41E-04 | 0.128 | CIITA,HLA-DMB,HLA-DOA,HLA-DOB,NLRC5 |
| Type I Diabetes Mellitus Signaling | 1.10E-03 | 0.072 | HLA-DMB,HLA-DOA,HLA-DOB,IL12B,IL1B,NGFR,NOS2,TNFRSF1B |
| Phagosome Maturation | 7.24E-03 | 0.053 | CTSC,CTSE,CTSH,CTSS,CYBB,RAB7B,TUBA1C,TUBB2B |
| HIPPO signaling | 2.09E-02 | 0.059 | AMOT,SFN,SMAD3,TEAD2,WWC1 |
| Glutathione-mediated Detoxification | 2.09E-02 | 0.094 | GSTA3,Gsta4,GSTO1 |
| Autophagy | 2.63E-02 | 0.066 | CTSC,CTSE,CTSH,CTSS |
| Calcium-induced T Lymphocyte Apoptosis | 3.39E-02 | 0.061 | ATP2A2,HLA-DMB,HLA-DOA,HLA-DOB |
| Clathrin-mediated Endocytosis Signaling | 1.00E-03 | 0.057 | APOB,APOE,IGF1,ITGA5,ITGB2,ITGB4,ITGB6,PDGFB,PDGFC,PIK3CD,TF |
| NF-κB Signaling | 1.35E-06 | 0.084 | CARD10,FGFR2,FGFR3,IL1A,IL1B,IL1RN,IL33,NGF,NGFR,PIK3CD,TGFBR2,TLR4,TLR7,TLR9,TNFRSF1B |
| Glucocorticoid Receptor Signaling | 2.00E-05 | 0.050 | ANXA1,BGLAP,CEBPA,CSF2RB,CXCL3,IGF1,IL10RA,IL18R1,IL1A,IL1B,IL1RN,KRT17,KRT19,KRT23,KRT80,MYC,NOS2,PIK3CD,PLAU,PTGS2,SLPI,SMAD3,TGFBR2 |
| Role of NFAT in Regulation of the Immune Response | 5.89E-04 | 0.061 | BLNK,BTK,CD79A,CD79B,FCGR2A,FCGR2B,HLA-DMB,HLA-DOA,HLA-DOB,PIK3CD,SYK |
| Sphingosine-1-phosphate Signaling | 2.19E-02 | 0.051 | CASP4,PDGFB,PDGFC,PIK3CD,RAC2,S1PR2 |
| LXR/RXR Activation | 1.00E-10 | 0.140 | APOB,APOE,C3,C4A/C4B,IL1A,IL1B,IL1RN,IL33,MSR1,NGFR,NOS2,PTGS2,SERPINF1,TF,TLR4,TNFRSF1B,VTN |
| Granulocyte Adhesion and Diapedesis | 6.03E-10 | 0.110 | Ccl6,Ccl7,Ccl9,CLDN2,CLDN8,CSF3,CXCL3,CXCL6,IL1A,IL1B,IL1RN,IL33,ITGAM,ITGB2,MMP19,MMP7,NGFR,SDC1,TNFRSF1B |
| Hepatic Fibrosis / Hepatic Stellate Cell Activation | 1.29E-08 | 0.097 | COL12A1,COL18A1,COL6A5,CXCL3,EDN1,FGFR2,IGF1,IGFBP4,IL10RA,IL1A,IL1B,NGFR,PDGFB,PDGFC,SMAD3,TGFBR2,TLR4,TNFRSF1B |
| Complement System | 1.86E-08 | 0.243 | C1QC,C3,C3AR1,C4A/C4B,CD55,CFB,CR2,ITGAM,ITGB2 |
| Communication between Innate and Adaptive Immune Cells | 2.14E-07 | 0.125 | Ccl9,CD79A,CD79B,IL12B,IL1A,IL1B,IL1RN,IL33,TLR4,TLR7,TLR9,TNFSF13 |
| PI3K Signaling in B Lymphocytes | 3.02E-07 | 0.101 | BLNK,BTK,C3,CARD10,CD79A,CD79B,CR2,FCGR2B,INPP5D,PIK3AP1,PIK3CD,SYK,TLR4,VAV1 |
| Dendritic Cell Maturation | 3.63E-07 | 0.087 | COL18A1,FCGR2A,FCGR2B,HLA-DMB,HLA-DOA,HLA-DOB,IL12B,IL1A,IL1B,IL1RN,IL33,NGFR,PIK3CD,TLR4,TLR9,TNFRSF1B |
| B Cell Receptor Signaling | 4.17E-07 | 0.086 | APBB1IP,BLNK,BTK,CARD10,CD22,CD79A,CD79B,FCGR2A,FCGR2B,INPP5D,PAX5,PIK3AP1,PIK3CD,RAC2,SYK,VAV1 |
| FXR/RXR Activation | 6.46E-07 | 0.103 | APOB,APOE,C3,C4A/C4B,IL1A,IL1B,IL1RN,IL33,SDC1,SERPINF1,SLC10A2,TF,VTN |
| Agranulocyte Adhesion and Diapedesis | 6.92E-07 | 0.083 | Ccl6,Ccl7,Ccl9,CLDN2,CLDN8,CXCL3,CXCL6,IL1A,IL1B,IL1RN,IL33,ITGA2,ITGA5,ITGB2,MMP19,MMP7 |
| T Helper Cell Differentiation | 9.55E-07 | 0.137 | CXCR5,HLA-DMB,HLA-DOA,HLA-DOB,IL10RA,IL12B,IL18R1,NGFR,TGFBR2,TNFRSF1B |
| MSP-RON Signaling In Macrophages Pathway | 1.29E-06 | 0.106 | CIITA,HLA-DMB,HLA-DOA,HLA-DOB,IL12B,ITGAM,ITGB2,KLK3,NOS2,PIK3CD,PTGS2,TLR4 |
| Th1 and Th2 Activation Pathway | 3.98E-06 | 0.082 | HLA-DMB,HLA-DOA,HLA-DOB,IL10RA,IL12B,IL18R1,IL33,ITGB2,NOTCH2,PIK3CD,SPI1,TGFBR2,TIMD4,VAV1 |
| Th2 Pathway | 9.12E-06 | 0.088 | HLA-DMB,HLA-DOA,HLA-DOB,IL12B,IL33,ITGB2,NOTCH2,PIK3CD,SPI1,TGFBR2,TIMD4,VAV1 |
| IL-17 Signaling | 4.90E-05 | 0.070 | CSF3,CXCL3,IL12B,IL1A,IL1B,IL33,LCN2,LIF,NOS2,PDGFC,PIK3CD,PTGS2,TNFSF13 |
| Differential Regulation of Cytokine Production in Intestinal Epithelial Cells by IL-17A and IL-17F | 5.50E-05 | 0.217 | CSF3,IL12B,IL1A,IL1B,LCN2 |
| FcγRIIB Signaling in B Lymphocytes | 7.41E-05 | 0.107 | BLNK,BTK,CD79A,CD79B,FCGR2B,INPP5D,PIK3CD,SYK |
| Toll-like Receptor Signaling | 8.13E-05 | 0.105 | IL12B,IL1A,IL1B,IL1RN,IL33,TLR4,TLR7,TLR9 |
| Th1 Pathway | 8.71E-05 | 0.083 | HLA-DMB,HLA-DOA,HLA-DOB,IL10RA,IL12B,IL18R1,ITGB2,NOTCH2,PIK3CD,VAV1 |
| Virus Entry via Endocytic Pathways | 1.20E-04 | 0.088 | CD55,FLNC,ITGA2,ITGA5,ITGB2,ITGB4,ITGB6,PIK3CD,RAC2 |
| Acute Phase Response Signaling | 1.41E-04 | 0.067 | C3,C4A/C4B,CFB,IL1A,IL1B,IL1RN,IL33,NGFR,PIK3CD,SERPINF1,TF,TNFRSF1B |
| Neuregulin Signaling | 1.48E-04 | 0.086 | AREG,BTC,DCN,ERBB2,ITGA2,ITGA5,ITGB2,ITGB4,MYC |
| PPAR Signaling | 1.48E-04 | 0.086 | IL1A,IL1B,IL1RN,IL33,NGFR,PDGFB,PDGFC,PTGS2,TNFRSF1B |
| Regulation Of The Epithelial Mesenchymal Transition By Growth Factors Pathway | 2.14E-04 | 0.064 | ERBB2,FGFR2,FGFR3,NGFR,PDGFB,PDGFC,PIK3CD,SMAD3,TGFBR2,TNFRSF1B,TNFSF13,VIM |
| HMGB1 Signaling | 2.69E-04 | 0.067 | IL12B,IL1A,IL1B,IL33,LIF,NGFR,PIK3CD,RAC2,TLR4,TNFRSF1B,TNFSF13 |
| IL-10 Signaling | 3.16E-04 | 0.100 | FCGR2A,FCGR2B,IL10RA,IL1A,IL1B,IL1RN,IL33 |
| LPS/IL-1 Mediated Inhibition of RXR Function | 3.16E-04 | 0.058 | ABCC3,APOE,FABP5,GSTA3,GSTO1,HS3ST1,IL1A,IL1B,IL1RN,IL33,NGFR,TLR4,TNFRSF1B |
| IL-15 Production | 4.27E-04 | 0.074 | BTK,CSF1R,EPHA1,EPHA2,ERBB2,FGFR2,FGFR3,FGR,SYK |
| HIF1α Signaling | 4.68E-04 | 0.059 | CYBB,EDN1,HK2,IGF1,MMP19,MMP7,NOS2,PDGFB,PDGFC,PIK3CD,TF,VIM |
| Role of Cytokines in Mediating Communication between Immune Cells | 4.79E-04 | 0.111 | CSF3,IL12B,IL1A,IL1B,IL1RN,IL33 |
| B Cell Development | 5.01E-04 | 0.139 | CD79A,CD79B,HLA-DMB,HLA-DOA,HLA-DOB |
| PI3K/AKT Signaling | 6.76E-04 | 0.060 | CSF2RB,IL10RA,IL18R1,INPP5D,ITGA2,ITGA5,ITGB2,ITGB4,PIK3CD,PTGS2,SFN |

## ESM Table 5.

## List of significant gene ontology terms identified for differentially expressed mRNAs

| Supplementary table S5: List of significant gene ontology terms identified for differentially expressed mRNAs | | | |
|---|---|---|---|
| Gene ontology term | p-value | Number of genes | List of genes |
| exocytosis | 6.81E-04 | 19 | Anxa3\|Atp2a2\|Btk\|Cftr\|Fgr\|Itgam\|Itgb2\|Lgals9\|Anxa1\|P2rx1\|Rac2\|Rest\|Syk\|Sdc1\|Tnfaip2\|Wnt7a\|Lat2\|Rab3il1\|Rims4 |
| regulation of regulated secretory pathway | 2.80E-03 | 11 | Atp2a2\|Fgr\|Itgam\|Itgb2\|Lgals9\|P2rx1\|Rac2\|Rest\|Syk\|Wnt7a\|Rims4 |
| protein secretion | 4.72E-06 | 28 | Apoe\|Cckar\|Cd22\|Cftr\|Csf1r\|Fgr\|Gja5\|Igf1\|Il12b\|Il1a\|Il1b\|Il1rn\|Itgb6\|Kcnn4\|Kcnq1\|Anxa1\|Nos2\|Rest\|Syk\|Tlr4\|Trh\|Clec4e\|Clec4n\|Il33\|Tlr9\|Ano1\|C1qtnf5\|Fermt1 |
| regulation of vesicle-mediated transport | 5.46E-07 | 33 | Apoe\|Arc\|Atp2a2\|C3\|Anxa2\|Cd22\|Cdh13\|Cftr\|Cnn2\|Cyba\|Fcgr2b\|Fcgr3\|Fgr\|Il1b\|Itga2\|Itgam\|Itgb2\|Lgals3\|Lgals9\|Anxa1\|P2rx1\|Rac2\|Rest\|Rit2\|Sftpd\|Syk\|Sdc1\|Trf\|Vtn\|Wnt7a\|Stap1\|Nckap1l\|Rims4 |
| endocytosis | 6.06E-07 | 39 | Anxa3\|Apoe\|Arc\|C3\|Anxa2\|Cd22\|Cd9\|Cdh13\|Cnn2\|Dmbt1\|Cyba\|Fcgr2b\|Fcgr3\|Fgr\|Il10ra\|Il1b\|Itga2\|Itgam\|Itgb2\|Lgals3\|Anxa1\|Rac2\|Rit2\|Msr1\|Sftpd\|Syk\|Tgfbr2\|Tgm2\|Tlr4\|Trf\|Vav1\|Vtn\|Stap1\|Ifitm3\|Epn3\|Nckap1l\|Colec12\|Rin1\|Rab7b |
| phagocytosis | 2.64E-06 | 23 | Anxa3\|C3\|Cnn2\|Cyba\|Fcgr2b\|Fcgr3\|Fgr\|Il1b\|Itga2\|Itgam\|Itgb2\|Anxa1\|Rac2\|Msr1\|Sftpd\|Syk\|Tgm2\|Tlr4\|Vav1\|Stap1\|Nckap1l\|Colec12\|Rab7b |
| antigen processing and presentation | 8.63E-04 | 9 | Ctse\|Ctss\|Fcgr2b\|Fcgr3\|H2-DMb1\|H2-Oa\|H2-Ob\|Rab32\|Wdfy4 |
| calcium-mediated signaling | 2.10E-03 | 12 | Atp2a2\|Cxcr5\|Cd22\|Cdh13\|Edn1\|Gsto1\|Igf1\|Rit2\|Syk\|Homer2\|Lat2\|Adgrb2 |
| protein processing | 6.83E-04 | 14 | C3\|Ctse\|Ctsh\|Ctss\|Cd55\|F3\|Il1b\|Mme\|Myc\|Klk1b4\|Klk1b3\|Plau\|Fcmr\|Nlrc4 |
| positive regulation of oxidative stress-induced cell death | 9.43E-03 | 3 | Itgam\|Rest\|Tlr4 |
| superoxide anion generation | 9.45E-08 | 9 | Acp5\|Cyba\|Cybb\|Edn1\|Itgam\|Itgb2\|Syk\|Hvcn1\|Duox2 |
| cell killing | 3.02E-10 | 22 | C3\|Ctsc\|Ctsh\|Cd55\|Fcer2a\|Fcgr3\|Il12b\|Itgam\|Lgals3\|Lgals9\|Ltf\|Nos2\|Reg3g\|Cxcl2\|Cxcl5\|Syk\|Pglyrp1\|Vav1\|Klrk1\|Stap1\|Hamp2\|Raet1e |
| reactive oxygen species metabolic process | 3.73E-09 | 25 | Acp5\|Cryab\|Cyba\|Cybb\|Cyp1b1\|Edn1\|Hk2\|Igf1\|Il1b\|Itgam\|Itgb2\|Smad3\|Ngfr\|Nos2\|Pdgfb\|Plau\|Ptgs2\|Syk\|Tgfbr2\|Tlr4\|Vav1\|Klrk1\|Duoxa2\|Hvcn1\|Duox2 |
| regulation of secretion by cell | 3.87E-09 | 43 | Apoe\|Atp2a2\|Runx1\|Cckar\|Cd22\|Cftr\|Csf1r\|Edn1\|Fgr\|Gal\|Gja5\|Igf1\|Il12b\|Il1a\|Il1b\|Il1rn\|Itgam\|Itgb2\|Kcnn4\|Kcnq1\|Lgals3\|Lgals9\|Lif\|Anxa1\|Ngf\|Nos2\|P2rx1\|Rac2\|Rest\|Syk\|Sdc1\|Tlr4\|Tnfrsf1b\|Trh\|Wnt7a\|Clec4e\|Clec4n\|Il33\|Tlr9\|Ano1\|Fermt1\|Rims4\|Rasl10b |
| stress-activated protein kinase signaling cascade | 6.37E-03 | 13 | Cryab\|Edn1\|Fcgr2b\|Fgfr3\|Il1a\|Il1b\|Il1rn\|Syk\|Tlr4\|Trf\|Wnt7a\|Wnt7b\|Tlr9 |
| actin cytoskeleton organization | 1.16E-06 | 36 | Avil\|Arhgdib\|Capg\|Casp4\|Cckar\|Cnn2\|Csf1r\|Csf3\|Edn1\|Eln\|Epha1\|Fgr\|Fhl3\|S1pr2\|Gpm6b\|Hcls1\|Il1a\|Krt19\|Stmn1\|Anxa1\|Smad3\|Notch2\|Pdgfb\|Rac2\|Trf\|Xirp1\|Amot\|Pdlim1\|Tacstd2\|Stap1\|Inf2\|Cdc42ep1\|Nckap1l\|Myo5c\|Frmd5\|Arhgap28 |
| apoptotic signaling pathway | 1.28E-04 | 28 | Casp4\|Ctsc\|Ctsh\|Cyp1b1\|Epha2\|Fgfr3\|Gdnf\|Ifi204\|Igf1\|Il1a\|Il1b\|Itgam\|Lcn2\|Lgals3\|Smad3\|Mal\|Myc\|Ngf\|Ngfr\|Plaur\|Ptgs2\|Tnfrsf1b\|Vnn1\|Sfn\|Fcmr\|Dapl1\|Il33\|Cth |
| glucose transmembrane transport | 9.35E-03 | 7 | C3\|Edn1\|Hk2\|Igf1\|Il1b\|Fabp5\|Myc |
| endocrine pancreas development | 1.96E-03 | 6 | Cdk6\|Cftr\|Anxa1\|Ptf1a\|Sox9\|Onecut2 |
| small GTPase mediated signal transduction | 4.48E-04 | 21 | Apoe\|Arhgdib\|Cdh13\|Erbb2\|Pdpn\|Igf1\|Stmn1\|Klk1b4\|Ngf\|Ngfr\|Notch2\|Rac2\|Rit2\|Vav1\|Amot\|Fgd3\|Eps8l1\|Cyth4\|Cdc42ep1\|Arhgap30\|Arhgap28 |
| inflammatory response | 4.00E-27 | 71 | Acp5\|Adam8\|Apoe\|Btk\|Ciita\|C3\|C3ar1\|Casp4\|Cebpa\|Cnr2\|Csf1r\|Ctsc\|Ctss\|Cyba\|Cybb\|Cd55\|Ecm1\|Epha2\|F3\|Fcgr2b\|Fcgr3\|Gal\|Lilrb4a\|Hyal1\|Igf1\|Il12b\|Il16\|Il1a\|Il1b\|Il1rn\|Il18r1\|Itga2\|Itgam\|Itgb2\|Itgb6\|Lgals9\|Anxa1\|Ly86\|Smad3\|Naip6\|Nos2\|Notch2\|P2rx1\|Reg3b\|Pik3cd\|Ptgs2\|Reg3g\|Ccl6\|Ccl7\|Ccl9\|Cxcl2\|Cxcl5\|Serpinf1\|Syk\|Sdc1\|Tgm2\|Tlr4\|Tnfrsf1b\|Pglyrp1\|Vnn1\|Stap1\|Serpinb1a\|Duoxa2\|Sting1\|Il33\|Tlr9\|Pik3ap1\|Tlr7\|Metrnl\|Themis2\|Nlrc4 |
| regulation of cytokine production | 1.26E-17 | 57 | Acp5\|Adam8\|Btk\|C3\|C3ar1\|Casp4\|Runx1\|Csf1r\|Cyba\|Cybb\|Cyp1b1\|Epha2\|F3\|Fcgr2b\|Fcgr3\|Fgr\|Ifi204\|Igf1\|Il12b\|Il16\|Il1a\|Il1b\|Il18r1\|Inpp5d\|Itgb6\|Lgals9\|Anxa1\|Ltf\|Smad3\|Nos2\|Pou2af1\|Ptgs2\|Cxcl5\|Sftpd\|Syk\|Tlr4\|Tnfrsf1b\|Pglyrp1\|Homer2\|Klrk1\|Clec4e\|Clec4n\|Serpinb1a\|Mcoln2\|Sting1\|Il33\|Tlr9\|Trim16\|Nckap1l\|Tlr7\|Rab7b\|Sulf1\|Fermt1\|Nlrc4\|Ltbp1\|Ifi211\|Isg15 |
| positive regulation of cytokine production | 2.99E-13 | 39 | Adam8\|C3\|C3ar1\|Casp4\|Runx1\|Csf1r\|Cyba\|Cybb\|Cyp1b1\|F3\|Fcgr3\|Fgr\|Ifi204\|Il12b\|Il16\|Il1a\|Il1b\|Il18r1\|Lgals9\|Anxa1\|Smad3\|Pou2af1\|Ptgs2\|Syk\|Tlr4\|Klrk1\|Clec4e\|Clec4n\|Mcoln2\|Sting1\|Il33\|Tlr9\|Trim16\|Tlr7\|Rab7b\|Sulf1\|Fermt1\|Nlrc4\|Ifi211 |

Gene ontology terms highlighted in yellow indicate the terms selected for representation

**ESM Table 6.**
**Checklist for reporting human islet preparations used in research**

| Islet preparation | 1 | 2 | 3 | 4 | 5 | 6 | 7 | 8[a] |
|---|---|---|---|---|---|---|---|---|
| MANDATORY INFORMATION | | | | | | | | |
| Unique identifier | AEL5345 (*SAMN08611141*) | AFA3256 (*SAMN08611211*) | AEIY348 (SAMN08768997) | AEIL428A (*SAMN08768972*) | | | | |
| Donor age (years) | 48 | 45 | 24 | 27 | | | | |
| Donor sex (M/F) | M | M | M | M | | | | |
| Donor BMI (kg/m$^2$) | 32.4 | 29.8 | 38.1 | 30 | | | | |
| Donor $HbA_{1c}$ or other measure of blood glucose control | 5.6 | 5.1 | 5.4 | 5.4 | | | | |
| Origin/source of islets[b] | IIDP program | IIDP program | IIDP program | IIDP program | | | | |
| Islet isolation centre | *The Scharp-Lacy Research Institute* | *The Scharp-Lacy Research Institute* | *Southern California Islet Cell Resource Center* | *University of Wisconsin* | | | | |
| Donor history of diabetes? Please select yes/no from drop down list | No | No | No | No | | | | |
| If Yes, complete the next two lines if this information is available | | | | | | | | |
| Diabetes duration (years) | | | | | | | | |
| Glucose-lowering therapy at time of death[c] | | | | | | | | |
| RECOMMENDED INFORMATION | | | | | | | | |
| Donor cause of death | | | *Cerebrovascular/stroke* | *Head trauma* | | | | |
| Warm ischaemia time (h) | | | | *Yes, 32 minutes* | | | | |

| | | | | | | | | |
|---|---|---|---|---|---|---|---|---|
| Cold ischaemia time (h) | | | | *4 Hour(s) 20 Minute(s)* | | | | |
| Estimated purity (%) | | | | *95%* | | | | |
| Estimated viability (%) | | | | *99%* | | | | |
| Total culture time (h)[d] | | | | | | | | |
| Glucose-stimulated insulin secretion or other functional measurement[e] | | | | | | | | |
| Handpicked to purity? Please select yes/no from drop down list | | | | | | | | |
| Additional notes | | | | | | | | |

Adapted from Hart NJ, Powers AC (2018) Progress, challenges, and suggestions for using human islets to understand islet biology and human diabetes. Diabetologia https://doi.org/10.1007/s00125-018-4772-2

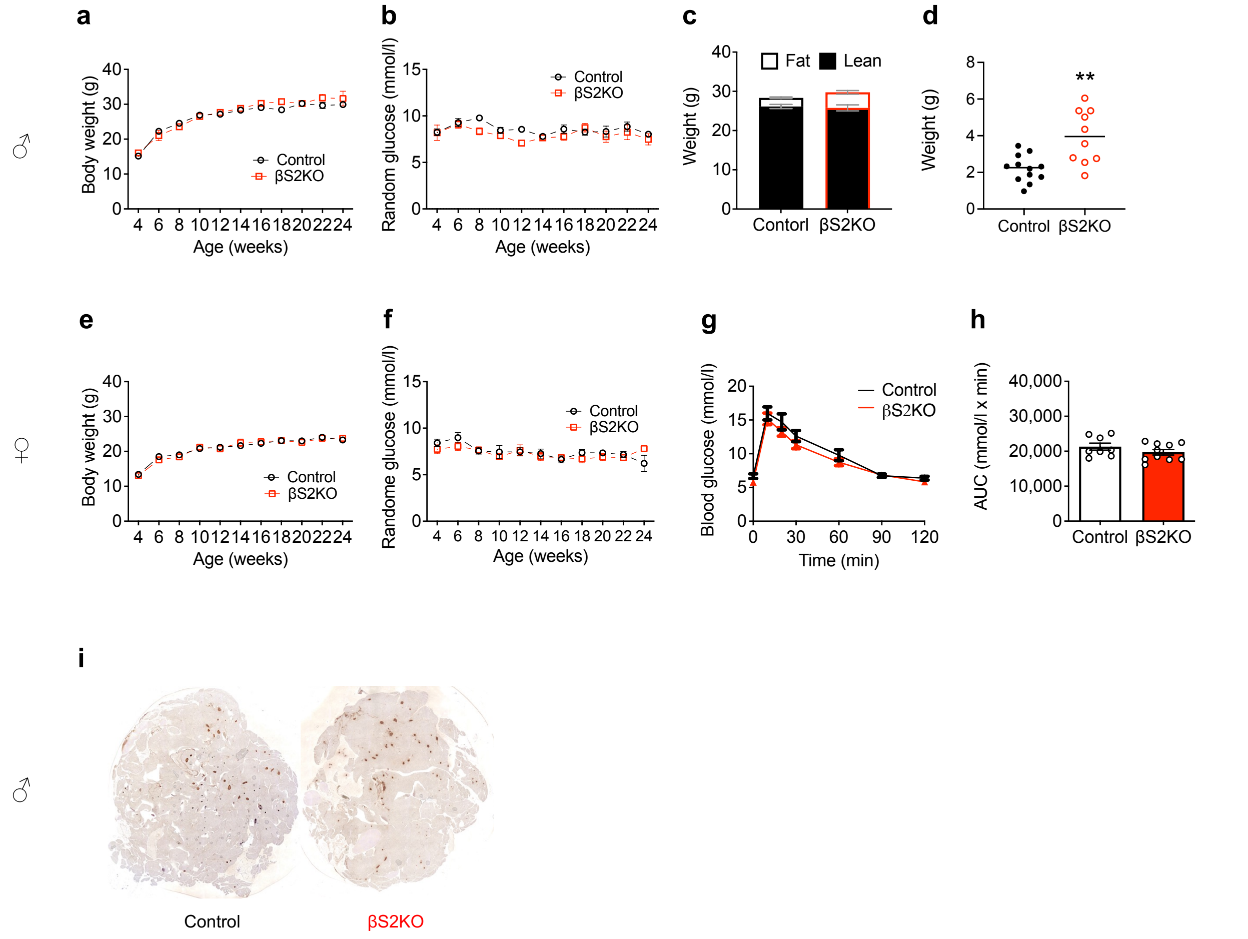


**ESM Fig. 1. SERCA2 deficiency did not impact weight, lean mass, or glucose tolerance in female mice.**

Beta cell-specific SERCA2 KO (βS2KO) and SERCA2$^{flox/flox}$ mice (Control) were fed a normal chow diet for 25 weeks. Longitudinal changes in whole body weight and random blood glucose in male (A and B) and female mice (E and F) was monitored until 24-week-old, n= at least 6.

(C-D) Lean mass and fat mass were measured in male mice at 25 weeks of age using the EchoMRI 500 Body Composition Analyzer, n=10-12.

(G-H) GTT was performed (2 g/kg glucose dosed to lean mass) in female mice at 24 weeks of age. Area under curve (AUC) analysis is shown graphically (H), n=8-10.

(I) Representative images of insulin immunostaining in pancreatic sections from male control and βS2KO mice at 25 weeks-of-age.

Results are displayed as mean ± SEM. *p < 0.05; **p < 0.01 vs control.

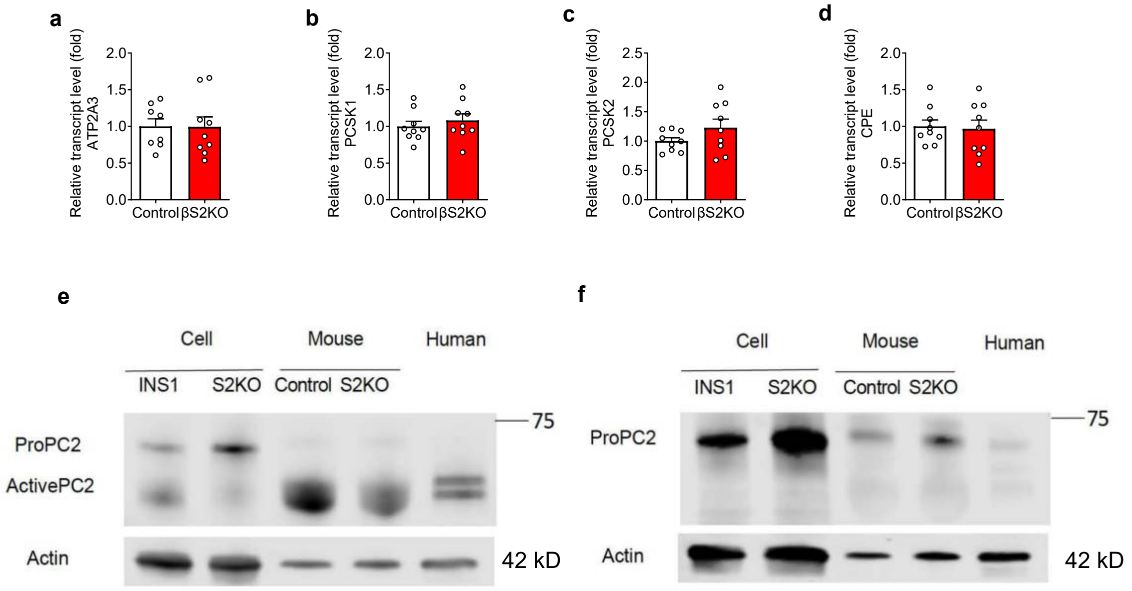


**ESM Fig. 2. Beta cell-specific SERCA2 deficiency did not alter transcription of prohormone convertase isoforms in islets.**

(A-D) Transcript levels of *ATP2A3*, *PCSK1*, *PCSK2*, and *CPE* in islets of control and βS2KO mice (male, 24-weeks-of-age) were determined by RT-qPCR and normalized to actin levels. Replicates are indicated by open circles and squares. *Indicates statistically significant difference from control (***p<0.001) by a Student's *t*-test.

(E-F) Protein lysate from INS1 cells, isolated mouse islets, and human islets were used for western blot analysis. Representative immunoblots of PC2 (E) and ProPC2 (F) with Actin blot, which is the loading control. PC2 propeptide antiserum (LSU26) was raised against residues $His^{58}$-$Asp^{80}$ of mouse pro-PC2, as described in the Methods.

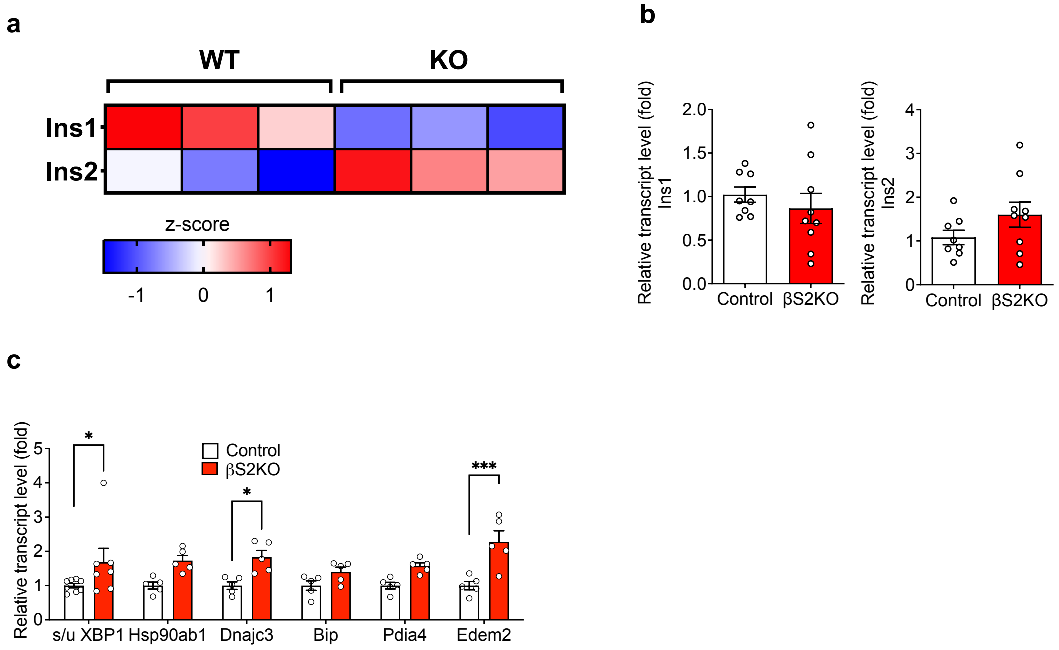


**ESM Fig. 3. Markers of ER stress were mildly increased, while insulin gene mRNA levels were not altered in islets isolated from βS2KO mice.**

(A) RNA isolated from control and βS2KO islets was subjected to bulk RNA sequencing analysis at 17-weeks of age. Heatmap of the fold changes for insulin genes.

(B) Transcript levels of *Ins1* and *Ins2* in islets of control and βS2KO mice (male, 24 weeks of age) were determined by RT-qPCR and normalized to actin levels. Replicates are indicated by open circles and squares. ns = not significantly different between groups.

(C) Transcript levels of spliced and unspliced *Xbp1, Hsp90ab1, Dnajc3, Bip* (also known as *Hspa5*), *Pdja4,* and *Edem2* were determined by RT-qPCR and normalized to actin levels. Replicates are indicated by open circles and squares. Results are presented as the mean ± S.E.M. *Indicates statistically significant difference (*p<0.05, ***p<0.001) by a student t-test.

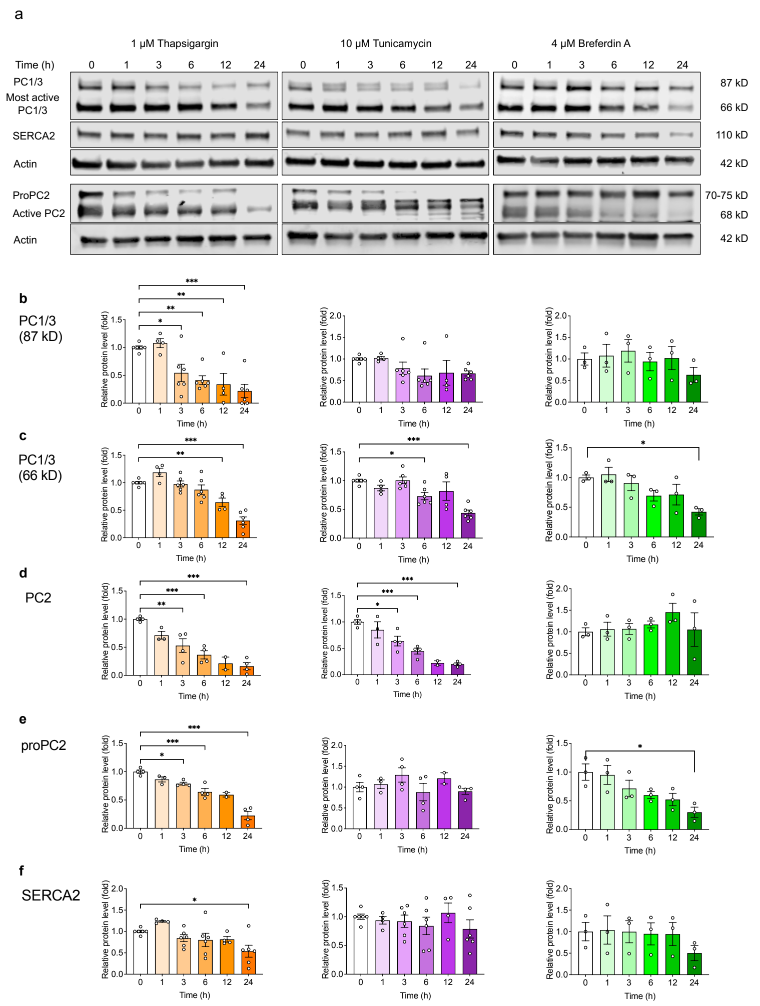


**ESM Fig. 4. ER stress and inhibition of protein trafficking reduced active prohormone convertases isoforms in beta cell lines.**

(A) Representative immunoblots of PC1/3, SERCA2, and PC2 in INS1 cells treated with 1 umol/L thapsigargin, 10 u mol/L tunicamycin, or 4 u mol/L brefeldin A for 0, 1, 3, 6, 12, or 24 h.

(B-F) Quantitation of immunoblotting results. Expression of proteins was normalized to actin expression; n=2-6. Results are presented as the mean ± S.E.M. Replicate samples are indicated by open circles. *Indicates statistically significant difference (*p<0.05, **p<0.01, ***p<0.001) by a two-way ANOVA and Sidak's post-test.

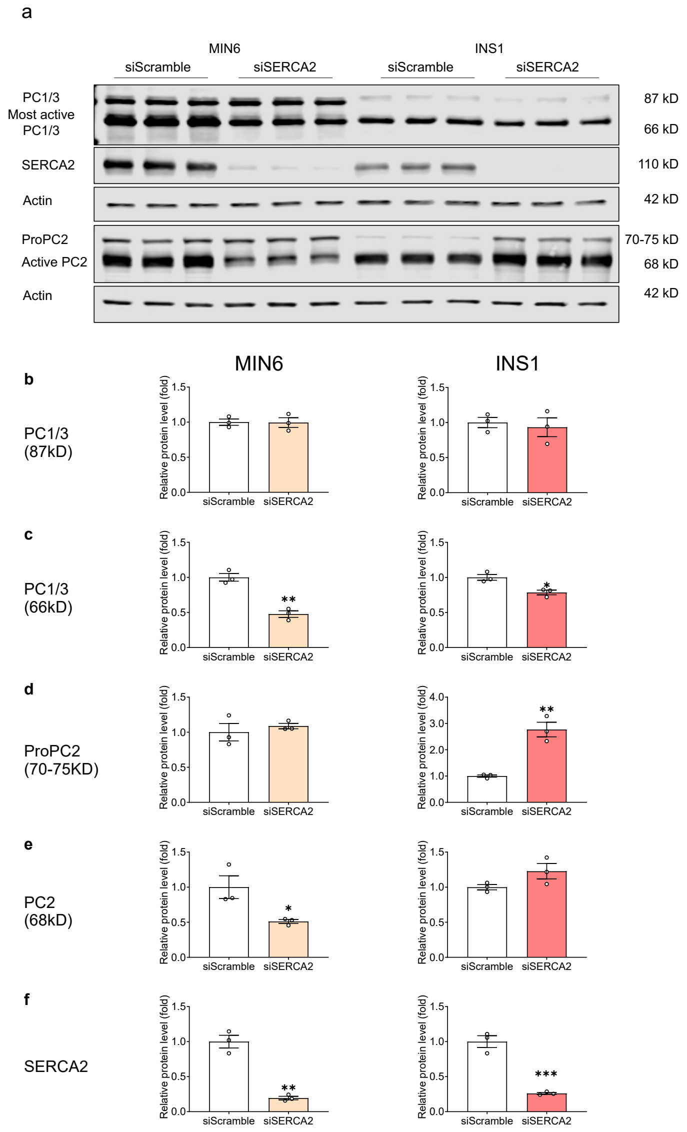


**ESM Fig. 5. siRNA-based SERCA2 knockdown reduced active prohormone convertases isoforms in beta cell lines.**

(A) Representative immunoblots of PC1/3, SERCA2, and PC2 in MIN6 or INS1 cells treated with siSERCA2 or control siRNA for 48 h.

(B-F) Quantitation of immunoblotting results. Expression of proteins was normalized to actin expression; n=3. Results are presented as the mean ± S.E.M. Replicate samples are indicated by open circles.

*Indicates statistically significant difference (*p<0.05, **p<0.01, ***p<0.001) by a student t-test.
